# Medicinal chemistry breakthroughs on ATM, ATR, and DNA-PK inhibitors as prospective cancer therapeutics

**DOI:** 10.1080/14756366.2025.2489720

**Published:** 2025-04-21

**Authors:** Ram Sharma, Anshul Mishra, Monika Bhardwaj, Gurpreet Singh, Larasati Vanya Indira Harahap, Sakshi Vanjani, Chun Hsu Pan, Kunal Nepali

**Affiliations:** a School of Pharmacy, College of Pharmacy, Taipei Medical University, Taipei, Taiwan; b Institute of Biological Chemistry, Academia Sinica, Taipei, Taiwan; c Department of Pharmaceutical Chemistry, ISF College of Pharmacy, Moga, India; d Molecular Medicine, University of South Florida, Tampa, FL, USA; e Ph.D. Program in Drug Discovery and Development Industry, College of Pharmacy, Taipei Medical University, Taipei, Taiwan

**Keywords:** Ataxia Telangiectasia Mutated, ATM and Rad3-related kinase, DNA-PK, DNA damage, cancer

## Abstract

This review discusses the critical roles of Ataxia Telangiectasia Mutated Kinase (ATM), ATM and Rad3-related Kinase (ATR), and DNA-dependent protein kinase **(**DNA-PK) in the DNA damage response (DDR) and their implications in cancer. Emphasis is placed on the intricate interplay between these kinases, highlighting their collaborative and distinct roles in maintaining genomic integrity and promoting tumour development under dysregulated conditions. Furthermore, the review covers ongoing clinical trials, patent literature, and medicinal chemistry campaigns on ATM/ATR/DNA-PK inhibitors as antitumor agents. Notably, the medicinal chemistry campaigns employed robust drug design strategies and aimed at assembling new structural templates with amplified DDR kinase inhibitory ability, as well as outwitting the pharmacokinetic liabilities of the existing DDR kinase inhibitors. Given the success attained through such endeavours, the clinical pipeline of DNA repair kinase inhibitors is anticipated to be supplemented by a reasonable number of tractable entries (DDR kinase inhibitors) soon.

## Introduction

The genetic material within a cell is continuously threatened by chemical modifications, primarily induced by exotic components for example, reactive oxygen species (ROS), ionising radiation (IR), UV radiation, and others. As a result, DNA is highly vulnerable to diverse varieties of damage, including breakage of DNA double-strand, single-strand, and base modifications such as alkylation and oxidation. To maintain genetic stability and ensure normal cellular function, cells have amplified numerous mechanisms for the reconstruction of damaged DNA^1–3^. Literature precedents claim that various kinases have a central role in cellular reactions to DNA damage by orchestrating the revealing, signalling, and repair of damage to maintain genomic integrity. Key kinases such as ATM (Ataxia Telangiectasia Mutated), ATR (ATM and Rad3-related), and DNA-PK (DNA-dependent Protein Kinase) are central to DNA damage response^4–14^. ATM kinase is primarily triggered by double-strand breaks and coordinates repair through homologous recombination pathways through phosphorylating critical substrates like p53 and CHK2. ATR, activated by replication stress and single-strand DNA, stabilizes halted replication forks and manages repair via CHK1. DNA-PK, essential for non-homologous end joining (NHEJ), processes and ligates DNA terminus at double-strand breaks^15–24^. Other kinases, for instance, CHK1 and CHK2, maintain cell cycle checkpoints to avert progression before repair is complete, while CDKs (Cyclin-dependent Kinases) and PLK1 (Polo-like Kinase 1) modulate cell cycle in response to damage or recovery. Additionally, stress-activated kinases like MAPKs influence apoptosis and Aurora kinases ensure mitotic processes halt until DNA integrity is restored. These kinases not only preserve cell survival by facilitating repair but also trigger apoptosis if damage is irreparable^25–33^. Despite these repair processes, genomic alterations can still occur, leading to DNA damage and modifications in cellular instructions key contributors to cancer development^34^. Genomic alterations, such as amplifications, deletions, or mutations in tumour suppressor genes or oncogenes, play a fundamental role in cancer progression. DNA damage is regarded as one of the critical hallmarks of cancer, as cancer cells exhibit elevated levels of genomic variability and abnormal DNA damage^35^^,^^36^. Mutational selection pressure on proto-oncogenes, tumour suppressor genes, and regions of the genome affected by defective DNA repair mechanisms participate in the conversion of normal cells into tumour cells^37^. The stimulation of DNA restoration genes and the downregulation of DNA relocalization genes present opportunities for targeted cancer therapies, particularly through multitarget anticancer drugs that simultaneously inhibit repair pathways and target oncogenic pathways^38^.

The three key DNA repair pathways ATM, ATR, and DNA-PK act as core monitors of DNA damage response, significantly influencing tumour development and treatment outcomes. These pathways influence vital cellular functions, including cell cycle regulation, cell death, gene expression, oxidative stress management, and telomere stability. The intricate involvement of DNA repair pathways in the aforementioned processes highlights their potential as therapeutic targets^39^. However, advancing their clinical applications requires a deeper understanding to optimise therapeutic benefits while minimising the risks associated with their inhibition.

This review discusses the roles of three major kinases ATM, ATR, and DNA-PK in sensing DNA damage and catalysing DNA repair, as well as their involvement in cancer. The intricate interplay between these kinases, highlighting their collaborative and distinct roles in maintaining genomic integrity and promoting tumour development under dysregulated conditions has been discussed. Moreover, recent advances in the field of DNA damage response kinase inhibitor discovery at clinical and preclinical levels have been covered. To add on, the outcomes of the recently conducted medicinal chemistry campaigns in the context of structure-activity relationship, and pharmacokinetic and pharmacodynamic profiling of new structural assemblages have been presented. Noteworthy to mention that the drug discovery endeavours employed robust drug design strategies and culminated into a plethora of tractable ATM/ATR/DNA-PK inhibitors that on exhaustive explorations might emerge as cancer therapeutics in the long run.

## Ataxia telangiectasia mutated (ATM)

### Structural and mechanism of ATM kinase activation

**Figure 1.**
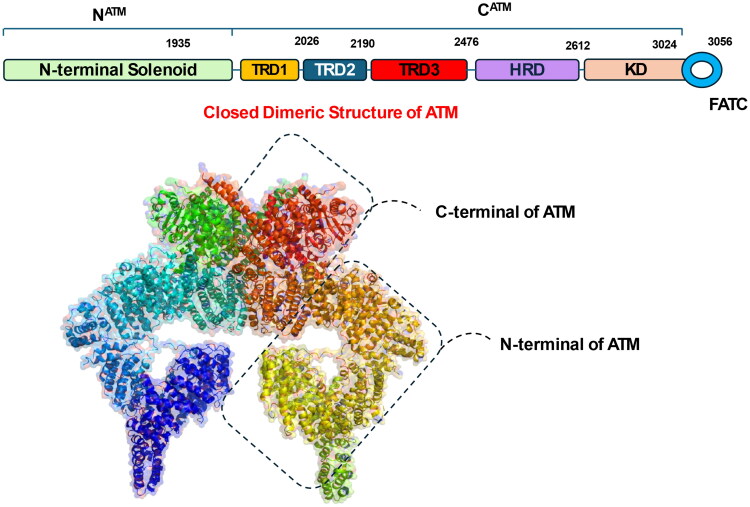
Closed dimeric structure of Ataxia Telangiectasia Mutated (ATM) (the figure was drawn by the authors using BioRender software).

The Ataxia Telangiectasia Mutated (ATM) gene is a key governing kinase of DNA damage response, particularly in addressing damage induced by environmental stressors. It encodes a widely expressed protein kinase that is present across all human tissues. Structurally, the ATM protein contains an amino-terminal domain with phosphoinositide 3-kinase-like activity, a large central region, and a carboxyl-terminal FATC domain^40^^,^^41^. Figure 1 illustrates the cryo-EM structure of the ATM kinase in its dimeric form, representing an autoinhibited state, critical for its regulation. In the dimeric state, two ATM molecules are symmetrically arranged, forming a “butterfly” architecture. This arrangement is stabilised by extensive interactions between specific regions of the protein, particularly FAT (FRAP-ATM-TRRAP), KD, and FATC (C-terminal FAT) sections. The structure reveals that dimerisation is mediated by interactions within the C^ATM^ region (comprising FAT, KD, and FATC) of each monomer. Specifically, the TRD3-DH helices (tetratricopeptide repeat domain 3 dimeric helices) form a key structural element, stabilising the dimer and maintaining the kinase in an autoinhibited conformation. This inhibition arises from the positioning of the PRD (PIKK regulatory domain) and its interactions with the TRD3-DH helices and the KD, which restrict access to the catalytic pocket. Moreover, In the dimeric state, the catalytic pocket is narrower and less accessible than in the monomeric state, limiting the enzyme’s activity^42^^,^^43^.

**Figure 2.**
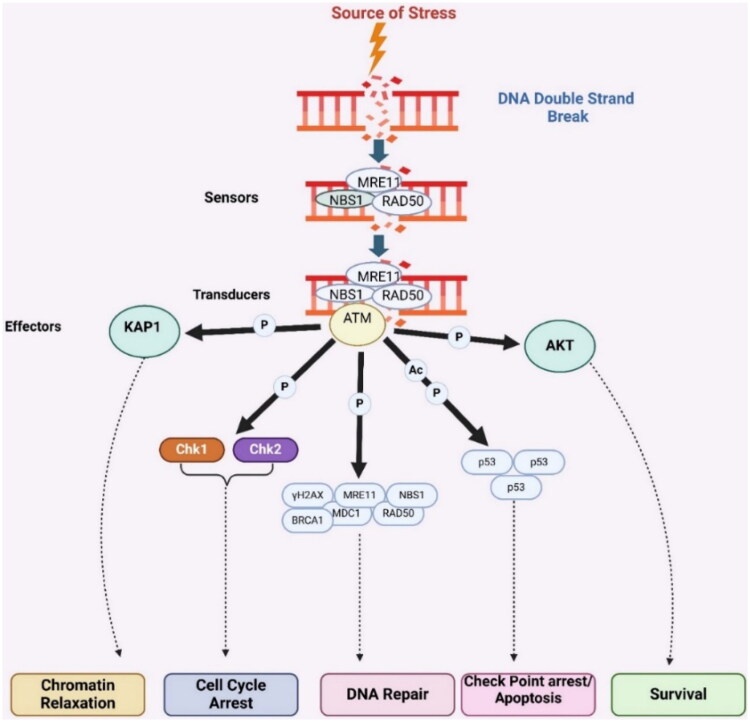
Mechanism of ATM kinase activation in DNA double strand (the figure was drawn by the authors using BioRender software).

Although ATM dimers have less accessible catalytic pockets, activated and quickly enrolled into DNA double-strand break (DSB) sites. This recruitment triggers autophosphorylation at Ser1981 (based on human ATM numbering), facilitating their transition to monomers. Upon monomerization, ATM activates several essential targets through phosphorylation, for example, histone H2AX, p53, also CHK2. Phosphorylated H2AX (γ-H2AX) acts as a signal to recruit various proteins involved in the repair of double-strand breakdown. One of the key complexes recruited is MRE11-RAD50-NBS1 (MRN) complex, which exhibits both exonuclease and endonuclease activities (Figure 2)^44–49^.

### Role of ATM in cancer development

ATM kinase is an oncogene silencer with a multifaceted role in cancer progression and genomic stability^50^. As a fundamental regulator of the DNA damage response. ATMs primarily respond to double-strand breaks caused by exotic components like radiation, chemotherapy, oxidative stress, or biological progressions for instance V(D)J recombination and meiosis^51–54^. It stimulates p53 and regulates cell cycle checkpoints, facilitating DNA repair or triggering natural cell death to inhibit the proliferation of cells^55–57^. The significance of ATM’s function is exemplified by ataxia-telangiectasia (A-T), an autosomal recessive disorder after homozygous ATM mutations. A-T cells fail to enforce G1/S and G2/M checkpoints under genotoxic stress, leading to unchecked replication of damaged DNA and an increased mutation burden. Patients with A-T exhibit profound radiosensitivity, cerebellar ataxia, oculocutaneous telangiectasias, immunodeficiency, and a significantly elevated risk of malignancies, particularly haematologic cancers, such as mantle cell lymphoma and T-cell prolymphocytic leukaemia, frequently harbour ATM mutations, with biallelic inactivation occurring in approximately 50% of cases^58–60^. Multiple reports indicate that Individuals carrying heterozygous mutations in the ATM gene exhibit an elevated susceptibility to breast cancer, particularly before the age of fifty. Moreover, somatic mutations in ATM are identified in approximately 4% of malignancies, with loss of heterozygosity occurring in nearly 40% of sporadic breast cancer cases. These oncogenic ATM aberrations, including truncating mutations, missense variants, and copy number deletions, collectively compromise genomic integrity, thereby promoting tumorigenesis^61–66^. Despite its established role as a tumour-down regulator, ATM’s contribution to cancer biology is nuanced, reflecting both protective and tumour-promoting effects. In early tumorigenesis, ATM is activated in response to replication stress and oncogene-driven DNA damage^67^. This activation functions as an initial barrier to malignant transformation by inducing p53-mediated cell cycle arrest, apoptosis, or senescence, thereby curbing the proliferation of genetically compromised cells. However, as neoplastic progression occurs, tumours frequently acquire mutations or functional impairments in DDR regulators, such as ATM and p53. These disruptions allow cells to bypass DDR-induced growth constraints, promoting genomic instability and facilitating the evolution of premalignant lesions into invasive carcinomas. This dual role of DDR in tumour suppression and adaptation underscores its complex contribution to oncogenesis. Research consistently highlights the role of upregulated ATM signalling in cancer resistance to therapies such as chemotherapy and radiation. These treatments encouraged DNA double-strand breaks (DSBs), but ATM activation enhances repair efficiency, promoting tumour survival^68^. For example, ATM-mediated Akt pathway activation supports pro-survival signalling and inhibits apoptosis, while NF-κB activation fosters cancer progression by enhancing survival, suppressing cell death, and driving epithelial-mesenchymal transition (EMT), facilitating cancer cell migration and metastasis. Beyond DNA repair, ATM influences tumour metabolism by activating the pentose phosphate pathway, which reduces reactive oxygen species and supports nucleotide biosynthesis and anabolic processes. This metabolic shift allows cancer cells to thrive under oxidative stress and maintain proliferation. Notably, in breast cancer with elevated HOXB9 expression or decreased PRSS11, ATM overactivity correlates with enhanced EMT and metastasis. ATM’s role extends to hypoxic tumour microenvironments, where it stabilises hypoxia-inducible factor 1-alpha (HIF1α). This stabilisation, mediated directly or through the TRAF6/H2AX/HIF1α axis, promotes angiogenesis, invasion, and metastasis, aiding tumour progression^69^^–^^77^. Another report demonstrated that in breast cancer, the antisense transcript ATM-AS positively regulates ATM expression by recruiting the KAT5 histone acetyltransferase to the ATM promoter. Reduced ATM-AS levels impair ATM-mediated DNA damage repair and correlate with poor prognosis, revealing a novel mechanism of ATM dysregulation in breast cancer^78^. Numerous studies have elucidated the pivotal roles of ATM and BRCA1/2 genes in cancer progression, therapeutic resistance, and immune modulation, highlighting their potential as therapeutic targets across diverse malignancies. In metastatic prostate cancer, aberrations in homologous recombination repair (HRR) genes, particularly BRCA1/2, are correlated with poorer overall survival outcomes^79^^,^^80^. Furthermore, ATM is critically implicated in the pathogenesis of myelodysplastic neoplasms (MDS) and their progression to acute myeloid leukaemia (AML). In MDS, ATM is frequently epigenetically silenced or downregulated through methylation or mutational events, thereby contributing to disease advancement. Elevated ATM methylation and reduced expression levels are observed in high-risk MDS and AML patients, underscoring ATM’s potential as a therapeutic target to mitigate AML transformation. ATM also exerts a significant influence on glucose metabolism in oncogene-driven cancers. Moreover, Pharmacological inhibition of ATM attenuates the expression of glycolytic enzymes and reduce oxidative phosphorylation levels, thereby augmenting apoptosis induced by oncogene-targeted therapies. This metabolic reprogramming suggests that combinatorial strategies involving ATM inhibitors and driver oncogene inhibitors could enhance therapeutic efficacy in cancers driven by oncogenic mutations. Additionally, ATM plays a crucial role in oncogene-induced malignant transformation. Sustained ATM activation facilitates transcriptional reprogramming and chromatin relaxation, enabling oncogenic transcription factors to access chromatin. Preclinical models demonstrate that ATM inhibition suppresses tumorigenesis, revealing its role in promoting, rather than suppressing, oncogene-driven transformation. ATM signalling also supports the differentiation of myofibroblastic cancer-associated fibroblasts, which are known to inhibit T-cell infiltration and antitumor immunity. Targeting ATM in tumours rich in myofibroblastic cancer-associated fibroblasts enhance CD8+ T-cell infiltration and improves responsiveness to immunotherapeutic interventions, providing a compelling rationale for combining ATM inhibitors with immune checkpoint blockade therapies. ATM inhibitors are specifically designed to disrupt the DNA damage response (DDR), impairing DNA repair mechanisms in cancer cells and inducing tumour cell death. In contrast, normal cells, which possess alternative DNA repair pathways, exhibit minimal susceptibility to ATM inhibition. Previous studies have demonstrated that ATM inhibitors potentiate cisplatin-induced apoptosis in breast cancer cells and exhibit synergistic effects with radiation therapy in glioblastoma. ATM inhibition sensitises cancer cells to DNA-damaging agents, PARP inhibitors, and immune checkpoint blockade by modulating DNA repair mechanisms and immune responses. For instance, ATM negatively regulates PD-L1 expression in triple-negative breast cancer (TNBC) via the JNK/c-Jun/TNF-α signalling axis, suggesting that ATM inhibition could enhance the efficacy of immune checkpoint therapy in PD-L1-negative TNBC patients. In testicular cancer, dual inhibition of ATM and histone deacetylase (HDAC) demonstrates potent antitumor activity, with favourable toxicity profiles and high efficacy in preclinical models^81–96^. These findings collectively highlight the therapeutic promise of targeting ATM in conjunction with other treatment modalities to enhance clinical outcomes across a spectrum of cancer types. Over the past two decades, significant research efforts have been directed towards the development of ATM inhibitors characterised by high selectivity and potent inhibitory activity. This has involved the exploration of diverse molecular scaffolds, culminating in the identification of multiple distinct classes of ATM inhibitors, each exhibiting unique pharmacodynamic and pharmacokinetic profiles. Here (Table 1), ATM inhibitors have been systematically categorised based on their structural frameworks and pharmacological activity for therapeutic applications in cancer.

**Table 1.** ATM inhibitors and their role in various cancers.

ATM inhibitor	Target	Type of cancer	Description	References
**Wortmannin (1)** 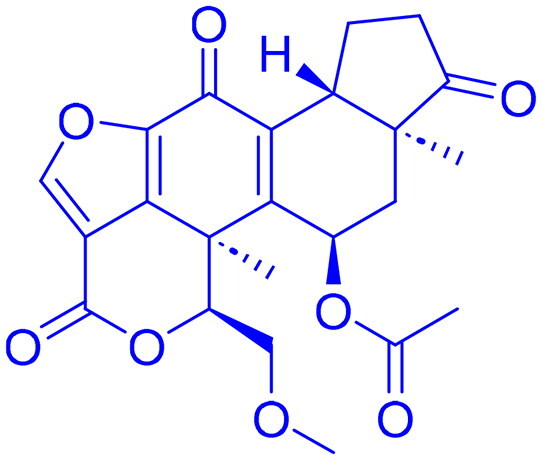	ATM, mTOR, DNA-PK, ATR	Lung cancer, breast cancer, leukaemia	**Wortmannin (1)** inhibits MCF-7 and K562 cell growth and promotes apoptosis by silencing the PI3K/Akt pathway and reducing NF-κB expression in a dose- and time-dependent manner.	^97–100^
**KU-55933 (2)** 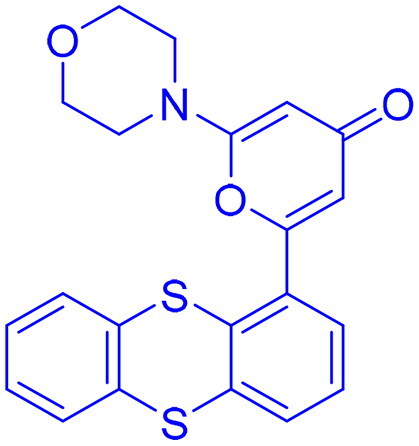	ATM	Breast cancer, prostate cancer, lung cancer	**KU55933 (2)** promotes cell death in breast & prostate cancer cell lines **KU55933 (2)** impedes Akt phosphorylation triggered by insulin and insulin-like growth factor I in cancer cells. Nanoparticles of **KU55933 (2)** have lower skin toxicity in Lung Cancer xenografts model.	^101^ ^,^ ^102^
**KU-60019 (3)** 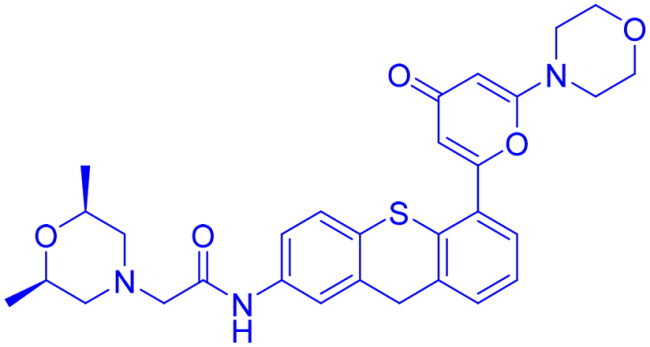	ATM	Lung cancer, breast cancer, lymphoma	**KU60019 (3)** synergistically exposes lung cancer cells to topoisomerase II by cell death and DNA double stand break. **KU60019 (3)** suppressed the proliferation & cell motility, and invasion of MCF-7 cells and significantly increased chemo sensitisation. **KU60019 (3)** induces cell cycle arrest & reduced expression of p21	^103^ ^,^ ^104^
**KU-59403 (4)** 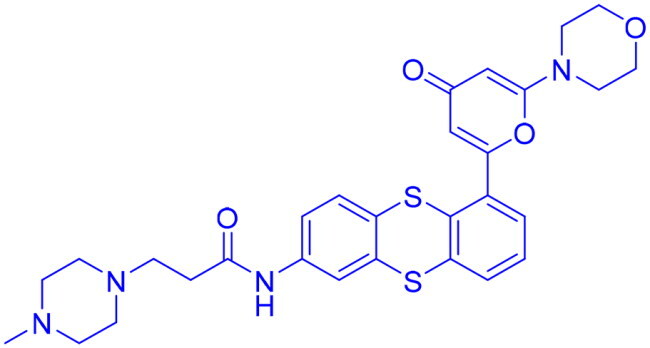	ATM	Colon cancer	**KU-59403 (4)** improved the anti-tumour effects of topoisomerase poisons in mouse models	^105^
**AZ31 (5)** 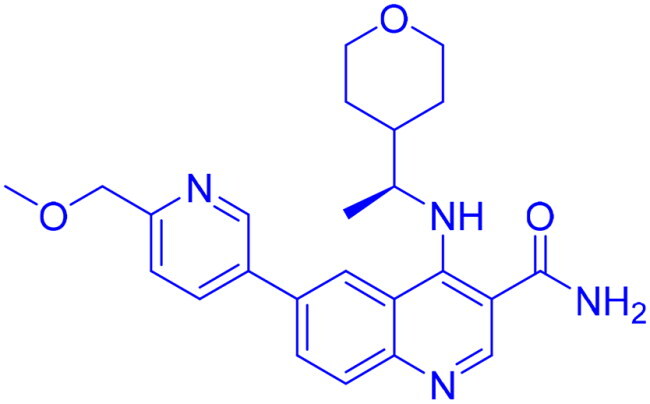	ATM ATR	Colorectal cancer, triple negative breast cancer	**AZ31 (5)** combined with irinotecan enhances antitumor effects in colorectal cancer models, while its synergy with the PARP inhibitor olaparib shows increased cytotoxicity in triple-negative breast cancer and HeLa cells.	^106^ ^,^ ^107^
**AZD0156 (6)** 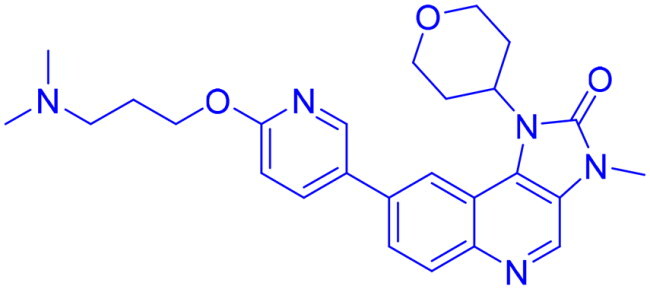	ATM ATR	Colorectal cancer, neuroblastoma, lymphoma, triple negative breast cancer	**AZD0156 (6)** monotherapy or sequential treatment with olaparib in TNBC reduced pRAD50 by 34–72%, while post-irinotecan ATM inhibition decreased pRAD50 by 68% in a colorectal cancer xenograft. Combining **AZD0156 (6)** with temozolomide and irinotecan enhanced antitumor efficacy *in vitro* and neuroblastoma xenograft models. Combination of AZD0156 and Olaparib has been reported for minor toxicities in Lymphoma	^108–110^
**AZD1390 (7)** 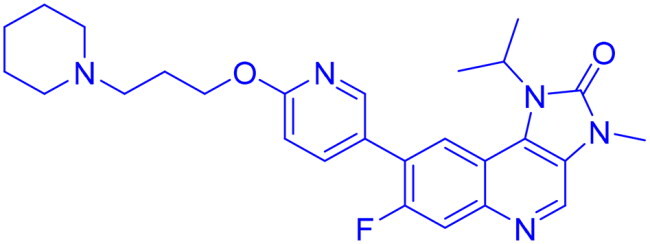	ATM, ATR, PI3K, mTOR, DNA-PK	Glioblastoma, sarcoma and NSCLC, breast cancer	**AZD1390 (7)** inhibits ATM-dependent DNA repair, enhances radiotherapy efficacy by inhibiting the repair of radiation-induced damage, and effectively penetrates the blood-brain barrier to target glioblastoma tumours, NSCLC and soft tissue sarcoma directly. Elevate anti-tumour effects in breast cancer cell lines by combination with cisplatin	^111^ ^,^ ^112^
**AZ32 (8)** 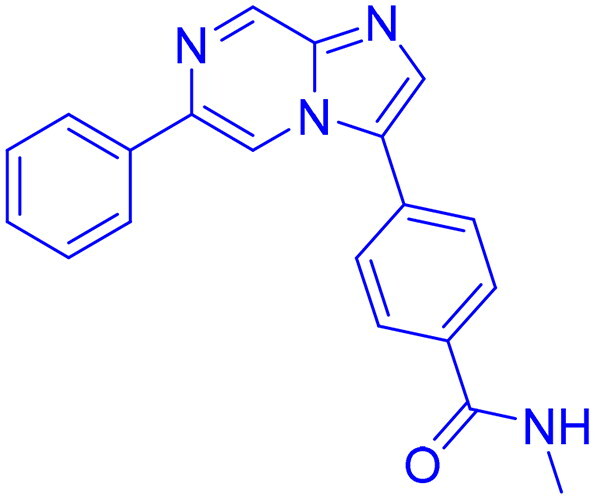	ATM	Glioblastoma, colorectal cancer	**AZ32 (10)** enhances radiotherapy sensitisation in mutant p53 gliomas and metastatic brain tumours while also reversing multidrug resistance to chemotherapeutics in colorectal cancer.	^113–115^
**CP466722 (9)** 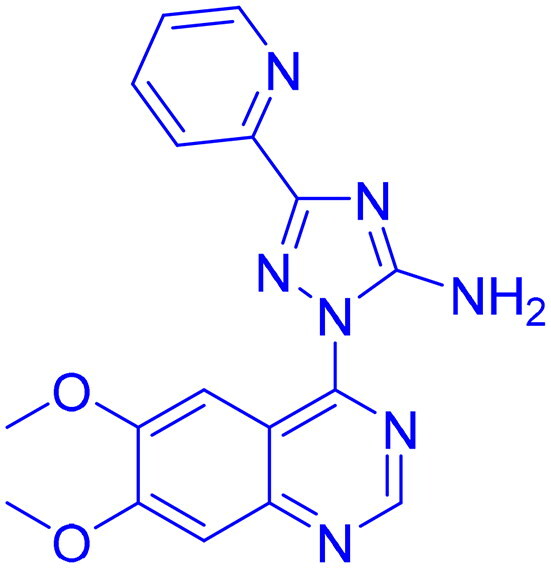	ATM	NSCLC	CP466722 **(9)** suppresses epithelial-mesenchymal transition and downregulates PD-L1 expression	^116^
**M3541 (10)** 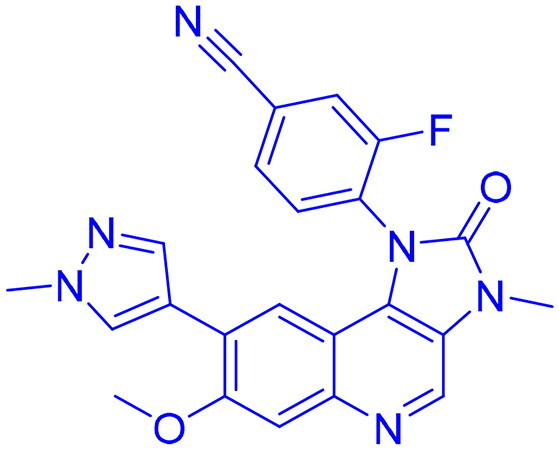	ATM, CHK2, KAP1 and p53	Lung cancer, colorectal cancer	M3541 **(10)** enhances radiotherapy efficacy in preclinical Lung & colorectal cancer models and targets FA/BRCA-deficient tumours via synthetic lethality.	^117^
**M4076 (11)** 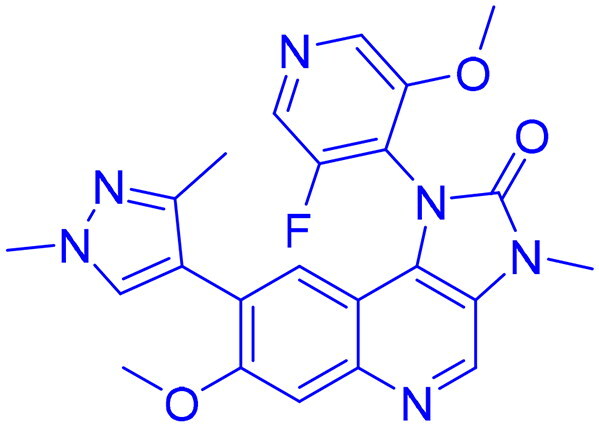 **WSD 0628** 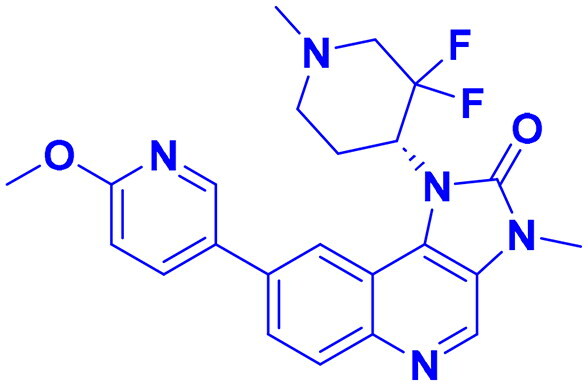	ATM, DNA-PK ATR ATM	Breast cancer, lung cancerBrain Cancer	Oral M4076 enhanced antitumor efficacy in mice through colony formation suppression in diverse cancer cell lines. Suppressed autophosphorylation of ATM at serine 1981 and ATM-mediated phosphorylation of Chk2 at threonine-68 and KAP1 at serine-824	^118^ ^,^ ^119^ ^,^ ^120^

In summary, ATM inhibitors represent a promising therapeutic approach by targeting the DNA damage response in cancer cells, thereby compromising their ability to repair DNA and enhancing susceptibility to cell death. Initial inhibitors, such as Wortmannin, exhibited broad-spectrum activity but were hampered by significant off-target effects, limiting their clinical utility. This prompted the development of more refined and selective compounds, including KU-55933 and KU-60019, which have shown notable efficacy in preclinical models of breast, prostate, and lung cancers. Other inhibitors, like KU-59403, specifically enhance the effects of topoisomerase poisons in colon cancer, while AZ31 and AZD0156, dual ATM/ATR inhibitors, exhibit synergy with chemotherapy in colorectal and triple-negative breast cancer. Advanced inhibitors such as AZD1390 and AZ32 cross the blood-brain barrier, showing potential for glioblastoma and metastatic cancers. CP466722 has been effective in NSCLC by targeting immune evasion, whereas multi-target inhibitors like M3541 and M4076 enhance radiotherapy responses in lung, colorectal, and breast cancers. Recent trends emphasise improved selectivity, with inhibitors like AZD0156 reducing off-target toxicity. Combination therapies, particularly with DNA-damaging agents like irinotecan, temozolomide, and PARP inhibitors, have shown enhanced therapeutic effects. Additionally, some ATM inhibitors, including AZD1390 and M3541, have been effective in radio sensitisation, increasing their potential in glioblastoma and NSCLC treatment. However, challenges such as drug resistance, toxicity, and better pharmacokinetics, requiring further refinement through structure-based drug design and optimised combination strategies.

## Ataxia Telangiectasia and Rad3-related (ATR)

### Structural insights and mechanism of ATR kinase activation

**Figure 3.**
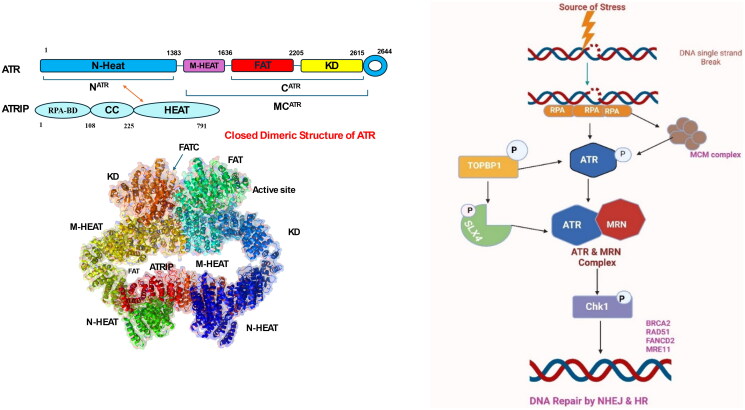
(a) Dimeric structure of ATR protein and (b) activation of ATR for DNA single strand damage (the figure was drawn by the authors using BioRender software).

ATR (Ataxia Telangiectasia and Rad3-related) is a protein from the serine/threonine kinase family that plays a vital role in DNA damage response (DDR), especially responding to replication stress and single-stranded DNA regions. Structurally, ATR consists of several domains that contribute to its function: the N-terminal HEAT repeats facilitate protein-protein interactions, enabling ATR to bind its associated protein ATRIP (ATR-Interacting Protein) and other regulatory molecules; the FAT domain provides structural stability and regulatory support; the kinase domain at the C-terminal end is catalytically active and phosphorylates downstream targets; and the FATC domain at the extreme C-terminus is essential for maintaining kinase integrity (Figure 3(a)). ATR is directed to DNA damage sites through its partnership with ATRIP, which recognises and attach to single-stranded DNA coated with replication protein A (RPA)^121^^,^^122^. The mechanism of ATR activation has been progressively unravelled over the past decade, with emerging research revealing that its activation and checkpoint functions are regulated by a complex signalling network, which includes chromatin-restructuring proteins and DNA replication sensors^123^^,^^124^. ATR activation at the molecular stage is primarily initiated through the existence of single-stranded DNA. During DNA replication, the unwinding of double-stranded DNA (dsDNA) exposes ssDNA, which serves as a crucial signal for ATR activation. COPI, A coatomer proteins first interact with double-strand DNA and binds to the proliferating cell nuclear antigen (PCNA) sliding clamp, facilitating the action of translocases such as WRNIP1, FBH1, and ZRANB3 to promote ssDNA exposure^125^. RPA then binds to the newly formed ssDNA, facilitating the recruitment and activation of ATR. This process is further regulated by the MCM helicase complex, whose activation is mediated by PICH, a protein that binds to UBC9 to maintain the SUMOylation levels of MCM. The TOPBP1-AAD domain undergoes autophosphorylation, promoting ubiquitination of the ATR-interacting BEACH domain of SLX4 by UBR5, which facilitates the ATR-mediated phosphorylation of ID2, triggering the ATR-MRN-ATRX signalling cascade. Upon activation of the repair mechanism, ATR phosphorylates checkpoint kinase 1 (CHK1), halting the cell cycle, initiating the non-homologous end joining (NHEJ) repair process via phosphorylation of MRE11, and activating the Fanconi anaemia (FA) pathway, thereby promoting homologous recombination (HR) repair of DNA damage (Figure 3(b))^126–128^.

### Role of ATR and ATR inhibitor in cancer

ATR (Ataxia Telangiectasia and Rad3-related protein) is essential for preserving genomic stability by involving in DNA repair pathways. Elevated ATR level has been identified as a significant biomarker in several types of cancer, highlighting its potential as a therapeutic target. This has spurred extensive research into the mechanistic link between ATR activation and homologous recombination (HR), with a focus on ATR-mediated phosphorylation events and the functional significance of its substrates. Despite these advances, the structural complexity of ATR continues to challenge a comprehensive understanding of its role in tumorigenesis. Nevertheless, abundant evidence underscore the impact of ATR overexpression on cancer progression^129–131^. For instance, in mouse models of pancreatic tumours, the co-expression of mutated *KRAS* and catalytically active ATR mutations accelerate tumorigenesis and suppress immune responses. In contrast, ATR knockout or inhibition results in significant tumour growth suppression^132^. ATR is particularly activated in many tumours in response to replication stress, often triggered by proto-oncogenes, for instance, *Ras*, *Myc*, and *Cyclin E*, leading to dysregulated cell cycle progression and increased cellular proliferation^133^. Moreover, the loss of DNA repair proteins, such as XRCC1 and ERCC1, enhances the vulnerability of cancer cells to ATR inhibitors, as demonstrated in preclinical models^134^. Previous research also shows that tumours in hypoxic environments, where limited oxygen availability hinders DNA replication and repair, are susceptible to ATR inhibition. In these conditions, ATR is essential for controlling the replication stress, indicating that its inhibition is a promising strategy for targeting hypoxic tumour cells^135^^,^^136^. Additionally, ATR also functions as a crucial safeguard in maintaining telomere integrity in tumours that alter the alternative lengthening of the telomeres (ALT) pathway. Supporting this role, studies have shown that ATR inhibition disrupts homologous recombination mechanisms necessary for telomere maintenance in ALT-positive tumours, leading to genomic instability and eventual cell death^137–139^. Studies have further highlighted that ATR inhibitors are particularly effective in tumour cells with defective p53 pathways. Since these cells have compromised G1/S checkpoint control and increased replication stress, they become more reliant on ATR. Without p53-mediated regulation, cells are unable to control progression into S-phase, making them more dependent on ATR for managing replication-associated stress. This selective sensitivity of p53-deficient tumours to ATR inhibitors suggests a promising therapeutic approach with the prospective for fewer unwanted effects in normal tissues^140^. These findings have driven efforts to introduce highly potent and selective ATR inhibitors for cancer therapy (Table 2). In this context, NU6027 **(12)** was recognised as a formidable ATR inhibitor, though it was initially exploited as a CDK2 inhibitor in 2011. NU6027 **(12)** has shown the ability to alert breast and ovarian cancer cell lines to ionising radiation (IR) and various antineoplastic agents despite its lack of selectivity for ATR^141^^,^^142^. Also in 2011, Toledo and colleagues identified dactolisib **(14)** through a cell-based compound library screen. While initially exploited as a dual PI3K and mTOR inhibitor^165^, Dactolisib **(14)** was found to also inhibit ATR, contributing to its ability to radio-sensitise Ras-overexpressing tumours. Several other compounds with ATR inhibitory activity, such as ETP-46464 **(15)** and Torin 2 **(17),** have also been identified, but like their predecessors, they lack selectivity^166–168^. This guided the finding of the first extremely potent and selective ATR inhibitors by Vertex Pharmaceuticals, VE-821 **(18),** which demonstrated significant ATR inhibition while sparing related kinases like ATM, DNA-PK, and mTOR. VE-821 **(18)** exhibited synergistic activity with genotoxic agents, particularly in cancer cells lacking p53 or ATM function. In normal cells, it demonstrated low toxicity, inducing only reversible cell cycle arrest without causing permanent damage^169–175^. Following these findings, VE-821 **(18),** was further developed into VE-822 **(19)** (later known as VX-970), which has improved pharmacokinetic properties and has been shown to heighten the results of radiation and chemotherapy therapy, especially in hypoxic tumour cells. VE-821 **(18)** entered clinical trials and demonstrated the ability to enhance the efficiency of DNA-damaging therapies, particularly in p53-deficient cancer cells, while sparing normal tissues^176–178^. Meanwhile, AZD6738 **(21),** developed by AstraZeneca as an analogue of AZ20 **(20),** has shown significant potential as an orally bioavailable ATR inhibitor. AZD6738 **(21),** demonstrated strong anti-tumour activity, particularly in ATM-deficient models, and is presently being assessed in a phase I clinical trial. Its ability to increase DNA damage markers like γH2AX in tumour tissue without causing lasting damage to normal tissues suggests a favourable therapeutic index for further clinical development^179–183^. ATR inhibition with AZD6738 **(21),** enhanced the effects of radiotherapy, particularly in PTEN-deficient models of non-small cell lung cancer (aggressive type of lung cancer). In multiple myeloma (MM), ATR inhibition combined with MEK1/2 inhibitors, such as AZD6738 **(21),** triggered dual STAT3 dephosphorylation, leading to downregulation of survival proteins (c-Myc, BCL-XL) and increased MM cell death. Additionally, the combination of S-1 and AZD6738 **(21),** showed a synergistic effect in suppressing tumour cell proliferation and inducing apoptosis, significantly reducing tumour volume *in vivo*^184^^,^^185^. Yves Pommier’s team developed the ATR inhibitor Gartisertib, later known as M4344 **(16),** which showed superior potency compared to VE-822 **(19)**, and AZD6738 **(21)**. M4344 **(16)** was evaluated for its anticancer efficacy both as a standalone treatment and in combination with DNA-damaging drugs across cancer cell lines, patient-derived tumour organoids, and mouse models. The compound demonstrated significant effectiveness, particularly in tumours characterised by replication stress and neuroendocrine gene expression. M4344 **(16)** promotes cell death by inducing DNA damage and exhibits synergistic effects with chemotherapeutic agents such as topotecan and irinotecan, thereby enhancing anticancer activity^147^. In another study, M4344 **(16)** has been shown to abrogate ATM-dependent cell cycle checkpoints and impair double-strand break (DSB) repair mechanisms, thereby attenuating the p53-mediated genomic surveillance pathway and prolonging the persistence of ATR inhibitor-induced DSBs^186^. In glioblastoma, ATR inhibition using M4344 **(16)** sensitised cells to temozolomide and radiation therapy. In hepatocellular carcinoma (HCC), TuBG1 overexpression was linked to poor prognosis, and its silencing led to increased ATR, p-P53, and apoptosis-related protein expression, reducing proliferation by M4344 **(16).** In ovarian cancer, SLFN11 downregulation contributed to cisplatin resistance, which was reversible by HDAC inhibition. BAY 1895344 exhibited strong anticancer activity across multiple cancer types. In anaplastic thyroid cancer (ATC), it induced dose-response cytotoxicity, caused S-phase and G2-phase arrest, activated caspase-3, and promoted apoptosis. It significantly suppressed tumour growth in xenograft models and showed synergistic effects with dabrafenib + trametinib and lenvatinib. In HPV-negative head and neck squamous cell carcinoma (HNSCC), BAY 1895344 enhanced radiosensitivity by disrupting the G2/M checkpoint, leading to mitotic catastrophe and improved tumour growth suppression in vivo. In triple-negative breast cancer (TNBC), ATR inhibition combined with LBH depletion increased DNA replication stress and cell death. In lymphoma, BAY 1895344 exhibited potent activity and was effective in combination with the PI3K inhibitor copanlisib^162^^,^^187–193^. RP-3500 **(13),** a next-generation oral ATR kinase inhibitor developed by Anne Roulston, is currently under clinical investigation and demonstrates exceptional potency and remarkable selectivity towards ATR, with a 30-fold higher specificity relative to mTOR and over 2,000-fold selectivity compared to other kinases, including ATM, DNA-PK, and PI3Kα^194^. Preclinical *in vivo* studies revealed that RP-3500 **(13)** induces significant tumour regression across various xenograft models^195^. Furthermore, Nishida and team identified *Schisandrin B*, dibenzocyclooctadiene lignan derived from *Schisandra chinensis*, as a selective ATR inhibitor. *Schisandrin B* was observed to disrupt cell cycle checkpoints, thereby potentiating the cytotoxicity of UV radiation in lung carcinoma cells. However, its ATR repressive efficacy is relatively modest, requiring elevated concentrations in cellular assays to exert significant biological effects^196^. ATR inhibitors demonstrate therapeutic limitations as monotherapies, necessitating combinatorial approaches with PARP inhibitors. Investigations utilising cholangiocarcinoma (CCA) cell lines indicate that an elevated burden of DNA damage response (DDR) mutations correlates with heightened sensitivity to both ATR inhibition (AZD6738) and PARP inhibition (olaparib, veliparib, talazoparib), with talazoparib emerging as the most potent agent. The combined administration of AZD6738 and talazoparib synergistically exacerbates DNA damage, particularly in cell lines exhibiting lower intrinsic responsiveness. In cervical cancer models, therapeutic synergy is observed exclusively when ATR inhibition precedes PARP inhibition, as ATR suppression upregulates PARP expression. This combinatorial regimen induces G2-phase cell cycle arrest, apoptosis, and extensive DNA damage; however, ATR inhibition alone significantly impairs xenograft tumour growth, with no additional therapeutic advantage conferred by PARP inhibition. Furthermore, a pioneering study highlights the development of novel inhibitors integrating olaparib and AZ20 pharmacophores, which exhibit 6- to 14-fold greater efficacy compared to the olaparib–AZD6738 combination. These hybrid inhibitors demonstrate robust anti-proliferative activity in both BRCA wild-type and mutant triple-negative breast cancer (TNBC) cell lines^197–199^. Collectively, these outcomes emphasise the therapeutic prospective of ATR inhibition as a targeted approach for oncological interventions. Cancer cells experiencing elevated replication stress or harbouring deficiencies in significant DNA repair pathways exhibit heightened susceptibility to ATR inhibitors, thereby enhancing the efficacy of genotoxic agents and promoting tumour cell eradication.

**Table 2.** Different class of ATR inhibitors used for cancer treatment.

Compound name	Target	Cancer type	Description	References
**NU6027 (12)** 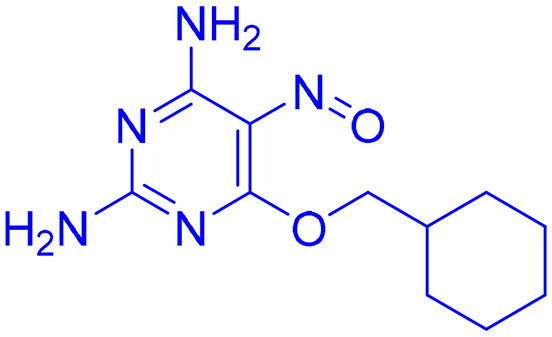	ATR ATP CDK1 CDK2	Ovarian cancer, breast cancer	Demonstrates potent inhibition of ATR activity in GM847KD cells, with an IC_50_ value of 2.8 μM Elevate double-strand DNA breaks and impairs the G2/M checkpoint reaction to DNA damage in MCF7 cells.	^141^ ^,^ ^142^
**RP3500 (13)** 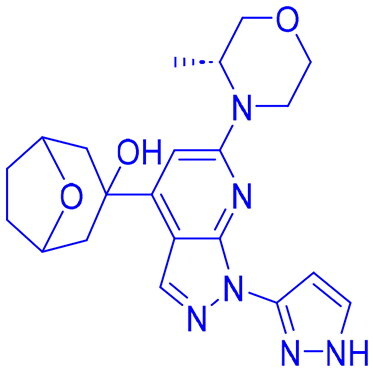	ATR ATM mTOR PI3Kα	Colorectal cancer, advanced solid tumour	Highly selectivity for ATR inhibition Combination with PARP inhibitor for ALT+ cancer	^143^
**Dactolisib (14)** NVPBE2235 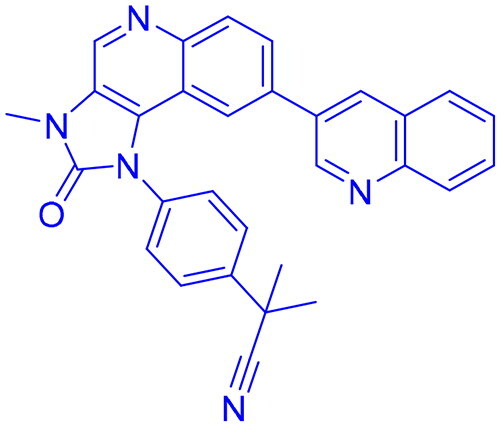	PI3K mTOR ATR ATM DNA-PK	Breast cancer, glioblastoma, advanced solid tumour	Decreased the level of vascular endothelial growth factor in Gliomas Prevent the initiation of the downstream effects of Akt, S6 ribosomal protein, and 4EBP1 in breast cancer cells.	^144^ ^,^ ^145^
**ETP-46464 (15)** 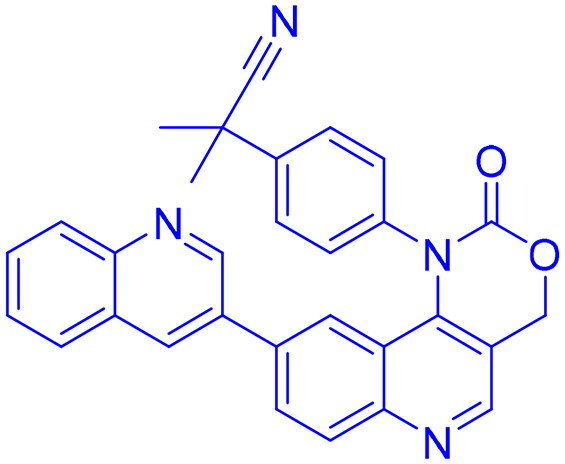	ATR mTOR	Ovarian cancer, endometrial and cervical cancer	Generate replicative stress, leading to Chromosomal Breakage in tumour Anticancer effects in Gynaecologic Cancer cell lines	^146^
**Gartisertib (M4344) (16)** 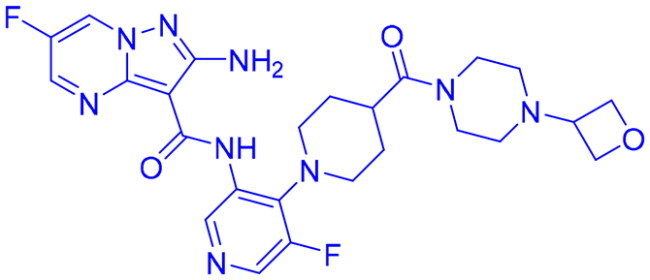	ATR Chk1	Acute lymphocytic leukaemia	Showing synergistic anticancer effects with drugs like topotecan and irinotecan	^147^
**Torin 2 (17)** 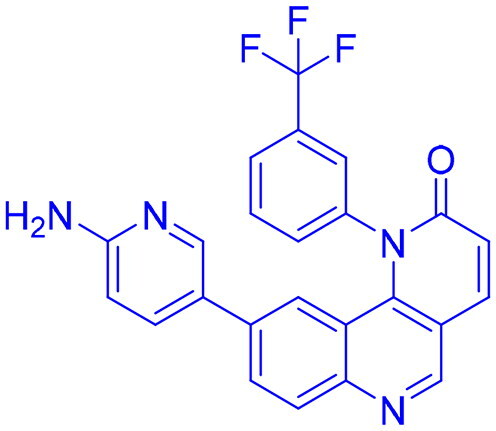	ATR ATM mTOR mTORC1 mTORC2	Liver cancer, glioblastoma	Induce autophagy and downregulation of UHRF1 expression Suppress mTORC1 and mTORC2 in glioblastoma	^148^ ^,^ ^149^
**VE-821 (18)** 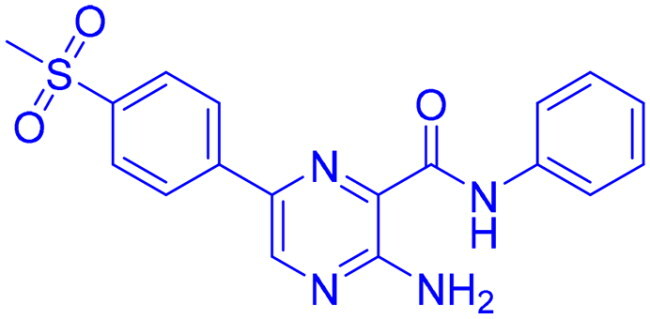	ATR CHk1	Sarcoma, pancreatic cancer, lymphoblastic, leukaemia cells	Combination of VE821 with other anticancer drugs led to strong apoptosis Reduce PD-L1 expression & CD44 expression VE-821 and doxorubicin-tempted synergistic effects in cancer cell lines	^150–152^
**Berzosertib** (VE-822) **(19)** 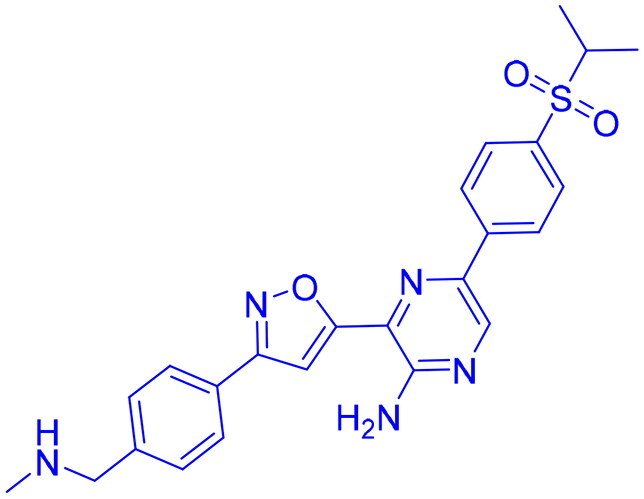	ATR ATM PI3Kγ	Colorectal cancer, NSCLC, HNSCC	Incorporation of VE-822 into radiotherapy protocols significantly enhanced STING pathway activation in colorectal cancer mouse models Combination of Berzosertib and Topotecan elevates overall survival of Lung Cancer Patients VE-822 boosted the anticancer effect in cisplatin-resistant HNSCC VE-822 effectively reverses 5-fluorouracil (5-FU) resistance in colorectal cancer cell lines	^153–155^
**AZ20 (20)** 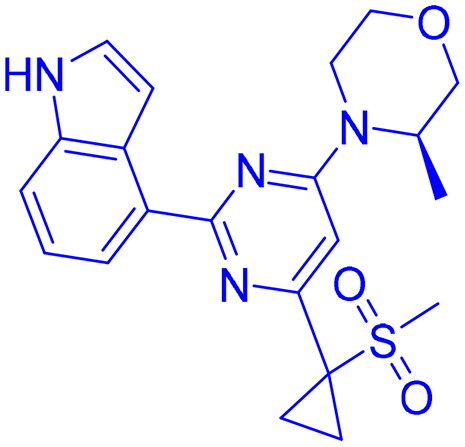	ATR mTOR PI3Kα	Ovarian cancer, breast cancer, liver cancer, lung cancer, brain cancer	AZ20 and PARP inhibitors showing synergistic anticancer effects in combination therapy	^156^
**AZD6738 (21)** 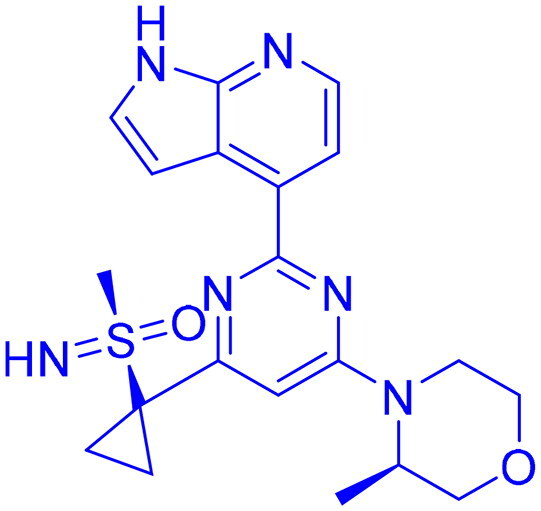	ATR PI3Kδ DYRK	Metastatic melanoma, advanced gastric cancer, colorectal cancer	Combination of Ceralasertib and Durvalumab showing synergistic effects metastatic melanoma and gastric cancer AZD6738 amplified the effects of 5-FU on p53-mutated colorectal cancer	^157–159^
**Tuvusertib (22)** 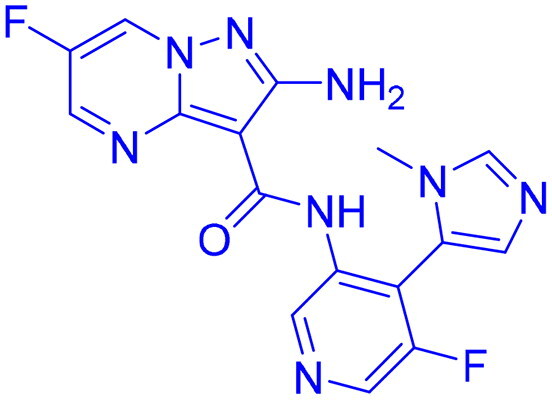	ATR	Solid tumour	Prevents the phosphorylation and activation of downstream effectors, such as CHK1 and RAD17. Efficiently blocked the activation of the ATRCHK1 checkpoint pathway caused by replication stress induced by TOP1 inhibitors	^160^
**ART0380 (23)** 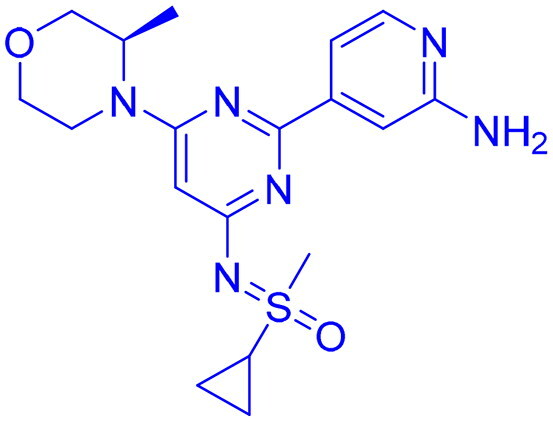	ATR	Advanced or metastatic solid tumours	Showing potent anticancer activity in LoVo xenograft model and excellent pharmacokinetics profile	^161^
**BAY 1895344 (24)**	ATR	Head and neck squamous cell carcinoma, anaplastic thyroid cancer, triple negative breast cancer	The combination of BAY 1816032 and paclitaxel exhibited a pronounced suppression of tumour cell proliferation, a substantial enhancement in radiotherapy efficacy, and a notable decrease in tumour volume, all while maintaining an outstanding safety and tolerability profile.	^162–164^

Overall, ATR inhibitors are transforming cancer therapy by targeting DNA repair vulnerabilities and boosting the effectiveness of genotoxic treatments. Early inhibitors like NU6027 and Torin 2 lacked selectivity, affecting other kinases, but advancements led to more precise compounds such as VE-821 and VE-822, which enhance the efficacy of DNA-damaging treatments. Next-generation inhibitors like AZD6738, M4344, and RP-3500 offer improved potency and specificity, showing promise in clinical trials. Natural compounds like Schisandrin B also inhibit ATR but require higher concentrations.

## DNA-dependent protein kinase catalytic subunit (DNA-PKcs)

### Structural insights and mechanism of DNA-PK activation

**Figure 4.**
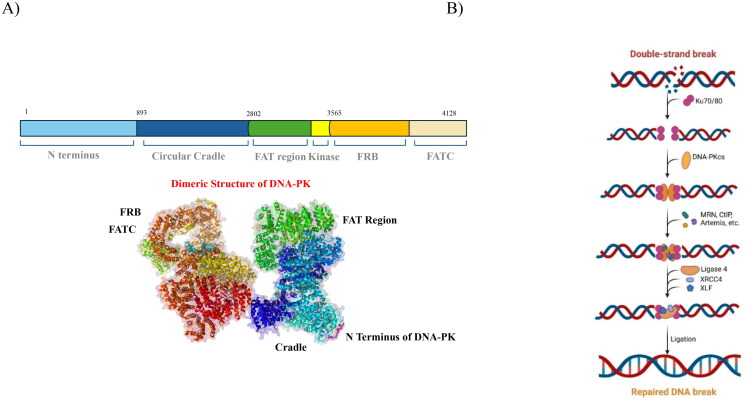
(a) Dimeric structure of DNA-PK and (b) DNA repair mechanism of DNA-PK (the figure was drawn by the authors using BioRender software).

DNA-PK (DNA-dependent protein kinase) is a key enzyme within the non-homologous end-joining (NHEJ) pathway, playing an essential role in facilitating the repair of DNA double-strand breaks. It consists of two primary components: the DNA-PK catalytic subunit (DNA-PKcs), a large serine/threonine kinase with a molecular weight of approximately 460 kDa, and the Ku heterodimer (Ku70/Ku80), which recognises and binds to DNA termini. Structural elucidation through cryo-electron microscopy (PDB: 5LUQ) (Figure 4(a)) has provided comprehensive insights into the architecture of DNA-PK. DNA-PKcs comprises several key domains, including N-terminal HEAT repeats, the FAT domain (FRAP-ATM-TRRAP), the kinase domain, and the FATC domain at the C-terminus. These domains collectively form a structural framework that stabilises the protein complex and modulates its kinase activity during the DNA repair process^200^^,^^201^.

The Ku complex is rapidly drawn to DSB sites, where it attaches itself to DNA ends and causes DNA-PK to undergo conformational changes, so initiating its catalytic activity^202–206^. DNA-PK is known to phosphorylate various proteins involved in the NHEJ process, including Ku, XRCC4, and XLF, but its most critical and well-characterised substrate is itself. Autophosphorylation of DNA-PK alters its conformation, facilitating the accessibility of DNA ends to processing enzymes such as Artemis nuclease and Pol X family polymerases, while also promoting the release of DNA-PK from the DNA ends. Interestingly, although autophosphorylation enhances the accessibility of the DNA ends, it also restricts the extent of DNA end processing, thus regulating the process (Figure 4(b))^207^^,^^208^.

The loss of DNA-PK leads to profound defects in DNA repair mechanisms, manifesting as severe combined immunodeficiency (SCID) *in vivo* due to its significant role in double-strand break (DSB) repair through the non-homologous end joining (NHEJ) pathway, particularly in processes such as V(D)J recombination and class switch recombination. Beyond its established function in DNA repair, DNA-PK have been implicated in several nuclear processes^209^^,^^210^. For instance, it plays a pivotal role in telomere maintenance by phosphorylating hnRNPA1, a key factor involved in telomere capping and stability. DNA-PK has also been presented as regulating transcription through the phosphorylation of various transcription factors, including SP1, one of its earliest identified substrates, which facilitates the assembly of transcriptional complexes at gene promoters^211^^,^^212^. Additionally, the kinase modulates RNA polymerase II-driven transcription by phosphorylating the co-regulatory protein TRIM28, a process essential for efficient gene expression. Inhibition or depletion of DNA-PK has been associated with reduced transcriptional activity, highlighting its importance in RNA synthesis. Moreover, DNA-PKcs contribute to ribosome biosynthesis by interacting with small nucleolar RNAs (snoRNAs) within the U3 processome of the ribosomal subunit. This interaction activates DNA-PK, leading to its autophosphorylation. Mutations that disrupt its catalytic activity or hinder autophosphorylation impair rRNA processing, causing deficiencies in ribosome assembly and subsequent translational defects, particularly in haematopoietic cells^213^. Literature precedents claim that DNA-PK has a key role in maintaining replication stress by promoting replication fork reversal and decelerating fork progression to prevent excessive damage during DNA synthesis. However, the precise substrates and molecular mechanisms by which DNA-PK regulates NHEJ, RNA processing, and replication stress responses remain incompletely understood. A significant challenge in elucidating these pathways lies in the incomplete characterisation of DNA-PK signalling networks and the overlapping functional roles shared with ATM kinase, which further complicates the identification of specific DNA-PK targets and downstream effectors^214^.

### Role of DNA-PK in cancer development

DNA-PK deficiency or mutation has significant implications for human diseases and cancer, primarily due to its prime role in DNA repair mechanisms. This deficiency is linked to various pathologies, including immunodeficiencies, neurological disorders, and increased cancer susceptibility^215^. DNA-PK deficiency causes genomic instability by misrecognising uncapped telomeres as double-strand breaks, leading to improper repair and telomere fusion, accelerating cancer progression, especially under ionising radiation. Additionally, genetic variations reducing DNA-PK activity increase radiation-induced breast cancer susceptibility, and reluctance to PARP inhibitors in BRCA1-mutated tumour cells is mediated by DNA-PK activation, which enhances homologous recombination repair, contributing to chemotherapy resistance^216–218^. Earlier investigations show that depletion of DNA-PK significantly impairs migration, invasion, and proliferation of HMEC-1 cells in glioma tumours. These inhibitory effects are potentially associated with a reduction in VEGF secretion and downregulation of HIF-1α expression levels. Furthermore, HepG2 cells exposed to cisplatin and fluorouracil (5-Fu), the depletion of DNA-PK guided to an elaboration of pro-apoptotic proteins, including p53 and caspase-3, while concurrently reducing Akt phosphorylation and Bcl-2 expression. Notably, 5-Fu alone appears to modestly enhance NF-κB activity, whereas cisplatin has been shown to downregulate NF-κB transcriptional activity through the degradation of IκB-α. Silencing DNA-PK via siRNA further amplified the marked reduction in NF-κB transcriptional activity observed with the combined cisplatin and 5-Fu treatment. Mounting evidence from numerous studies highlights the central role of DNA-PK in the pathogenesis and advancement of hepatocellular carcinoma^219^^,^^220^. Extensive research on hepatocellular carcinoma (HCC) cell lines has demonstrated that elevated DNA-PK levels are closely associated with increased expression of key DNA repair proteins, particularly XRCC6 and XRCC5. In contrast, other DNA repair-related genes, such as ATM, XRCC6, LIG4, and XRCC4, exhibit no notable overexpression within these cells. These genes play important roles in various DNA repair pathways, including mismatch repair (MMR), nucleotide excision repair (NER), base excision repair (BER), and homologous recombination repair (HRR), all of which are essential for preserving genomic stability and mitigating tumour development^221^. Recent findings suggest that DNA-PK undergo post-transcriptional regulation in hepatocellular carcinoma (HCC). Notably, the ATPase family protein Reptin (RUVBL2), identified as a potential liver oncogene, has been shown to directly interact with HCC cells, thereby stabilising DNA-PK protein levels. Importantly, this study also demonstrated that Reptin (RUVBL2) plays a similar role in stabilising ATM kinase, highlighting its key function in facilitating the repair of double-strand breaks (DSBs) in HCC cells and promoting genomic stability in tumour progression^222^. Recent studies indicate that tankyrase 1 binding protein 1 (TNKS1BP1), a member of the poly(ADP-ribose) polymerase (PARP) superfamily, is a prime mediator of DNA repair processes. TNKS1BP1 was shown to be physically associated with both PARP-1 and DNA-PK, facilitating their interaction across various cell lines, including HepG2. Notably, TNKS1BP1 overexpression promotes the autophosphorylation of DNA-PK at Ser2056 in a PARP-1-dependent manner, thereby enhancing the activity of the DNA double-strand break (DSB) repair pathway. Additionally, amplification of the MYC gene family is frequently observed in small-cell lung cancer (SCLC), resulting in elevated expression of the c-MYC oncoprotein, which drives cellular survival, proliferation, and tumour progression^223^. Other side OCT4, a transcription factor, has been shown to drive the activation of MYC. A study by Sung Jen Wei et al. identified a novel signalling pathway in which OCT4 interacts with DNA-PK to regulate c-MYC expression and facilitate DNA damage repair. Their findings demonstrate that blockage of DNA-PK or disruption of the interaction between OCT4 and DNA-PK provokes a reduction in c-MYC expression and exerts anticancer effects in both SCLC cell lines and xenograft models^224^.

Targeting DNA-PK in cancer treatment represents a promising therapeutic strategy as shown in Table 3. Early advancements in DNA-PK inhibition utilised quercetin **(25)**^225^, a non-selective PI3K inhibitor, and LY294002 **(26)**^226^ as foundational compounds for developing more potent and selective inhibitors. Notably, NU7026 **(27)** and NU7441 **(28)** emerged as highly potent and selective DNA-PK inhibitors with minimal off-target effects on related kinases such as ATM and ATR. Among these, NU7441 **(28)** demonstrated significant potential by enhancing the ability of DNA-damaging chemotherapy and radiotherapy in multifarious cancer cell lines, including breast, lung, colorectal, and nasopharyngeal malignancies. However, despite their *in vitro* effectiveness and selectivity, both NU7026 **(27)** and NU7441 **(28)** were hindered by suboptimal pharmacokinetics and limited oral bioavailability^227–230^. Another breakthrough was the development of KU-0060648 **(29)**, a dual DNA-PK and PI3K inhibitor with better solubility and pharmacokinetics than NU7441 **(28)**^231^. Recent progress in structural elucidation has driven the development of highly potent and selective inhibitors, exemplified by peposertib (M3814), which has demonstrated substantial efficacy in increasing the susceptibility of cancer cells to both radiation therapy and chemotherapeutic agents. Findings from Phase I and II clinical trials indicate that M3814 is well-tolerated, producing mild adverse effects, while demonstrating efficacy in combination treatments for advanced solid tumours and leukaemia. Similarly, CC-115 **(31),** a dual DNA-PK and mTOR inhibitor, has shown potential in certain cancers, achieving stable disease in CRPC and glioblastoma patients, though it was discontinued in glioblastoma trials due to toxicity and limited efficacy^232–239^. AZD7648 **(32)** and XRD-0394 **(33)** are newer inhibitors showing encouraging preclinical results, with ongoing clinical evaluations for metastatic and advanced tumours^240–242^. Numerous preclinical studies underscore the critical role of DNA-dependent protein kinase (DNA-PK) inhibitors in combination with other anticancer agents, particularly in the context of synthetic lethality-based therapeutic strategies. These approaches have been extensively investigated in conjunction with poly(ADP-ribose) polymerase inhibitors (PARPi), such as olaparib, especially in BRCA1/2-deficient malignancies, including ovarian cancer. In such scenarios, the downregulation of vaccinia-related kinase-1 (VRK1) exacerbates DNA-PK instability, thereby augmenting the therapeutic efficacy of PARPi. Furthermore, DNA-PK inhibitors are being actively explored as radiosensitizing agents in various cancer models. For instance, AZD7648 **(32)** has demonstrated superior radiosensitizing potency compared to olaparib, particularly in BRCA2-deficient contexts. However, its clinical utility is constrained by heightened toxicity profiles in non-malignant tissues. Similarly, the combination of Peposertib with standard-of-care (SOC) chemoradiation has yielded enhanced clinical responses in rectal cancer, albeit with heterogeneous pathological outcomes. This variability suggests the potential activation of compensatory DNA repair mechanisms, which may mitigate the therapeutic impact and warrant further mechanistic investigation^91^^,^^243–247^.

**Table 3.** Different DNA-PK and their role in cancer treatments.

Compound name	Target	Cancer type	Description	References
**Quercetin (25)** 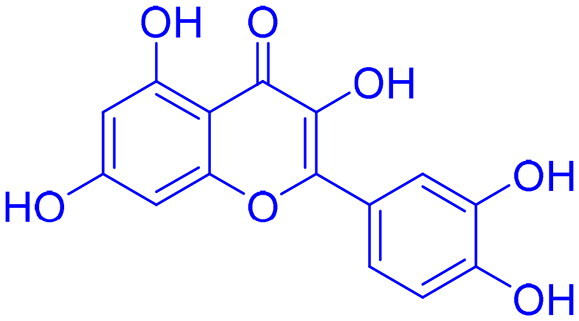	PI3K DNA-PK	NSCLC	Inhibit the phosphorylation of ATR and ATM in the HR pathway and DNA-PK and Ku70 in the NHEJ pathway.	^225^
**LY294002 (26)** 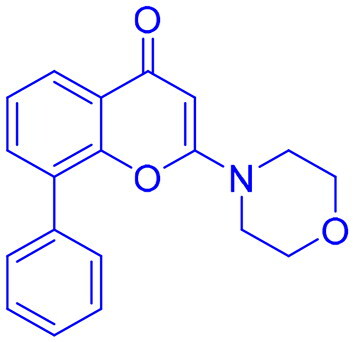	DNA-PK	Glioblastoma, pancreatic cancer	LY294002 showed promising results as a radio- and chemo-sensitising agent in various tumour models in vitro and in vivo. However, its lack of specificity, rapid metabolism, poor stability, and unfavourable toxicity made it unsuitable for clinical use.	^226^
**NU7026 (27)** 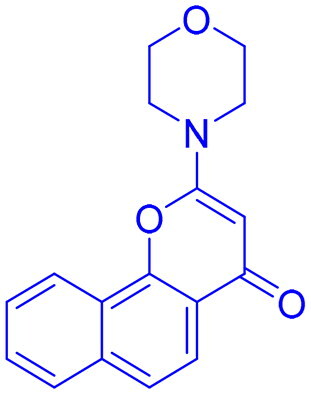	DNA-PK	Gastric cancer, breast cancer	NU7026 enhances irradiation and etoposide-produce DNA destruction, causing G2/M arrest and cell death, and synergizes with ß-lapachone to selectively target NQO1-positive cancer cells.	^227^ ^,^ ^228^
**NU7441 (28)** 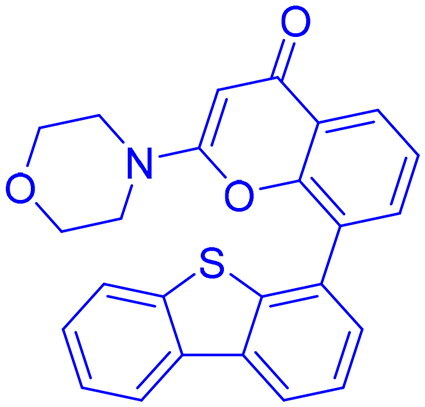	DNA-PK	Breast cancer	NU7441 alerted cancer cells to radiation and doxorubicin, with the strongest effect in MDA-MB-231 cells, enhancing G2/M arrest and delaying DNA repair via DNA-PK inhibition.	^229^ ^,^ ^230^
**KU-0060648 (29)** 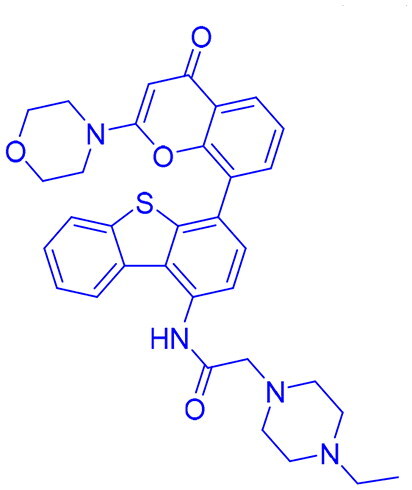	DNA-PK AKT mTOR	Breast cancer, colon cancer, hepatocellular carcinoma	KU-0060648 enhanced etoposide-induced tumour delay by up to 4.5-fold in SW620 and MCF7 xenografts, inhibited PI3K-AKT-mTOR signalling in hepatocarcinoma, and slowed MCF7 xenograft growth.	^231–233^
**M3814 (30)** 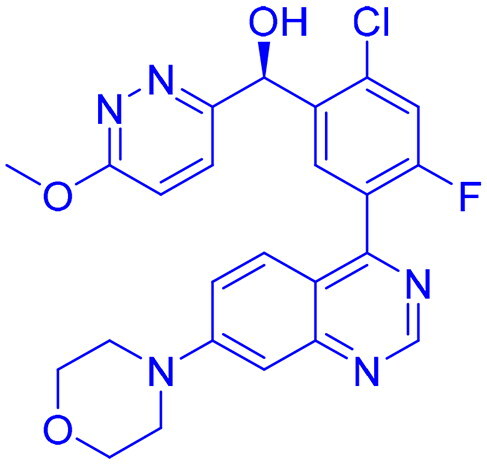	DNA-PK ATM CHK2	Fibrosarcoma, lung cancer, ovarian cancer, cervical cancer, head and neck cancer	M3814 induces cell death through mitotic catastrophe and apoptosis in A549 and HT-1080 cell lines. Additionally, M3814 reduces tumour growth in ovarian cancer cell lines. Induce radiosensitizing and antitumor activity in xenograft models	^234–237^
**CC-115 (31)** 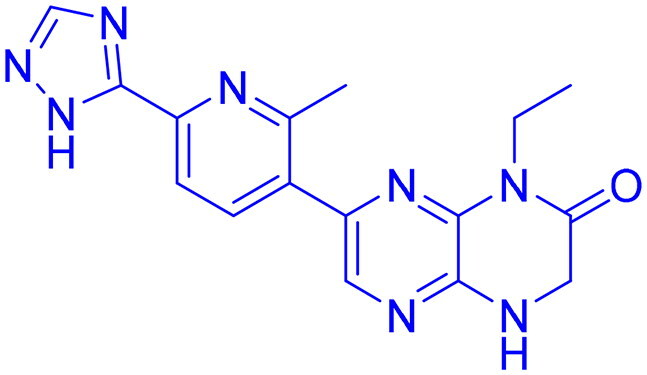	DNA-PK mTORC1/2	Metastatic castration-resistant prostate cancer, lung cancer	CC-115 and enzalutamide reduce the level of PSA in prostate cancer, producing a synergistic effect. CC-115 inhibited the activation of mTORC1/2 and DNA-PK, leading to the death of primary NSCLC cells.	^238^ ^,^ ^239^
**AZD7648 (32)** 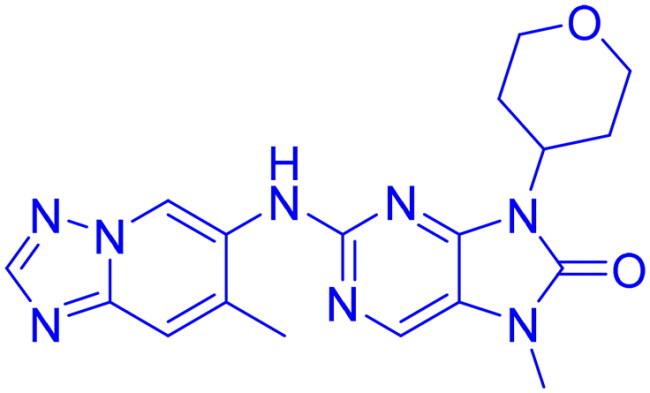	DNA-PK	Ovarian cancer leukaemia, head and neck cancer	Inhibiting abdominal metastases and tumour growth in a xenograft mouse model. Synergistic antiproliferative activity in the HL-60 xenograft model AZD7648 significantly enhances radiosensitivity in HNSC models	^240^ ^,^ ^241^
**XRD-0394 (33)** 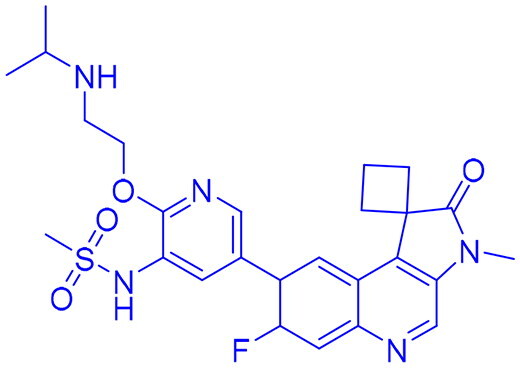	DNA-PK ATM	Solid tumour	XRD-0394 improves both in vitro and in vivo tumour cell death under ionising radiation conditions.	^91^

Overall, DNA-PK inhibition represents a growing field in cancer therapy, with ongoing research focused on improving drug properties, exploring combination therapies, and expanding treatment options for advanced or resistant cancers. Early inhibitors, such as quercetin **(25)** and LY294002 **(26)**, were non-selective and faced limitations like poor pharmacokinetics and toxicity. However, these inhibitors paved the way for more selective and potent inhibitors like NU7026 **(24)** and NU7441 **(28)**, which showed significant potential in sensitising cancer cells to radiation and chemotherapy across various cancer types. Despite their *in vitro* efficacy, these inhibitors struggled with suboptimal pharmacokinetics and bioavailability. Recent advancements have led to the development of more effective inhibitors, such as M3814, which has demonstrated substantial efficacy in clinical trials, enhancing cancer cell susceptibility to treatment while being well-tolerated. Other notable inhibitors, like CC-115 **(31)**, showed promise but were discontinued in some trials due to toxicity and limited efficacy. Newer inhibitors, including AZD7648 **(32)** and XRD-0394 **(33)**, are under clinical evaluation and have shown encouraging preclinical results.

## Interplay between ATM, ATR, and DNA-PKcs in cancer

ATM, ATR, and DNA-PKcs, key members of the phosphatidylinositol 3-kinase-like protein kinase (PIKK) family, are integral to the DNA damage response (DDR) at distinct levels. The instability of these proteins has been linked to the pathogenesis of numerous disorders, particularly numerous types of cancers^39^. While the precise mechanisms by which aberrations in ATM, ATR, and DNA-PK contribute to diverse phenotypic outcomes remain unclear, alterations in these genes are strongly linked with specific cancer subtypes. A detailed understanding of their involvement in DNA repair pathways and their complex crosstalk is important for developing effective therapeutic strategies^248^. Plenty of studies demonstrated that ATM, ATR, and DNA-PK are key proteins involved in the cellular response to DNA damage caused by factors like ionising radiation (IR), replication stress, or chemotherapy. When activated, these proteins initiate repair processes by interacting with other molecules to establish protein complexes that manage the repair of damaged DNA. These proteins play a fundamental role in rebuilding double-strand breaks (DSBs) and become important targets in cancer treatment strategies^249^. Multiple research findings suggest that inhibiting ATM or DNA-PK, particularly when combined with radiation or chemotherapy, significantly enhances the susceptibility of cancer cells to these treatments. This strategy proves especially effective in tumours harbouring BRCA1 or BRCA2 mutations, leveraging their intrinsic vulnerabilities attributed to the mechanism of synthetic lethality^47^. Several studies have explored the crosstalk between DDR pathways, with particular focus on the interplay between DNA damage response elements and chromatin non-repair elements, mediated by ATM, ATR, and DNA-PK^250^. Despite being activated by different types of cellular stress, these proteins share a common family origin as PI3-kinases and are deeply integrated into DNA repair pathways. After DSBs, ATM phosphorylates a range of cNREs, including Chk2, CtIP, SKP2, CDC25C, CDC25A, and p53, all critical for maintaining genomic stability. ATM also phosphorylates SMURF1, an E3 ubiquitin ligase, which recruits RNF8, another E3 ligase. RNF8 ubiquitylates H2AX through K63-linked conjugation to facilitate the recruitment of additional repair components^251–253^. Similarly, ATR phosphorylates several DNA damage response elements following replication stress, including CDC25A, GEMIN2, and TopBP1, which are involved in activating intra-S-phase checkpoints^254–256^. ATR also targets chromatin non-repair elements (cNRE) substrates like CHEK1 and BLM, which are essential to the homologous recombination (HR) repair pathway. CHEK1 phosphorylates NSMCE2 to ensure ZBRK1-dependent repression of HDR, thus favouring non-homologous end joining (NHEJ) over HR^257–260^. In contrast, CHEK1 counteracts PALB2’s action on RAD51 within the HR pathway^261^. CHEK1, a key downstream target of ATR in response to replication stress, undergoes phosphorylation and activation by ATR to arrest S-phase progression and resolve stalled replication forks. In the context of double-strand break (DSB) repair, DNA-PK phosphorylates an extensive spectrum of substrates, including NBS1, Rad50, H2AX, and TP53BP1, all of which play pivotal roles in the primary stages of DSB repair, chromatin remodelling, and maintaining genomic stability. Additionally, DNA-PK regulates chromatin accessibility to facilitate efficient DNA repair processes^262–264^. Moreover, DNA-PK participates in the crosstalk between DSB repair pathways and the DNA damage transcriptional machinery. Active DNA-PK recruits RNF8 for ubiquitylation events, including the degradation of p53 early in the DNA damage response, thereby inhibiting the transcription of downstream repair genes^265^^,^^266^. Takamitsu A. Kato *et.al*. indicate that in mammalian cells, a strong synthetic lethal effect occurs when ATM, ATR, and DNA-PKcs are all inhibited at the same time. While suppressing one or two of these proteins can increase cell death, blocking all three leads to a much higher accumulation of DNA double-strand breaks (DSBs), severe chromosomal damage, and significantly greater cell death^267^. In castration-resistant prostate cancer lacking functional ATM, the absence of ATM drives a dependency on ATR and DNA-PK for the DNA damage response. Concurrent inhibition of these kinases markedly amplifies radiosensitivity, resulting in elevated cell death and a greater reduction in DNA repair markers, such as γH2AX foci, compared to monotherapy. Moreover, this dual-targeted approach disrupts replication fork dynamics, further enhancing the vulnerability of cancer cells to radiation-induced damage^268^. Studies utilising mouse models that exhibited kinase-dead variants of ATM, ATR, and DNA-PK have demonstrated the key structural roles of these kinases at sites of DNA damage. Notably, these models exhibited more pronounced genomic instability than models with complete kinase deletion, suggesting that catalytic inhibition results in the persistent localisation of these kinases at damage sites. The study reveals that in S-phase irradiated cells, ATM and DNA-PK collaboratively destroy hyperactivation of the G2 checkpoint, with inhibition of either kinase producing similar effects. While ATM and ATR work together in G2, ATR primarily regulates the S-phase checkpoint, supported by ATM. Deficiencies in these kinases can significantly impact cancer cells; DNA-PK deficiency increases DNA end resection and causes hyperactivation of the ATR-dependent G2 checkpoint, while ATM deficiency reduces resection but may lead to prolonged checkpoint activation^71–77^^,^^269^^,^^270^.

## ATM, ATR, DNA-PKcs inhibitors in clinical trial lead compounds

Numerous inhibitors of ATM, ATR, and DNA-PK have advanced into clinical trials for oncological applications, as summarised in Table 4. CC-115 (31), a DNA-PK inhibitor, is currently being evaluated in a Phase I clinical trial for patients with brain and different malignancies^271^. Berzosertib (VE-822, 19), a highly selective ATR inhibitor, has demonstrated significant efficacy in suppressing pancreatic tumour progression while exhibiting minimal off-target toxicity in normal tissues. Ongoing clinical investigations are exploring the therapeutic potential of Berzosertib in combination with the platinum-based chemotherapeutic agent cisplatin in a Phase I trial for advanced solid tumours. Preliminary data suggest that Berzosertib, both as a monotherapy and in combination with cisplatin, is generally well-tolerated with an acceptable safety profile^272–275^. AZD0156, with an IC_50_ of 0.58 nM, disrupts DNA damage response (DDR) mechanisms and induces apoptosis in ATM-overexpressing tumours. Its pharmacokinetics, safety, tolerability, and efficacy are under investigation in oncology trials. AZD0156 enhances radiation-induced tumour growth inhibition, potentiates PARP inhibitors like olaparib, and shows synergistic effects with irinotecan, demonstrating promise in combinatorial cancer therapies^276^^,^^277^. VX-803, another potent ATR kinase inhibitor, effectively inhibits the phosphorylation of PeCHK1 with an IC_50_ of 8 nM. M4344, in combination with niraparib, is also being evaluated in early-phase clinical trials for patients with PARP-resistant persistent ovarian carcinoma^278^^,^^279^. LY294002 (26), a DNA-PK inhibitor, was investigated for neuroblastoma in a Phase I trial that was terminated due to insufficient patient enrolment. Another clinical trial is currently assessing the safety, tolerability, and pharmacokinetics/pharmacodynamics (PK/PD) of tuvusertib in combination with lartesertib (M4076) and, in some cohorts, with avelumab. This study aims to determine the maximum tolerated dose (MTD), identify the recommended expansion dose, and evaluate preliminary signs of clinical efficacy in specific cancer types. Additionally, it investigates the bioavailability of various tuvusertib formulations^280^^,^^281^. AZD7648 (32), with an IC_50_ of 0.6 nM in biochemical assays and over 100-fold selectivity against other kinases, is being explored in a Phase II clinical trial for oncological applications. VX-984 (37), a potent DNA-PK inhibitor, effectively inhibits non-homologous end joining (NHEJ), thereby preventing the repair of double-strand breaks (DSBs) induced by chemotherapeutic agents or ionising radiation (IR). Clinical studies have evaluated VX-984 both as a monotherapy and in combination with the PARP inhibitor niraparib for breast cancer^284^. Nedisertib (30) (M3814), a highly potent DNA-PK kinase inhibitor, is known to sensitise various cancer cell lines to DSB-inducing agents and IR. Multiple clinical trials are actively investigating Nedisertib as a standalone treatment and in combination with radiotherapy and chemotherapy^285^^,^^286^. BAY-1895344 (Elimusertib), an orally bioavailable ATR kinase inhibitor, is being extensively studied for its antineoplastic activity in preclinical and clinical settings, demonstrating promising results in various malignancies. BAY-1895344, in combination with the PARP inhibitor niraparib, is currently being evaluated in early-phase clinical trials for patients with advanced solid and ovarian carcinomas. Among 22 patients treated with this combination, a partial response was observed in four cases, with a median survival duration of 315 days in individuals with ATM protein mutations or loss of ATM expression. The most common treatment-related adverse events included anaemia, neutropenia, and thrombocytopenia. Overall, BAY-1895344 exhibits compelling anti-tumour efficacy, particularly in solid malignancies with ATM pathway deficiencies^287^. Similarly, AZD6738, an orally administered and highly potent ATR inhibitor, is being evaluated in a Phase I clinical trial in combination with paclitaxel for patients with advanced solid tumours and non-small cell lung cancer (NSCLC). Interim results from 57 patients treated with AZD6738 (240 mg BID) plus paclitaxel demonstrated an overall response rate of 22.6%, with the melanoma subgroup achieving a 33.3% objective response rate, a median progression-free survival of 3.6 months, a duration of response of 9.9 months, and an overall survival of 7.4 months. A separate Phase III clinical trial is assessing the efficacy and safety of AZD6738 in combination with durvalumab compared to standard-of-care docetaxel in patients with advanced or metastatic NSCLC who have progressed following prior anti-PD-(L)1 therapy and platinum-based chemotherapy^289^. Additionally, an ongoing clinical study is evaluating the efficacy and safety of ART0380 as a monotherapy in patients with tumours exhibiting biological characteristics predictive of sensitivity to ATR inhibition. WSD0628 (34), a potent ATM inhibitor, is currently under investigation in a Phase I clinical trial in combination with radiotherapy for the treatment of persistent brain tumours. The primary objectives of this study include evaluating the safety, tolerability, pharmacokinetics (PK), and preliminary anti-tumour efficacy of WSD0628 when administered with radiation therapy^290^.

**Table 4.** Various ATM, ATR, DNA-PKcs inhibitors in clinical trial studies.

Candidate drug	Target	Type of cancer	Stage of clinical trials	Outcomes of clinical study	Time period of clinical trials	References
**CC-115 (31)** 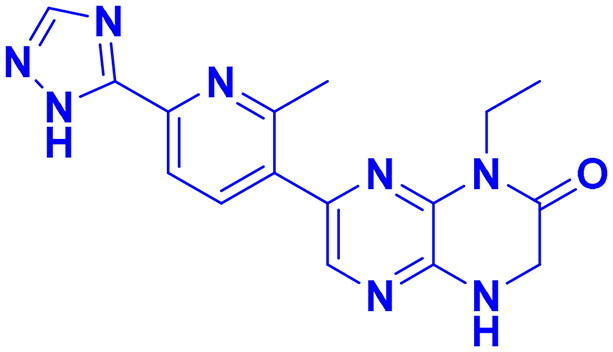	DNA-PK	Advanced solid tumours, and haematologic malignancies	Completed Phase 1	CC-115 exhibited anticancer efficacy in solid tumours by overcoming resistance mechanisms and inducing tumour regression. With a manageable toxicity profile and promising clinical activity, including partial responses across various cancer types	April 2011 to March 2021	^271^
**VE 822 (19)** 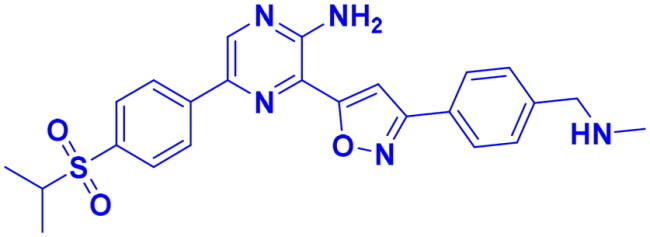	ATR	Advanced solid tumours	Completed Phase 1	VE 822 showed a manageable safety profile and predictable PK with cisplatin or gemcitabine, without significant drug-drug interactions. The established RP2Ds and preliminary efficacy support further Phase 2 trials, particularly in combination with platinum-based therapies or gemcitabine.	December 2012 to March 2020	^177^ ^,^ ^272–275^
**AZD0156 (6)** 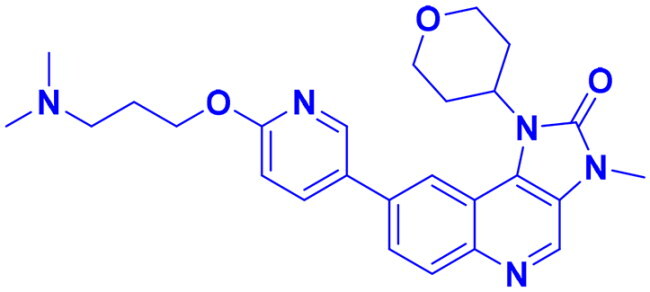	ATM	Advanced cancer	Completed Phase 1	The safety and preliminary efficacy of AZD0156 were evaluated alone or in combination with other anti-cancer treatments in patients with advanced cancer. AZD0156 enhanced the tumour growth inhibitory effects of radiation treatment and potentiates the effects of PARP inhibitors like Olaparib as well as showed synergistic effects with irinotecan in this study	November 2015 to July 2022	^276^ ^,^ ^277^
**Gartisertib (M4344) (16)** 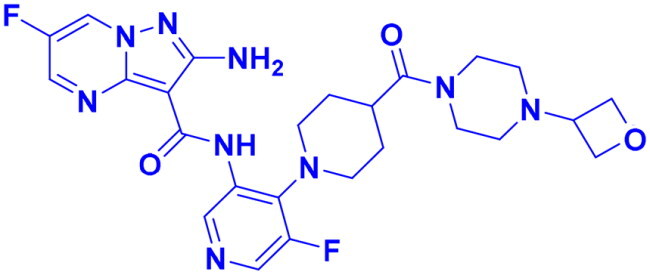	ATR	Ovarian cancer	Withdrawn Phase 1	M4344 showed a manageable safety profile, predictable pharmacokinetics, and preliminary anti-tumour activity in advanced solid tumours. The established MTD and observed clinical activity support further investigation in larger trials, particularly in combination therapies.	May 2023 to May 2024	^278^ ^,^ ^279^
**LY294002 (26)** 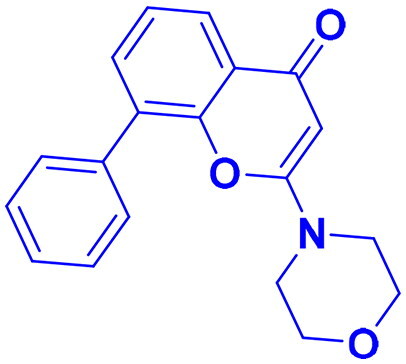	DNA-PK	Neuroblastoma	Terminated Phase 1	Insufficient enrolment or recruitment of participants into a clinical trial,	July 2015 to May 2018	^280^
**Samotolisib (36)** 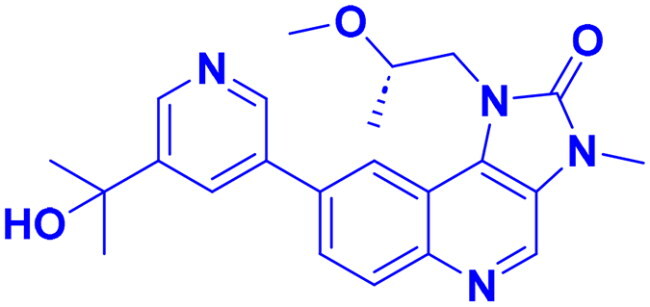	DNA-PK	Advanced malignancies	Completed Phase 1	The results of the study have not yet been submitted for publication.	September2015 to February 2017	^281^
**AZD7648 (32)** 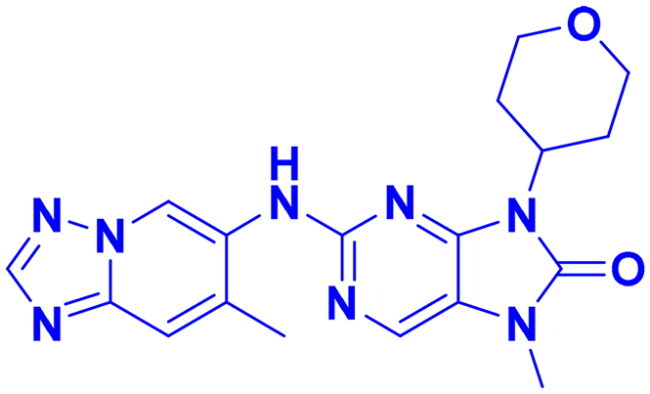	DNA-PK	Advanced malignancies	Completed Phase 1 Phase 2	Partial responses (PR) and stable disease (SD) were observed in patients with squamous head and neck cell carcinoma, Ewing sarcoma, glioblastoma multiforme, and castration-resistant prostate cancer. 10 mg twice daily of AZD7648 show stability and tolerability in Phase 2 of clinical study	October 2019 to December 2022	^282^
**AZD1390 (7)** 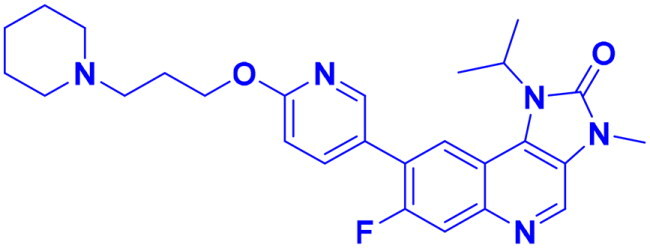	ATM	NSCLC	Recruiting Phase 1	The trial has completed its recruitment phase and is in the data analysis stage. The results are not posted and expected to be published in the coming months.	March 2021 to March 28, 2028 (estimated)	^283^
**VX-984 (37)** 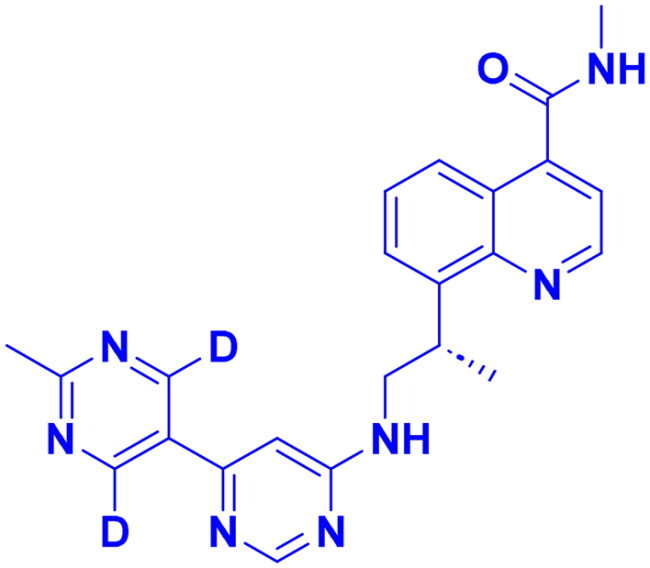	DNA-PK	Advanced tumours	Withdraw Phase 1 Phase 2	The trial was withdrawn based on portfolio prioritisation	December 2020 to September 2023	^284^
**Nedisertib (30)** 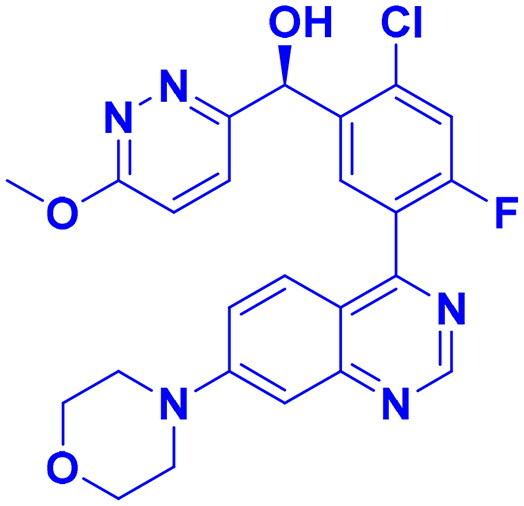	DNA-PK	Pancreatic neuroendocrine, tumours ovarian cancer	Active Phase 1 Active Phase 1	No results have been posted for this study as it is still ongoing and not yet completed.	July 2021 to March 2026 (estimated) May 2020 to June 2025 (estimated)	^285^ ^,^ ^286^
**BAY 1895344 (35)** 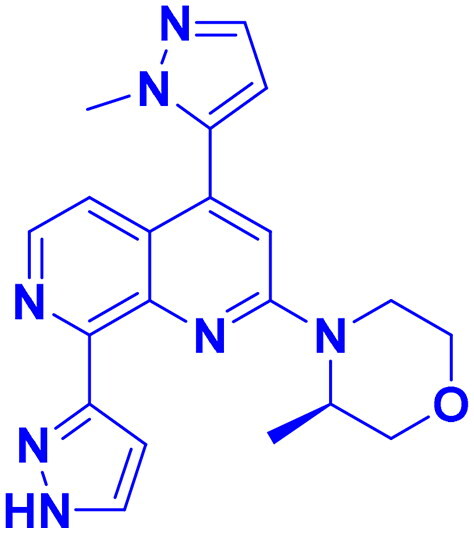	ATR	Advanced or metastatic cancers of the stomach and intestines	Active, Phase 1	No results have been posted for this study as it is still ongoing and not yet completed.	August 2021 to April 2025 (estimated)	^287^
**Lartesertib (11)** 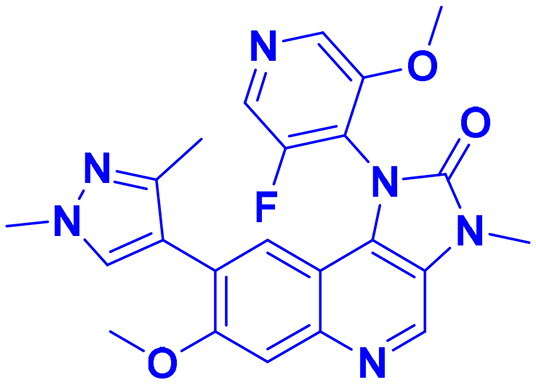	ATM	Solid tumour	Recruiting Phase 1	The clinical study is under process, no result has been posted yet	June 2022 to May 2026 (estimated)	^288^
**AZD6738 (21)** 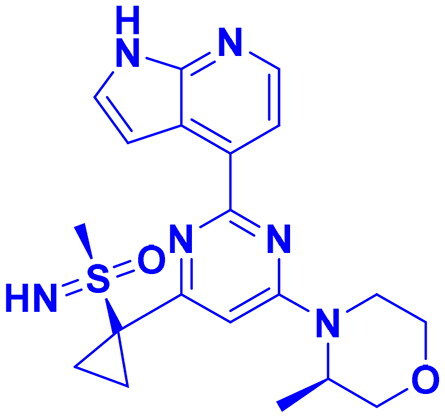	ATR	NSCLC	Active Phase 3	No results have been posted for this study as it is still ongoing and not yet completed.	September2022 to August 2025 (estimated)	^289^
**WSD0628 (34)** 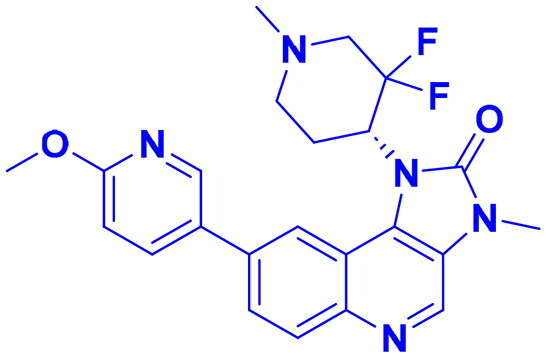	ATM	Glioblastoma	Recruiting Phase 1	No results have been posted for this study as it is still ongoing and not yet completed.	January 2024 to February 2029 (estimated)	^290^

Structures were drawn by the authors using Chemdraw software.

## Patented literature on ATM, ATR, DNA-PK inhibitors

DNA-PK, ATR, and ATM inhibitors have displayed significant potential effects in preclinical studies as targeted therapies for cancer (Table 5). In 2025, Jazz Pharmaceuticals patented an ATR-PARP inhibitor combination designed to overcome PARP resistance by disrupting DNA repair in cancer cells. In the same year, Celator Pharmaceuticals introduced an ATR inhibitor delivered via a liposomal drug delivery system to enhance treatment efficacy for refractory malignancies. Another key development was from Antengene Discovery Limited in 2022, which patented a novel class of ATR inhibitors with improved potency, selectivity, and pharmacokinetic properties for treating NSCLC and SCLC. Additionally, Sichuan University (2021) developed ATR inhibitors that exhibited significant anti-proliferative effects in LoVo cells and showed potent activity against colorectal tumours in animal models. Other patents target different cancer types. In 2023, Nanjing Damei Biopharmaceutical developed ATR inhibitors particularly effective against cancers with impaired DNA repair mechanisms, including those with BRCA1/2 mutations, PTEN loss, or ATM deficiency. Similarly, Wigen Biomedicine Technology (2023) introduced naphthyridine derivatives as ATR kinase inhibitors for treating ATR-mediated diseases, including breast and solid tumours. Shanghai Antengene Corporation Limited (2022) synthesised heteroaromatic ATR inhibitors, with one compound demonstrating exceptional potency, exhibiting an IC_50_ value below 100 nM. Some patents focus on multi-targeted inhibitors. AstraZeneca (2017) developed imidazo[4,5-c]quinolin-2-one derivatives that inhibit ATR, ATM, and mTOR kinases, demonstrating potential for treating breast cancer and ductal carcinoma. Jiangsu Hengrui Medicine (2022) patented an imidazopyrimidine derivative targeting ATR, DNA-PK, and PI3K, showing promise for colon cancer treatment. Meanwhile, CSPC Zhongqi Pharmaceutical Technology (2022) introduced ATM inhibitors with significant selectivity for ATM kinase, making them a potential treatment for solid and hematological tumours. Medshine Discovery (2021) also patented ATM inhibitors with strong anticancer activity and excellent selectivity for the ATM target. Additionally, some patents focus on PI3K and DNA-PK inhibition. Yamanouchi Pharmaceutical (2001) synthesised imidazopyridine derivatives as PI3K inhibitors, demonstrating strong anti-proliferative effects against melanoma with IC_50_ values consistently below 1 μM. Finally, Beijing Tide Pharmaceutical patented pyrazolopyrimidine-based ATR inhibitors that have demonstrated exceptional anticancer activity against multiple malignancies, further reinforcing ATR kinase inhibition as a promising therapeutic strategy. These patented compounds collectively highlight a growing trend in ATR, ATM and DNA-PK targeted cancer therapies, with increasing emphasis on combination approaches, improved drug delivery methods, and multi-targeted kinase inhibition to enhance treatment efficacy and overcome resistance mechanisms^291–302^.

**Table 5.** Patented ATM, ATR, DNA-PK inhibitors.^291–302^

Compound	Target	Type of cancer	Publication year	Company/assignees	References
**38** 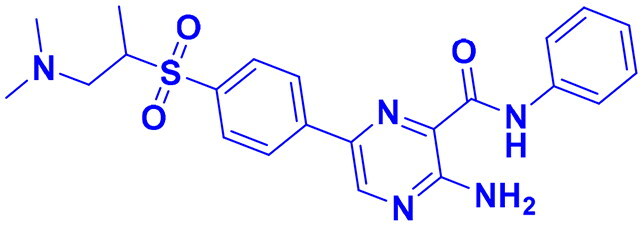	ATR	Advanced tumour	2025	Jazz Pharmaceuticals, United States	^291^
**39** 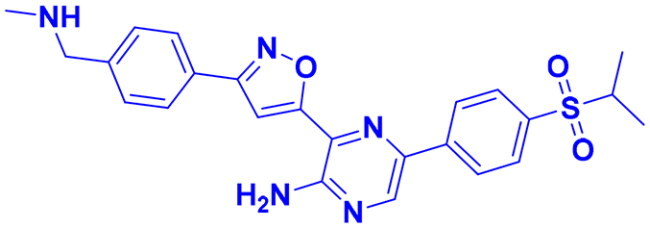	ATR	Advanced tumour	2025	Celator Pharmaceuticals, United States	^292^
**40** 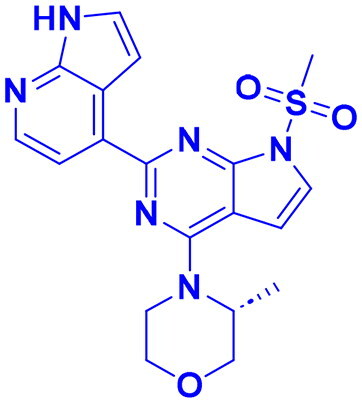	ATR	Brain cancer	2023	Nanjing Damei Biopharmaceutical, China	^293^
**41** 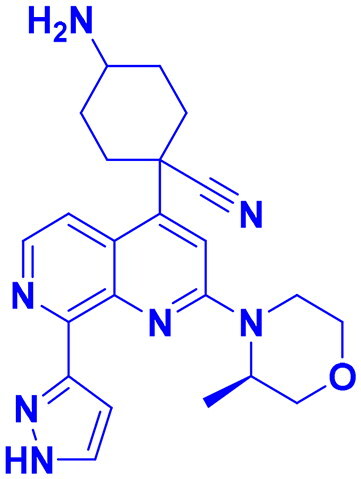	ATR	Solid and breast tumour	2023	Wigen Biomedicine Technology, China	^294^
**42** 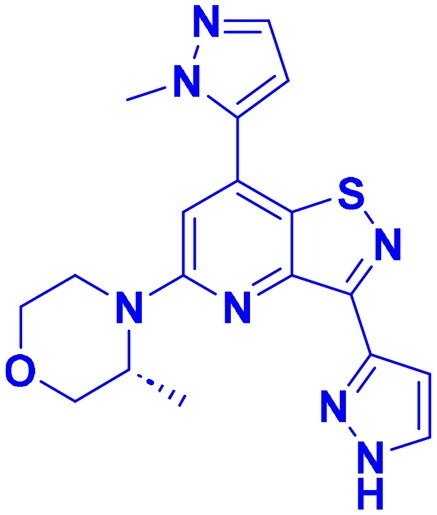	ATR	NSCLC, SCLC	2022	Antengene Discovery Limited, China	^295^
**43** 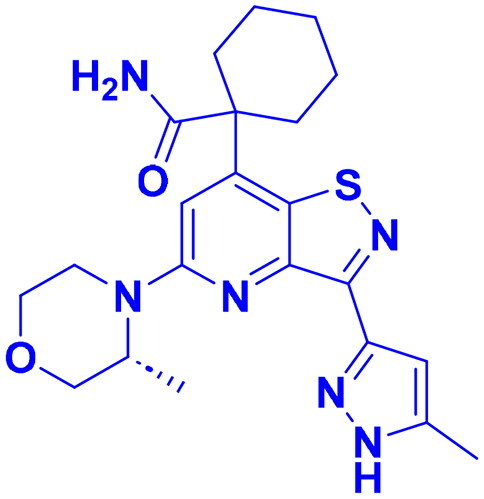 **44**	ATR,	Colon cancer	2022	Shanghai Antengene Corporation Limited, China	^295^
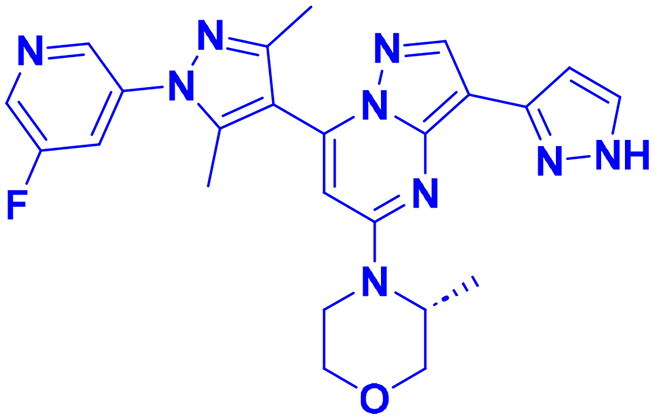	ATR	Multiple malignancies	2022	Beijing Tide Pharmaceutical, China	^296^
**45** 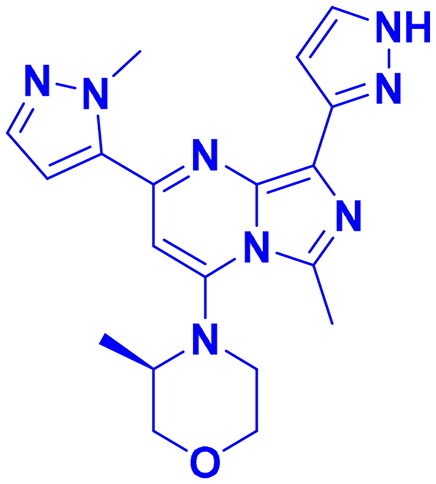	ATR, DNA-PK, PI3K	Colon cancer	2022	Jiangsu Hengrui Medicine, China	^297^
**46** 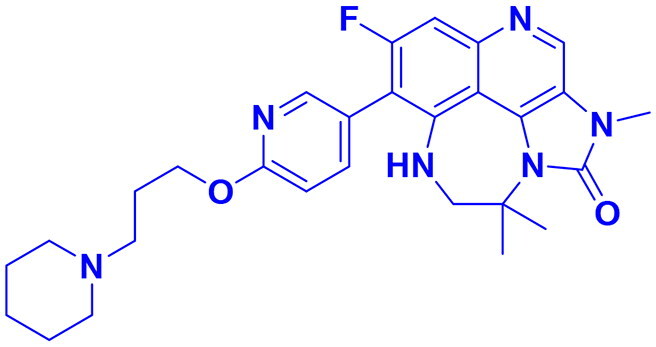	ATM	Solid & hematological tumour	2022	CSPC Zhongqi Pharmaceutical Technology, China	^298^
**47** 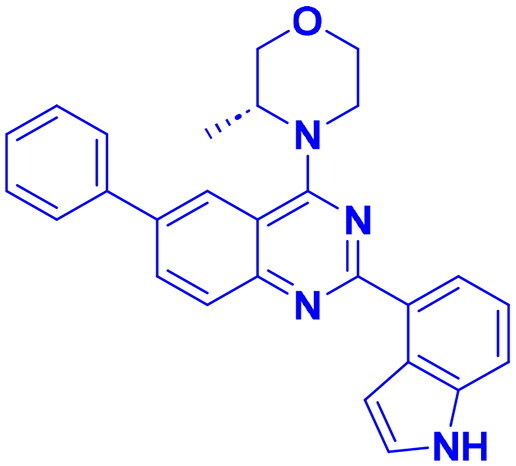	ATR	Colorectal cancer	2021	Sichuan University, China	^299^
**48** 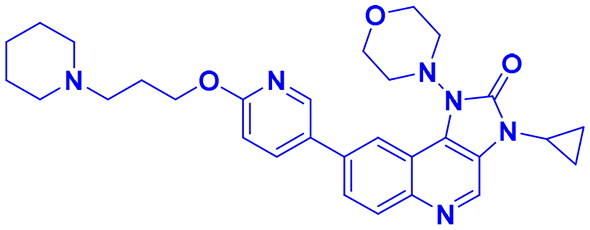	ATM	Solid tumour & neurological diseases	2021	Medshine Discovery, China	^300^
**49** 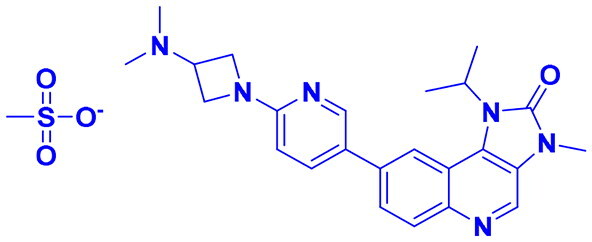	ATR, ATM, mTOR	Ductal carcinoma, breast cancer	2017	AstraZeneca, England	^301^
**50** 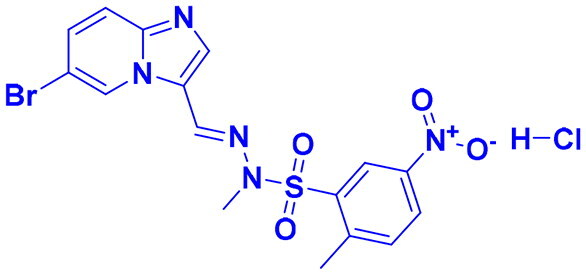	DNA-PK	Melanoma	2001	Yamanouchi Pharmaceutical, Japan	^302^

Structures were drawn by the authors using Chemdraw software.

## Recent medicinal chemistry campaigns

Medicinal chemists have made significant strides in leveraging key biological discoveries about the roles of DNA-PK, ATR, and ATM in the initiation and progression of cancer to design tractable chemical architectures exerting their modulation. Numerous rationally designed compounds targeting these critical pathways are currently under investigation in early-stage preclinical studies. This segment also highlights the conformational relationships among these inhibitors are shaped by their structural frameworks, binding interactions, and modifications that enhance their selectivity and pharmacokinetics. ATM, ATR, and DNA-PK inhibitors share core scaffolds like quinolone, cinnoline, imidazoquinolinone, benzimidazole, pyrimidine, and urea-based structures. Structural rigidification, scaffold hopping, and linker optimisation have played a key role in refining their activity. For example, ATM inhibitors evolved from early quinolone derivatives, which exhibited poor pharmacokinetics, to cinnoline carboxamides with improved permeability and bioavailability. Structural modifications, such as dimethyl amino propyloxy side chains, strengthened interactions with polar sub-pockets, improving potency and selectivity. Similarly, rigidification strategies in compounds like AZD0156 enhanced ATP-binding affinity and stability. ATR inhibitors initially lacked selectivity, but pyrimidine core optimisation, along with the incorporation of morpholino and sulphonyl groups, improved solubility and kinase specificity. DNA-PK inhibitors, like chromen-4-one derivatives, benefitted from morpholino groups for better hydrogen bonding, while dibenzothiophene substitutions enhanced activity. These modifications stabilised inhibitor conformations, allowing for tighter kinase binding. The insights gained from both *in vitro* and *in vivo* evaluations provide a comprehensive understanding of the therapeutic potential of these novel ATR, DNA-PK, and ATM inhibitors.

### Medicinal chemistry endeavours on ATM & ATR inhibitors

**Figure 5.**
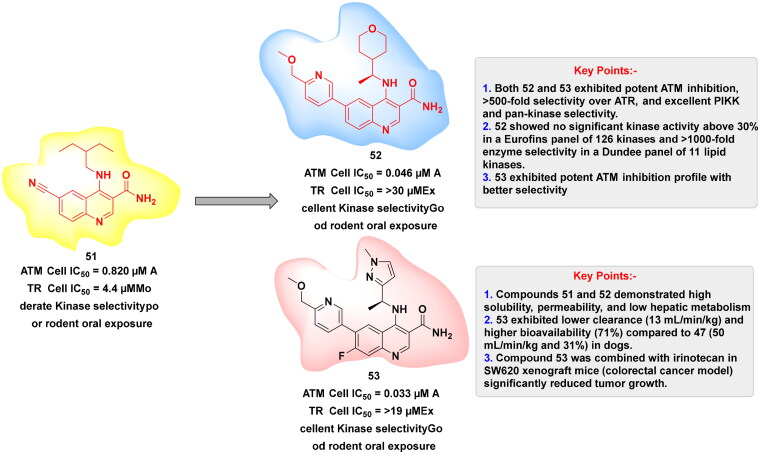
Quinoline carboxamides as ATM inhibitors (the figure was drawn by the authors using Chemdraw software).

In the quest to furnish potent ATM inhibitors as antitumor agents endowed with impressive pharmacokinetic profiles, Degorce *et al* conducted a structural exploration of a modestly potent HTS hit containing the quinolone core **(51).** The quinolone core was comprehensively explored at every single substitution point. Resultantly, two structures (**52** and **53**) were identified as highly potent ATM inhibitors (Figure 5). In addition to magnificent potency, both chemical architectures also exhibited substantial selectivity towards ATM over ATR. In particular, compound **53** inhibited only 3 kinases (CLK1, 57%; CLK4, 55%; Mer, 56%) in a panel of 386 kinases. Inspired by the striking potency as well as selectivity of ATM inhibitors **52** and **53**, both compounds were subjected to pharmacokinetics study in rats and dogs. Gladly, the results of the pharmacokinetic study were quite optimistic as bioavailability, as well as clearance, were observed to be within the stipulated range for both the compounds in both species. In particular, compound **53** demonstrated lower clearance and higher bioavailability than **52** in dogs. *In-vivo* antitumor activity evaluation of **53 (**50 mg/kg) was performed in combination with irinotecan tumour-bearing, immunocompromised mice. It was observed that the drug cocktail exerted a more pronounced reduction of tumour growth than irinotecan^303^.

**Figure 6.**
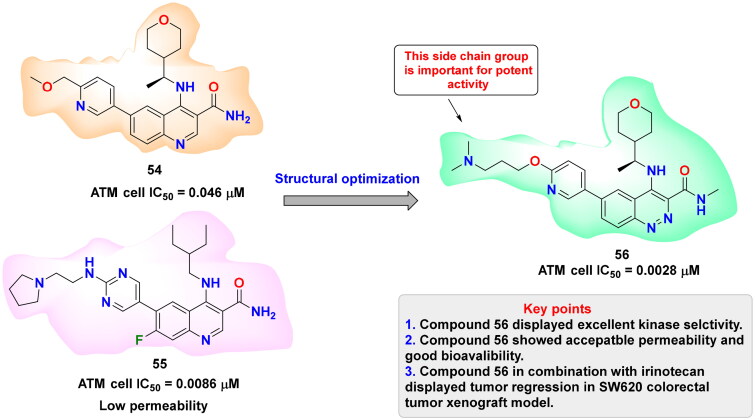
Cinnoline carboxamides based selective ATM inhibitors (the figure was drawn by the authors using Chemdraw software).

Early inhibitors of ATM such as Ku-55933 **(2)** and Ku-60019 **(3)**, demonstrated strong activity but were hindered by poor physicochemical properties and limited oral bioavailability^108^. Advancements in the ATM inhibitor development field have enabled the identification of compounds with improved selectivity, potency, and pharmacokinetic profiles, for example, quinoline carboxamides and imidazo[5,4-c]quinolin-2-ones, as well as cinnoline carboxamides. To address issues related to pharmacokinetics and low potency, Barlaam *et al* designed and synthesised a novel series of 3-cinnoline carboxamides as highly potent and selective inhibitors of ataxia-telangiectasia mutated (ATM) kinase (Figure 6). A comparative analysis of cinnoline carboxamides with the quinolone counterparts was conducted in the study. The findings of the study showed that cinnoline carboxamides exhibited higher permeability and lower efflux compared to their quinoline counterparts, along with favourable metabolic stability and oral bioavailability. Structure-activity relationship (SAR) studies revealed that certain modifications, such as the introduction of dimethyl amino propyloxy side chains, significantly enhanced ATM potency by interacting with polar sub-pockets in the kinase’s active site. Among all tested compounds, compound (**56)** stood out, demonstrating exceptional kinase selectivity against a wide range of kinases and favourable pharmacokinetic properties, including long half-lives, high oral bioavailability, and good solubility. Previous studies have shown that combining ATM inhibitors with DNA-damaging drugs, such as topoisomerase I inhibitors leads to synergistic anti-tumour efficacy. Thus, the effect of a combination of compound (**56)** with irinotecan was investigated and it was observed that the drug cocktail significantly improved tumour growth inhibition compared to irinotecan alone. These findings highlight the therapeutic potential of ATM inhibitors as integral components of combination treatment strategies in oncology^304^.

**Figure 7.**
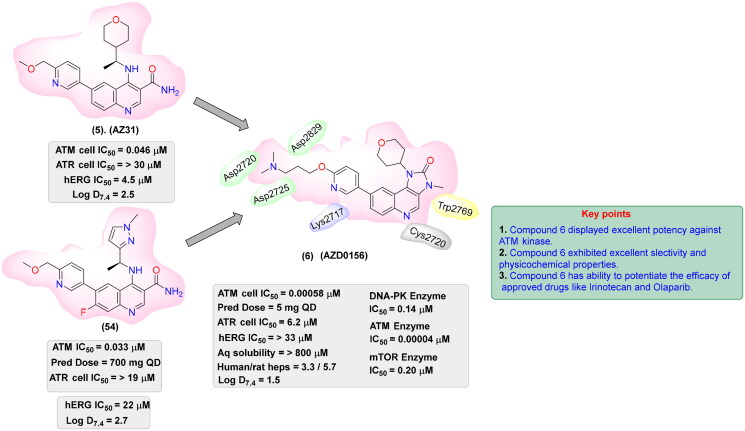
Discovery of first-in-class AZD0156 as ATM kinase inhibitor (the figure was drawn by the authors using Chemdraw software).

Pike et al. conducted a lead optimisation study employing their previously furnished ATM inhibitor **(54)** as the starting point. Compound **(54)** demonstrated strikingly potent and selective inhibitory potential towards ATM and was also found to be suitable for oral administration. Despite being endowed with impressive ATM inhibitory and anticancer profiles, the relatively short human half-life and high clinically efficacious dose of **(54)** urged the research group to conduct a structural engineering program in pursuit of preserving or enlightening potency, selectivity, and physiochemical properties. The structural alteration program identified **(6)** (AZD0156) possessing an imidazo[4,5-c]quinolin-2-one core endowed with a magnificent ATM inhibitory profile (IC_50_ = 0.00058 µM) (Figure 7). Computational analyses demonstrated that compound **6** exhibits strong binding affinity to the ATP-binding site of ATM by interacting with key residues, including the kinase hinge region, the catalytic lysine, and the back pocket. The basic amine group was strategically positioned within a highly polar sub-pocket of the ATM binding site, effectively interacting with the surrounding acidic residues. In the *in vivo* studies, compound **(6)** potentiated the ability of DNA DSB-inducing agents. Notably, a combination of **(6)** (20 mg/kg) and irinotecan (50 mg/kg) led to tumour regression in a SW620 xenograft model in immune-compromised mice. Also, a drug cocktail of **(6)** (5 mg/kg) and olaparib (50 mg/kg) caused tumour regression in immunocompromised mice enduring HBCx-10 patient-derived tumours. Further, the pharmacokinetic study of compound **(6)** was conducted in rats and dogs and it was observed that the inhibitor **(6)** exhibited good oral bioavailability and had low to moderate clearance. Notably, the outcome of the *in vitro* metabolite identification study revealed that the key route of metabolism for **(6)** in humans was probable to be flavin-containing monooxygenase (FMO) mediated N-oxidation of the basic nitro-gen moiety, steering towards the generation of a metabolite that retained the activity against ATM. Compound **(6)** is presently undergoing clinical evaluation with these agents^305^.

**Figure 8.**
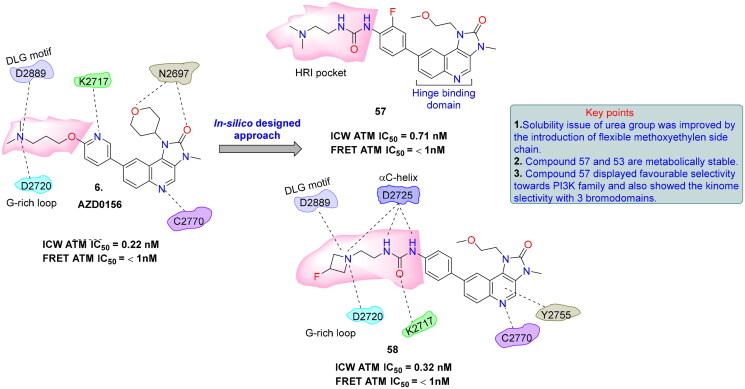
Development of novel urea-based ATM kinase inhibitors (the figure was drawn by the authors using Chemdraw software).

Dimitrov et al. utilised *in-silico* methods like induced fit docking, MD simulations, and homology modelling for the development of urea-based ATM kinase inhibitors for the treatment of cancer. A series of ATM kinase inhibitors were designed and synthesised. Compound **(6) (AZD0156)** was initially optimised by replacing ether linkage to urea for further interaction with the aspartate residues within the HRI pocket of ATM kinase. To overcome the solubility limitations associated with the urea linker, structural modifications were explored by introducing a flexible methoxyethylene chain to the 1,3-dihydro-2H-imidazo[4,5-c]quinolin-2-one core, coupled with the replacement of the pyridine moiety with a phenyl group. These modifications yielded derivatives with enhanced metabolic stability and improved solubility. Among the synthesised analogs, compounds **(57)** and **(58)** exhibited potent sub-nanomolar inhibitory activity against ATM, with ATM IC_50_ values of 0.71 nM and 0.32 nM, respectively, in cellular assays conducted on A549 cells (Figure 8). Furthermore, both compounds demonstrated inhibitory concentration values of ≤1 nM in FRET-based assays, comparable to the reference compound **(6).** Notably, compounds **(57)** and **(58)** displayed high selectivity across the PIKK and PI3K families, with no significant off-target interactions observed against related kinases or bromodomains. Additionally, compounds **(57)** and **(58)** displayed comparable metabolic stability to reference compound **(6).** Furthermore, it was observed that the urea linker displayed additional hydrogen bond interaction with the D2725 αC-helix motif^306^.

**Figure 9.**
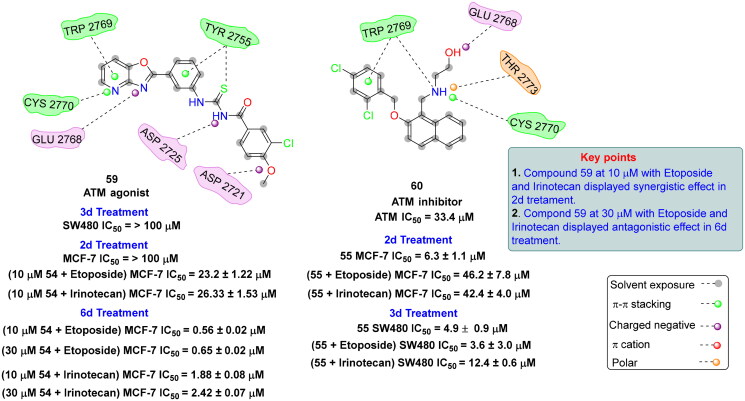
Discovery of novel ATM kinase modulators (the figure was drawn by the authors using Chemdraw software).

Zhang et al. performed structure-based virtual screening and extensive biological studies to design ATM ligands and the endeavour led to the identification of two distinct compounds: **59**, a selective ATM agonist, and **60**, a potent ATM inhibitor. Compound **59** was characterised as a low-toxicity agonist that enhanced the phosphorylation of ATM protein and its downstream target, KAP1, in response to DNA-damaging molecules like etoposide. In contrast, compound **60** exhibited strong antiproliferative effects in cancer cells. Intriguingly, compound **60** demonstrated time-dependent dual effects when prescribed with chemotherapeutic drugs such as irinotecan and etoposide. During short-term treatments, **60** antagonised chemotherapies by suppressing the phosphorylation of p53 and p21. This suppression accelerated cell cycle progression, improved cell survival, and reduced chemotherapy efficacy. However, with long-term exposure, compound **60** synergized with etoposide and irinotecan, enhancing its antiproliferative effects and sensitising cancer cells to treatment. Notably, compound **60** was particularly effective against tumours with p53 deficiency or mutations, which are prevalent in cancers such as lung, breast, and colon cancers. *In vivo,* studies revealed that compound **60** enhanced the antitumor activity of irinotecan in MCF-7 and SW480 xenograft model and without causing weight loss in mice. The binding modes of **59** and **60** were investigated using molecular docking and molecular dynamics (MD) simulations. Computational studies demonstrated that other ATM inhibitors CP466722, AZD-0156, AZ31, AZ32, and KU55933, exhibited comparable binding patterns within the ATP binding site, forming hydrogen bonds and hydrophobic interactions with amino residues (Figure 9). Compound **60**, a distinct ATM inhibitor, exhibited unique features, including hydrogen bonds and a π-π stacking interaction with amino acid residues, with its 2,4-dichlorobenzyl group deeply buried in a hydrophobic pocket. Unlike inhibitors, the ATM agonist, compound **59** demonstrated a unique mechanism, forming hydrogen bonds with Lys2717 and Asp2725, crucial for ATM activation, along with additional interactions involving the oxazolo[4,5-b]pyridine scaffold and hydrophobic residues^307^.

**Figure 10.**
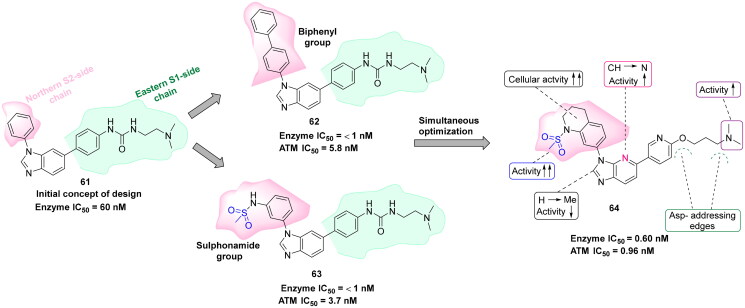
Novel benzimidazole and Imidazo[4,5-*b*]pyridine-based ATM kinase inhibitors (the figure was drawn by the authors using Chemdraw software).

Dimitrov et al. designed and synthesised a novel series of ­benzimidazole and Imidazo[4,5-*b*]pyridine-based ATM (ataxia-telangiectasia mutated) kinase inhibitors with sub-nanomolar activity. The authors utilised structure-activity relationship analysis based on the previously reported quinoline-based derivatives with the subsequent optimisation on the two side chains (northern S2-sidechain/eastern S1-sidechain) of the early hit compound **(61)** (ATM IC_50_ = 60 nM). Based on the early hit, a group of biphenyl and sulfonamide-based inhibitors were identified, with low cellular activity and modest-to-low selectivity against mTOR and DNA-PK. Simultaneous optimisation and implementation of different approaches on biphenyl and sulfonamide-based inhibitors led to the development of a compound **(64)** that displayed improved ATM inhibitory activity (IC_50_ = 0.96 nM) and binding affinity (Figure 10). Importantly, the kinase assay was carried out, which displayed that compound **(64)** had good selectivity within the PIKK and PI3K kinases with residual activity over 80% at an inhibitor concentration of 0.1 μM. Additionally, compound **(64)** displayed good metabolic stability at 23% (t_1/2_ = 59/CL_int_ = 12) and favourable pharmacokinetic properties with C_max_ = 42 nM. Moreover, the immunofluorescence assay on A549 cells confirms that compound **(64)** effectively reduced the phosphorylation levels of H2AX compared to etoposide-treated cells. Western blot assay revealed that compound **(64)** significantly reduced the phosphorylation of CHK2 and p53. Furthermore, it was found that compound **(64)** strongly inhibited the overexpressed murine hepatocellular carcinoma organoids (MLO-6/MLO-3) with different concentrations of etoposide^308^.

**Figure 11.**
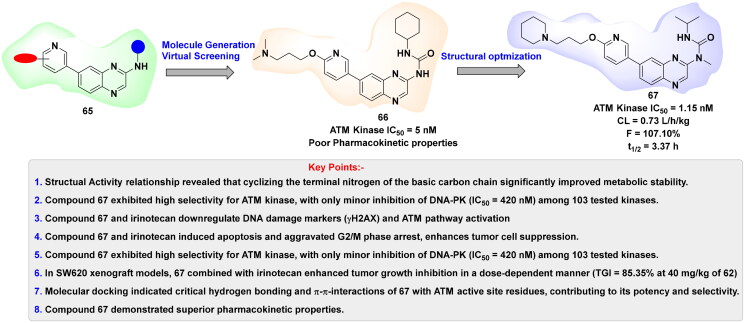
Quinoxalin urea derivatives as ATM inhibitors (the figure was drawn by the authors using Chemdraw software).

Deng et al. conducted a comprehensive lead optimisation study to develop potent ATM kinase inhibitors through a novel goal-oriented molecular design approach. The selection of the lead scaffold was guided by a multi-parametric strategy that incorporated various predictive factors, including Lipinski’s rule of five, synthetic approachability, drug-similarity, molecular dock scores, binding interactions, and pharmacokinetics profiles, with a focus on ligand similarity and target-specific structural features. This rational design approach culminated in the identification of a promising lead compound, which subsequently underwent extensive structural modifications aimed at mitigating its metabolic vulnerabilities. The primary objective of structural refinement was to reduce the hepatic clearance of the lead compound while preserving its potency and selectivity towards ATM kinase. As a result of these iterative optimisation efforts, compound **(67)** was identified, exhibiting a superior pharmacodynamic and pharmacokinetic profile (Figure 11). *In vitro* and *in vivo* assays highlighted the therapeutic potential of the compound **(67),** demonstrating its multifaceted anticancer effects. The compound **(67)** exhibited synergistic antitumor activity when combined with irinotecan in MCF-7 and SW620 cancer cell lines, significantly enhancing the cytotoxic response and exhibiting dose-dependently inhibit the ATM signalling pathway by reducing the expression levels of phosphorylated ATM and phosphorylated p21. Additionally, compound **(67)** effectively downregulated the expression of the DNA damage biomarker γH2AX in HCT116 cells, indicating its role in suppressing DNA damage signalling. Moreover, the compound **(67)** induced cell death by promoting cell cycle arrest at the G2/M phase, further supporting its potential as a targeted therapeutic agent against ATM-driven malignancies. Importantly, compound **(67)** demonstrated remarkable selectivity, as confirmed by kinase screening against a panel of 103 kinases. Furthermore, the optimised compound exhibited favourable pharmacokinetic properties, including reduced *in vivo* clearance, moderate half-life, high systemic exposure, and excellent oral bioavailability in Balb/c mice, with adequate permeability in Caco-2 cell assays. Notably, the combination of orally administered compound **(67)** with irinotecan (40 mg/kg) resulted in a significant reduction in both malignant tumour volume and weight in a human colon cancer SW620 xenograft model^309^.

**Figure 12.**
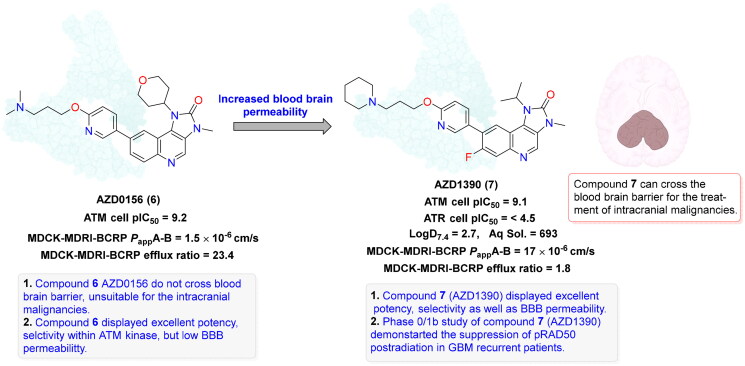
Discovery of novel AZD1390 (7) as an ATM kinase inhibitor with BBB permeability (the figure was drawn by the authors using Chemdraw software).

Pike et al. identified a novel, selective ataxia telangiectasia mutated kinase inhibitor that can penetrate the blood-brain barrier. According to the chemical architecture of previously synthesised clinical candidate AZD0156 **(6),** Pike and their co-workers developed a new ATM kinase inhibitor AZD1390 **(7)**. The strategy is intended to reduce the hydrogen bonding ability, basicity, and flexibility of the resulting scaffold. The authors used positron emission tomography to afford the combination of carbon-11 isotope to generate AZD1390 **(7)** which displayed good brain exposure in non-human primates (NHPs) compared to AZD0156 **(6)**. Thermo Fisher Scientific kinase panel was used, which displayed that AZD1390 **(7)** exhibited excellent selectivity over all the related kinases such as PI3Ka/mTOR/DNA-PK with the pIC50 values <4.9, <4.8, and <4.5, respectively. Moreover, AZD1390 **(7)** displayed an excellent pre-clinical pharmacokinetic profile in rats (CL/*V*ss/*T*_1/2_/%F = 16/3.0/2.4/74) and dogs (CL/*V*ss/*T*_1/2_/%F = 16/24/22/66). Additionally, the rat brain Kp, uu (0.33 ± 0.068) of AZD1390 **(7)** was determined by using the *in-vivo* K_p_ and *in-vitro* unbound fraction in the brain (*f* u,b = 0.010) and plasma (*f* u,*p* = 0.175) which displayed excellent pharmacokinetic with increased lipophilicity and *in-vitro* MDCKII-MDR1-BCRP efflux ratio of less than 2. Furthermore, a Phase 0/1b study of AZD1390 **(7)** demonstrated a post-radiation reduction in the pRAD50 levels in GBM recurrent patients. Overall, the authors reported the discovery of AZD1390 **(7)** as a CNS-penetrating ATM kinase inhibitor (Figure 12)^310^.

**Figure 13.**
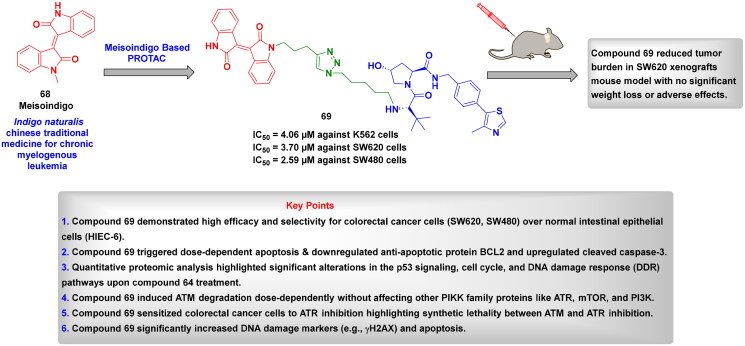
Meisoindigo-derived PROTAC as the ATM degrader (the figure was drawn by the authors using Chemdraw software).

In pursuit of developing innovative antitumor therapies, Liu et al. embarked on a drug discovery endeavour to assemble Meisoindigo-based degraders (PROTACS). Notably, Meisoindigo (Mei), a mono *N*-methyl derivative of indirubin **(68)**, is derived from *Indigo naturalis* (traditional Chinese medicine) and demonstrates efficacy against diverse malignancies^73^. Notably, this N-heterocyclic compound has been utilised in the treatment of chronic myeloid leukaemia (CML) in China for an extended period. Several precedents ascertained the impact of Meisoindigo on multiple oncogenic pathways (DNA biosynthesis, cell cycle arrest, and microtubule assembly)^311–314^. Impressed by the aforementioned, this medicinal chemistry campaign was initiated to elucidate the molecular target and unfold the mechanistic insights of Meisoindigo via its installation in the PROTAC template to generate chemical tools (degraders) capable of inducing targeted protein degradations via activation of ubiquitin − proteasome systems. The efforts culminated in the identification of a PROTAC that manifested magnificent potential to degrade ATM kinase in a ubiquitin − proteasome dependent manner in SW620 and SW480 cells (colorectal cell lines) as revealed by the outcome of DiaPASEF-based quantitative proteomic analysis. Western blot analysis ascertained the ATM degradation ability of compound **(69)** as downregulation of ATM expression levels was observed in SW620, SW480, and K562 cells, with compound **(69)** treatment. Also, an abrogation in the cell progression inhibitory effects of the compound **(69)** was observed in decreasing the ATM level via the use of shRNA technology. The amalgamation of the compound **(69)** and the ATR inhibitor **(21)** overwhelms the cells’ ability to manage accumulated DNA damage, resulting in catastrophic genomic instability and cell death. This dual-targeted strategy significantly increases DNA damage and apoptosis markers, such as γ-H2AX and cleaved-caspase 3. In both *in vitro* studies and xenograft mouse models, the combination therapy effectively reduced tumour growth without causing notable toxicity (Figure 13). By targeting the weaknesses of ATM-deficient cancer cells, this approach not only boosts the effectiveness of ATR inhibitors but also introduces a promising strategy to overcome chemoresistance, paving the way for more innovative and effective treatments for colorectal cancer^315^.

**Figure 14.**
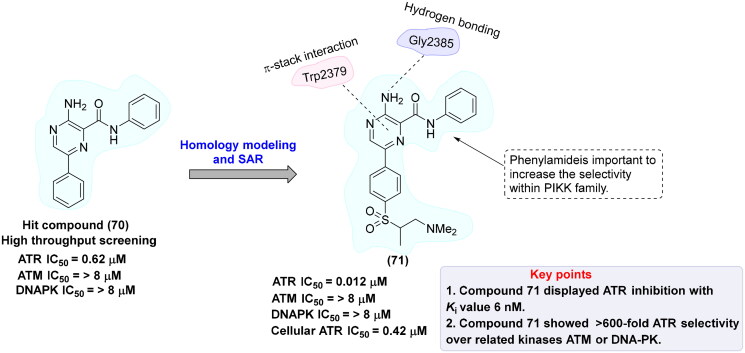
Development of potent and selective ATM and Rad3-related ATR kinase inhibitor (the figure was drawn by the authors using Chemdraw software).

In the quest to generate efficacious ATR inhibitors, Charrier et al. conducted high throughput screening and arrived at the chemical architecture of compound **70**. The homology modelling study of compound **70** led the research group to pinpoint the key interactions imperative for potency as well as selectivity towards ATR. Specifically, π-stack interactions between a tryptophan (Trp2379) with the aromatic group (position 6, pyrazine ring) were deduced to be critical. Building on this information, a structure-activity relationship was established that culminated in the recognition of compound (**71)** as a potent and selective ATR inhibitor that demonstrated a > 600-fold selectivity towards ATR over related kinases. Moreover, the outcome of the Caco2 study revealed that compound (**71)** demonstrated good aqueous solubility, lipophilicity (cLogP 3.0), and profile. Also, the ATR inhibitor elicited potent cell growth inhibitory effects against ATM pathway deficient cells and was lacking cytotoxicity towards normal cell lines. It was also observed that death exerted by DNA-damaging agents was significantly enhanced by compound (**71)** in a selective manner (only towards certain cancers and not on normal cells) (Figure 14).^148^

**Figure 15.**
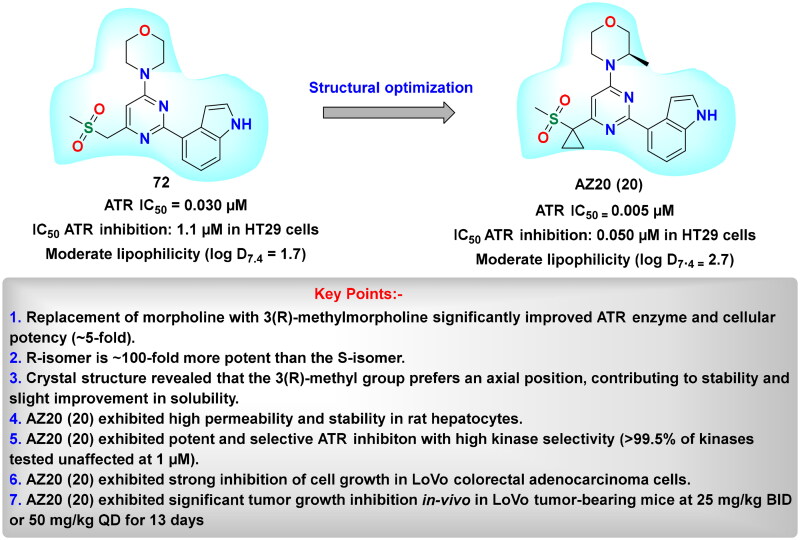
Discovery of potent and selective ATR inhibitor (AZ20) (the figure was drawn by the authors using Chemdraw software).

Foote *et al* selected a sulphonyl morpholino pyrimidine-based mTOR inhibitor **(72)** endowed with ATR inhibitory potential for their medicinal chemistry campaign to amplify its selectivity and potency as an ATR inhibitor. Noteworthy to mention that despite being endowed with an impressive ATR inhibitory profile and ability to inhibit the ATR-guided phosphorylation of Chk1 at serine-345, its low solubility is directed to the commencement of a structural optimisation program to fine-tune the physiochemical profile of compound **(72)**. Delightfully, the consequence of this study guided the identification of Compound **(20)** which displayed significant improvement in cellular potency compared to the lead compound **(72)**. Exhaustive explorations of Compound **(20)** led to promising revelations viz. (i) Compound **(20)** exerted inhibition of ATR immunoprecipitated from HeLa nuclear (IC_50_ of 5 nM); (ii) Compound **(20)** induced ATR guided phosphorylation of Chk1 (IC_50_ = 50 Nm, HT29 colorectal tumour cells; (iii) Compound **(20)** manifested selectivity towards ATR kinase; (iv) AZ20 **(20)** exhibited substantial mouse free drug exposure; (v) Compound **(20)** exerted remarkable inhibition in the tumour growth in Lovo xenografts (Figure 15)^316^.

**Figure 16.**
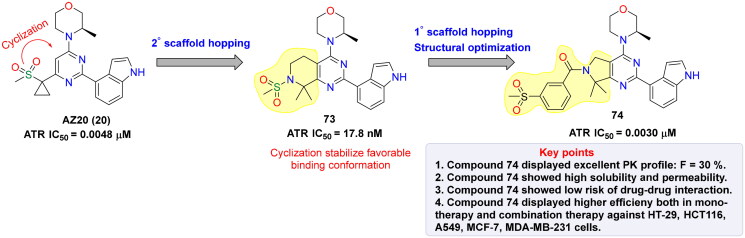
Discovery of pyrimidine based as ATR inhibitors (the figure was drawn by the authors using Chemdraw software).

Qi et al. conducted structural engineering of AZ20 **(20)**, an ATR inhibitor comprising of a pyrimidine skeleton substituted with (*R*)-3-methylmorpholinyl and indolyl functionality. The interaction profile of AZ20 **(20)** was carefully studied using the ATR protein homology model and key interactions were figured out. Notably, the binding of (*R*)-3-methylmorpholinyl functionality to kinase hinge and a hydrogen bond with Val 851 residue along with a water-mediated hydrogen bond between N–H of the indole and Asp 810 residue were pinpointed as critical interactions. The docking study also revealed that the solvent-exposed methylsulfonyl functionality of compound **(20)** extended to the solvent-susceptible region and can be subjected to a structural alteration program. With this background, the research group employed a structural rigidification approach to confine the development of the sulphonyl side chain. Resultantly, a compendium of pyrimidine derivatives was generated and evaluated as ATR inhibitors. The efforts culminated in the identification of a compound **(74)** endowed with striking ATR inhibitory activity and remarkable cell growth inhibitory effects against ATM-deficient cancer lines. Further investigations revealed that compound **(74)** demonstrated, **(**i) concentration-dependent colony formation inhibition and migration in LoVo cells, (ii) synergistic anticancer efficacy (cell studies) with AZD-1390 **(7),** cisplatin, oxaliplatin, and PARP inhibitor, Olaparib against a panel of cancer cell lines, (iii) mild liver microsomal stability and appropriate pharmacokinetic profile (Figure 16)^317^.

**Figure 17.**
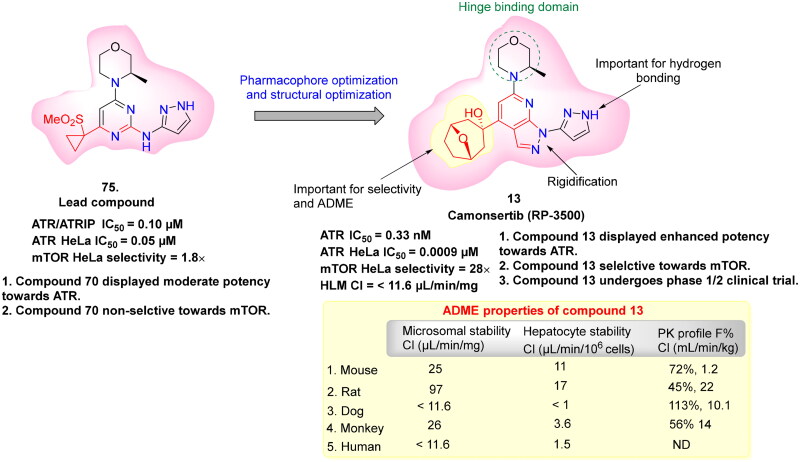
Discovery of the novel potent and selective ATR inhibitor camonsertib (the figure was drawn by the authors using Chemdraw software).

Black et al. designed and synthesised a novel ATR kinase inhibitor to treat genetically selected solid tumours. The authors utilised a pharmacophore and structurally driven optimisation approach which directed to the discovery of a potent and selective compound with excellent ADME properties. Specifically, the lead ATR inhibitor **(75)** was used for the structural rigidification strategy to preserve critical hydrogen bonding interaction. Delightfully, the approach culminated in compound **(13)** (Camonsertib, RP-3500) with a rigidified scaffold that displayed excellent ATR inhibition with an IC_50_ value of 0.33 nM and selectivity over mTOR kinase, compared to compound (**75)** (moderate potency and non-selective over mTOR). *In-vitro* metabolic stability in human liver microsome assay indicated that compound **(13)** (Camonsertib-RP-3500) displayed a clearance of < 11.6 (µL/min/mg) which was acceptable compared to rat, dog, and monkey. The *in-vitro* cell-based assay included DNA-PK, ATM, mTOR, and PI3Kα in LoVo and HT-29 cells displayed that the compound **(13)** (Camonsertib, RP-3500) was 30-fold selective towards ATR over mTOR and > 2000-fold selective over DNA-PK, ATM and PI3Kα. Moreover, Camonsertib (RP-3500) **(13)** demonstrated potent antitumor activity *in vivo* studies, particularly in xenograft models harbouring DNA repair deficiencies, such as mutations in ATM and BRCA1. A 3-days-on/4-days-off dosing regimen was effective in mitigating anaemia, a frequently observed adverse effect of ATR inhibitors while maintaining therapeutic efficacy. Notably, Camonsertib **(13)** exhibited synergistic effects with PARP inhibitors, including niraparib and olaparib, enabling enhanced antitumor efficacy at reduced doses and minimising toxicity through intermittent dosing schedules (Figure 17). These preclinical findings have informed the design of clinical trials, where intermittent dosing strategies have shown the potential to reduce severe anaemia and improve overall patient outcomes^143^.

**Figure 18.**
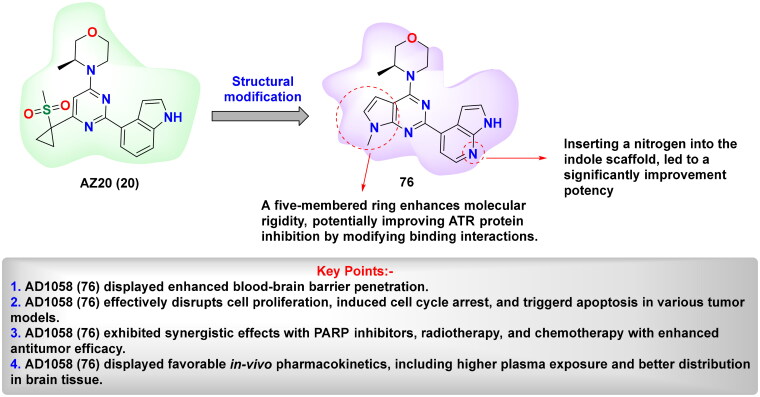
Discovery of potent ATR inhibitor (AD1058) (the figure was drawn by the authors using Chemdraw software).

Wang et al. conducted a structural engineering endeavour on **AZ20 (20)** to furnish AD1058 **(76),** a potent, selective, and brain-penetrant ATR inhibitor for the treatment of advanced carcinoma. *In vitro* assays demonstrated that AD1058 **(76)** effectively inhibits ATR with an inhibitory concentration of 1.6 nM, outstanding the performance of earlier compound AZ20 **(20).** Notably, AD1058 **(76)** exhibits significant blood-brain barrier (BBB) penetration, achieving a brain-plasma ratio of 31% which is a remarkable improvement over AZ20 **(20),** thereby making it a strong candidate for treating brain metastases and leptomeningeal metastases. Additionally, AD1058 **(76)** has robust anticancer activity across a widespread extent of carcinoma cell lines, particularly those with ATM deficiencies. Thus, it is conceived that compound (**76)** leverages synthetic lethality to enhance its therapeutic impact. Also, AD1058 **(76)** disrupts the cell cycle by inducing S-phase arrest and promotes apoptosis more effectively than previous ATR inhibitors. Furthermore, compound (**76)** exposes cancer cells to DNA-damaging agents, including PARP inhibitors, chemotherapy, and, ionising radiotherapy, displaying pronounced synergistic effects in combination therapies. These attributes make AD1058 (**76)** especially promising in overcoming resistance mechanisms in PARP inhibitor-resistant cancers. In terms of physiochemical properties, AD1058 (**76)** demonstrates high plasma protein binding (99.5%) and excellent oral bioavailability. It also achieves high area-under-the-curve (AUC) exposure and superior distribution to brain tissues compared to previous ATR inhibitors, further validating its strong CNS-targeting capabilities (Figure 18). In the safety evaluations, AD1058 (**76)** showed no significant toxicity, apart from minor changes in platelet counts and serum creatinine levels, which highlights its therapeutic potential. Additionally, AD1058 **(76)** exhibits minimal off-target activity against other kinases, ensuring a safer and more selective therapeutic profile^318^.

**Figure 19.**
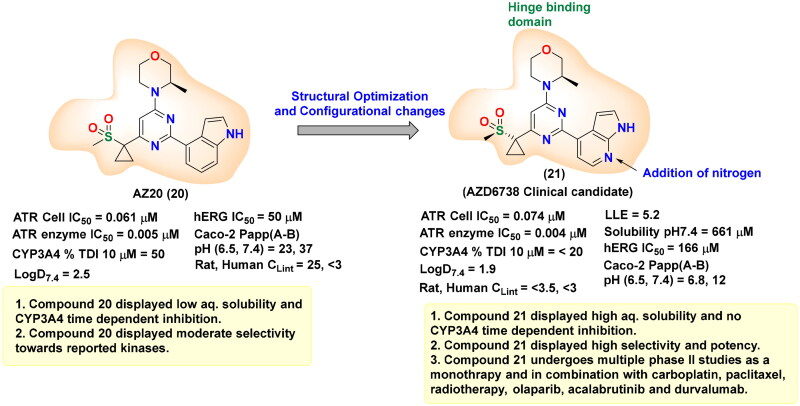
Discovery of novel potent ATM and Rad3-related ATR kinase inhibitor (the figure was drawn by the authors using Chemdraw software).

Foote and their co-workers previously synthesised potent and selective ATR kinase inhibitor AZ20 **(20).** Unfortunately, **AZ20** demonstrated poor aqueous solubility and displayed an elevated menace of drug-drug interactions. To increase the aqueous solubility, structural optimisation, and configurational changes were performed on compound AZ20 **(20)** which directed to the discovery of compound (**21) (AZD6738),** a 7-azaindole sulfoximine derivative. Delightfully, **21** (**AZD6738)** displayed high water solubility. Also, compound (**21)** displayed excellent ATR inhibitory potency with an IC_50_ value of 0.004 µM. AZD6738 **(21)** displays high kinase selectivity, with minimal off-target activity against related PIKK-family kinases such as mTOR, DNA-PK, and ATM in cell-based assays. *In-vivo* xenograft studies revealed that compound **21** was well tolerated once daily at 50 mg/kg (QD) in mice. Additionally, (**21)** exerted significant TGI inhibition in the xenograft model at a dose of 25 mg/kg (BD). Moreover, compound (**21)** displayed excellent physicochemical properties with high solubility in aqueous media and simulated gastric fluid as well as fasted state simulated intestinal fluid. *In-vivo* pharmacokinetic evaluation revealed that compound (**21)** showed low to moderate clearance, moderate volume of distribution, and good bioavailability in rodents and dogs **(**Figure 19). Noteworthy to mention that compound (**21)** is currently under phase-II clinical study as a mono-therapy together with carboplatin, paclitaxel, olaparib, acalabrutinib, and durvalumab for the treatment of cancer^319^.

**Figure 20.**
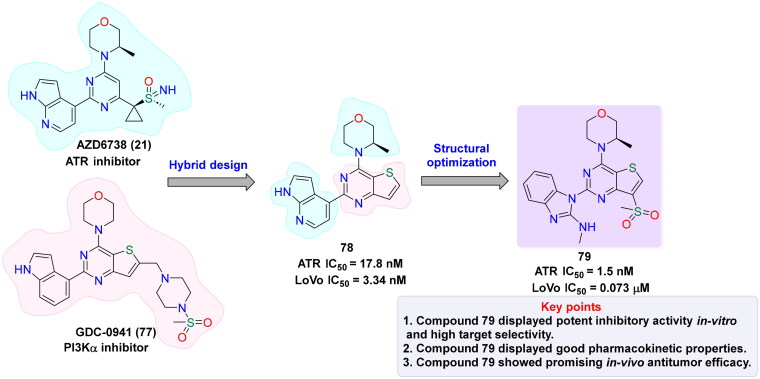
Discovery of potent and selective Thieno[3,2-*d*]pyrimidine derivatives (the figure was drawn by the authors using Chemdraw software).

In pursuit of supplementing the preclinical synthetic bank of ATR inhibitors with efficacious entries that can be translated to higher-stage explorations and eventually to clinical trials, Duan et al. embarked on a medicinal chemistry campaign and conducted a lead modification of a hybrid scaffold comprising of pharmacophoric features of GDC-0941 (PI3Kα inhibitor) **(77)** and 7-azaindole moieties **(78)** (ATR inhibitor). Notably, the hybrid scaffold (**78)** manifested an ATR inhibitory profile with IC_50_ values of 17.8 nM. Additional structural optimisation directed to the identification of a thieno[3,2-*d*]pyrimidine derivative compound **(79)** endowed with remarkable ATR inhibitory activity (ATR IC_50_ = 1.5 nM) as well as antiproliferative effects against LoVo cells (ATR IC_50_ = 1.5 nM). The compound **(79)** also demonstrated selectivity towards ATR kinase, impressive *in vivo* pharmacokinetic properties, and reasonable antitumor efficacy in the LoVo xenograft model (Figure 20). Gladly, the cocktail of compound **(79)** and AZD1390 **(21)** induced synthetic lethality in HT-29 cells^320^.

**Figure 21.**
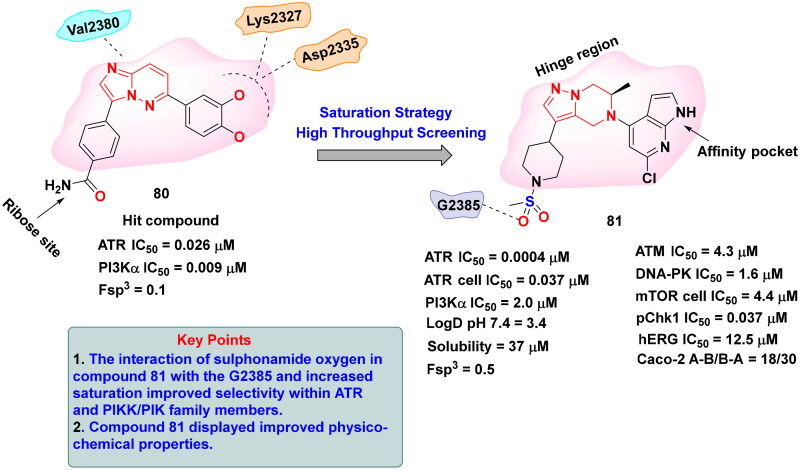
Discovery of new potent and selective tetrahydropyrazolo [1,5‑*a*]pyrazines as ATR Inhibitors (the figure was drawn by the authors using Chemdraw software).

Barsanti et al. reported the design and synthesis of potent and selective tetrahydropyrazolo[1,5-a]pyrazine (THPP) derivatives as ATR inhibitors, specifically targeting cancer cells harbouring p53 mutations, which rely on ATR for survival. These inhibitors were designed to rebuild sensitivity to DNA-damaging agents by effectively inhibiting ATR activity. The discovery of these inhibitors began with high-throughput screening (HTS) assays that utilised gemcitabine-induced Chk1 phosphorylation to identify initial hits. Among these, compound (**80)** (Lead Compound) demonstrated ATP-competitive inhibition of ATR but exhibited suboptimal selectivity. To optimise the initial hit, a comprehensive structure-activity relationship (SAR) study was conducted. Key modifications to the molecular scaffold included increasing the fraction of sp³-hybridized carbons (Fsp³) and incorporating a 6-chloro substituent on the azaindole core. These modifications yielded compound (**81)**, the highly potent ATR inhibitor in the series, with an IC_50_ of 0.0004 μM against ATR and significantly reduced activity against PI3Kα (IC_50_ = 2.0 μM). Notably, compound **(81)** demonstrated a markedly improved kinase selectivity profile, minimising off-target interactions, including the inhibition of the hERG ion channel, a known contributor to cardiotoxicity. Further structural analysis revealed that the methylsulfonylpiperidine moiety of the compound (**81)** engages with non-conserved residues in the ATR kinase’s lower hinge and ribose-binding pocket. This unique binding interaction emphasises its selectivity over additional phosphatidylinositol 3-kinase-related kinases (PIKKs), including ATM, DNA-PK, and mTOR. The selective inhibition of ATR is a significant attribute of its therapeutic potential in oncology, where ATR inhibitors enhance the efficacy of DNA-damaging agents by exploiting tumour cells’ heightened replication stress and compromised DNA repair mechanisms. Despite its promising pharmacological profile, compound (**81)** exhibits time-dependent inhibition of the cytochrome P450 enzyme CYP3A4, raising concerns about potential drug-drug interactions. Mechanistic studies of the compound (**81)** identified reactive metabolites formed on the piperazine core as the primary cause of this liability (Figure 21). Efforts to mitigate time-dependent inhibition through structural modifications, such as core alterations and deuterium substitution, achieved partial success but did not fully eliminate the issue. Nonetheless, the risk associated with CYP3A4 inhibition is considered manageable within combination therapy regimens, where lower doses of ATR inhibitors are sufficient to achieve chemo sensitisation, thereby reducing overall exposure and minimising the potential for adverse interactions^321^.

**Figure 22.**
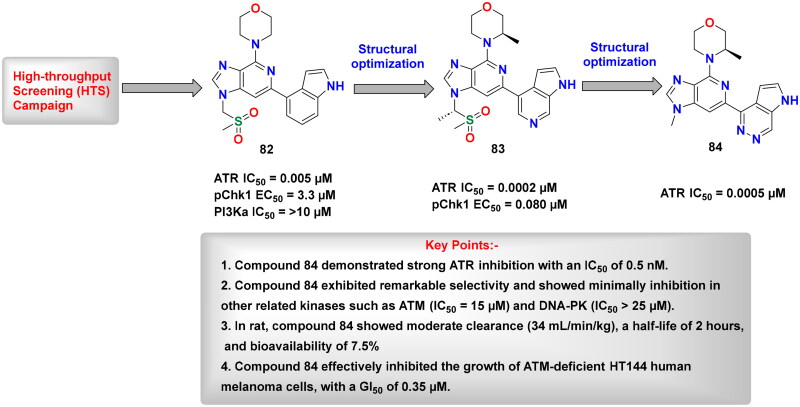
Discovery of potent azabenzimidazoles based as ATR inhibitor (the figure was drawn by the authors using Chemdraw software).

Paul A. Barsanti and collaborators successfully designed and synthesised a series of novel, potent, and highly selective azabenzimidazole (ABI)-based ATR inhibitors. A hypothesis-driven siRNA screening revealed that ATR inhibition exhibits synthetic lethality in the context of ATM deficiency, establishing a robust genetic rationale for the therapeutic application of ATR inhibitors as standalone agents. To explore this, a high-throughput screening (HTS) campaign was conducted, which initially identified ATR inhibitors. However, many of the initial hits exhibited poor kinase selectivity, notably displaying off-target activity against PI3K and related kinases. Subsequent efforts employing a combination of virtual screening and targeted HTS led to the discovery of novel chemotypes with improved lipid kinase selectivity. Among these, a morpholino-imidazopyrimidine was identified as a selective ATR inhibitor **(**Figure 22). Despite its promising selectivity profile, the compound exhibited suboptimal physicochemical properties, which hindered further development. Redesigning the core structure to an azabenzimidazole scaffold resulted in compound **(82)**, which demonstrated significant improvements in solubility, stability, and *in vivo* pharmacokinetics. Further optimisation to enhance binding affinity and selectivity led to the development of compound **(83)**, which displayed exceptional *in vitro* potency and selectivity. However, compound **(83)** suffered from poor *in vivo* bioavailability and significant liabilities, including hERG ion channel inhibition and time-dependent CYP3A4 inhibition. To address these challenges, structural modifications were attempted, replacing the azaindole core with a pyrrolo[2,3-d]pyridazine scaffold. This approach yielded compound (**84)**, which successfully mitigated hERG and CYP3A4-associated liabilities while retaining strong potency and selectivity. Compound (**84)** exhibited significant efficacy in suppressing the proliferation of ATM-deficient HT144 melanoma cells and displayed a favourable pharmacokinetic profile, positioning it as a promising agent for *in vivo* validation of the synthetic lethality paradigm between ATM loss and ATR inhibition^322^.

**Figure 23.**
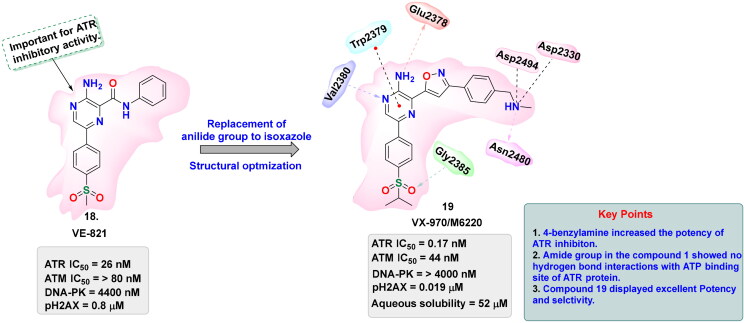
Discovery of first in class (VX-970, M6620) as ATR inhibitor (the figure was drawn by the authors using Chemdraw software).

Knegtel et al. rationally designed a first-in-class Rad3-related ATR kinase inhibitor compound (**19)** for the treatment of cancer. Initial optimisation was carried out on previously reported ATR inhibitor compound (**18)** (**VE-821)** which was developed by Charrier *et al* and displayed moderate potency and poor physicochemical properties [239]. Leveraging the chemical architecture of **18** (2-aminopyrazine derivative), optimisation of intra- and intermolecular polar attachments for the development of a novel ATR kinase inhibitor was performed which directed to the development of compound (**19)** (VX-970, M6620). Notably, compound (**19)** demonstrated a magnificent ATR kinase inhibitory profile with an IC_50_ value of 0.17 nM. It was postulated that the incorporation of an isoxazole moiety within the structural framework of compound 19 contributed to the enhancement of its physicochemical properties. Encouragingly, compound (**19)** demonstrated an impressive pharmacokinetic profile with Cl = 26 ml min^−1 ^kg^−1^, V*ss* = 21 L/kg, and t_1/2_ = 11.6 h, respectively. Moreover, compound (**19)** displayed > 200-fold selectivity towards ATR over cytochrome P450, Cyp3A4, 2C9, and 2D6. Also, compound (**19)** exhibited >100-fold selectivity towards ATR over related kinases. Furthermore, it was observed that compound (**19)** effectively sensitised the HCT116 colorectal cell line to the DNA cross-linking agent cisplatin, with a concentration of < 50 nM. A molecular docking assay showed that compound (**19)** fits well in the active site ATR pocket and displayed important hydrogen bond interactions with the unique residues Gly2385, Asn2480, and Asp2494 (Figure 23). Overall, the authors identified a novel ATR inhibitor currently being evaluated in Phase I/II clinical trials in combination with various DNA-damaging agents and PARP inhibitors for the treatment of solid malignancies^323^.

**Figure 24.**
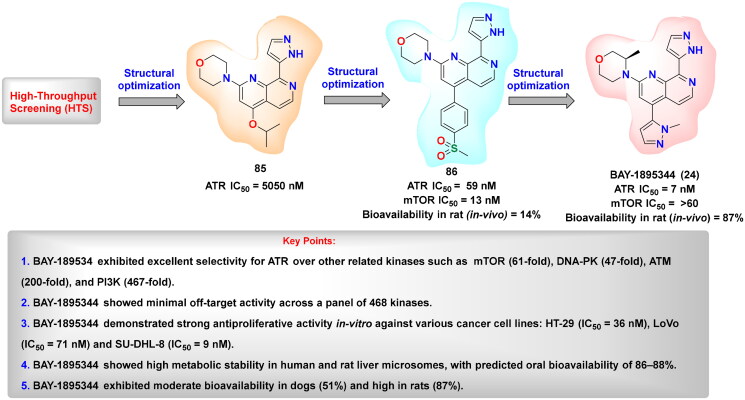
Discovery of BAY-1895344 as an orally active, selective, and potent ATR inhibitor (the figure was drawn by the authors using Chemdraw software).

Lucking and co-workers reported the discovery of BAY-1895344 **(24)** as an orally active, selective, and potent ATR inhibitor. At the outset of the endeavour, careful analysis of the binding pattern of the model compound AZ20 **(20)** within the active site of ATR was done. Resultantly, it was deduced that the making of hydrogen bonds by morpholine oxygen and indole N-H group within the hinge region of the site was important for the inhibitory activity of **AZ20**. With this information in hand, compound (**85)** (IC_50_ = 5050 nM) was synthesised first and evaluated, wherein it showed inferior ATR inhibition than AZ20 **(20)** (IC_50_ = 3 nM). Further, optimisation culminated in compound (**86)** (IC_50_ = 59 nM, having naphthydrine at 7-position) as a potent ATR inhibitor endowed with striking antiproliferative activity against HT-29 and LoVo cells. Albeit, compound (**86)** manifested good enzymatic and cellular activities, limitations such as poor oral bioavailability, poor PK profiles, and hERG liability were associated with its treatment. Thus, further structural optimisation of (**86)** was carried out which revealed that the presence of pyrazole at the 8-position of the naphthydrine core was necessary for optimal binding within the active site of ATR protein. Continued structural interrogation involving variation at the 4-position of the core led to the identification of BAY-1895344 **(24)** which displayed excellent inhibitory activity against ATR (IC_50_ = 7 nM). Also, BAY-1895344 **(24)** exhibited a promising antiproliferative activity profile against the panel of selected cell lines (IC_50_ = 9 – 160 nM), improved solubility at pH 6.5, and moderate metabolic stability in dog and mouse microsomal cells with predicted oral bioavailability of 86% after 60 min of incubation. Also, low blood clearance rates were observed for BAY-1895344 **(24)** in rats and dogs In addition, no CYP liabilities, and lower plasma protein bindings were observed with BAY-1895344 **(24)** in humans and rats. Furthermore, BAY-1895344 **(24)** elicited potent *in-vivo* antitumor efficacy in human cancer xenografts. The *in vivo* study also revealed that BAY-1895344 **(24)** had the potential to increase pATR and increase pH2AX. Combined with carboplatin, BAY-1895344 **(24)** produced synergistic effects in platinum-resilient ATM protein low-articulating CR5038 human CRC patient-derived xenograft (PDX) model in NOD/SCID mice (Figure 24). In addition, BAY-1895344 **(24)** exerted synergistic antitumor efficacy with olaparib (PARP inhibitor)^324^.

**Figure 25.**
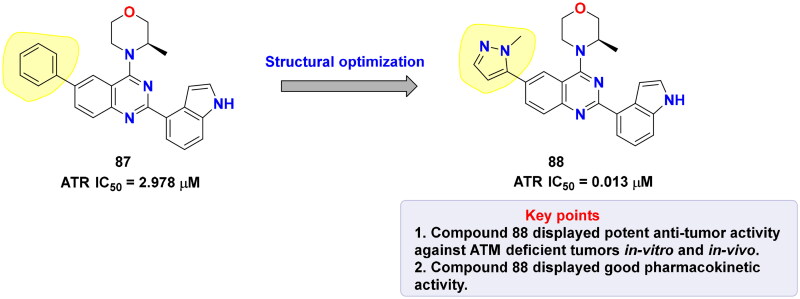
Discovery of highly selected ATR kinase inhibitor (the figure was drawn by the authors using Chemdraw software).

Linli Li et al. conducted a medicinal chemistry campaign and identified a hit compound (**87)** with moderate ATR inhibition (IC_50_: 2.978 µM) through initial screening of a kinase inhibitor library. A detailed structure-activity relationship (SAR) study was further performed to enhance potency, focusing on three regions of the quinazoline scaffold. First, substitution at the 6^th^ position was attempted with a methylpyrazole group that led to significant improvement in potency, resulting in compound (**88)** (IC_50_: 0.013 µM). Further, placement of an indole group at the 2^nd^ position and (R)-3-methylmorpholine at the 4^th^ position enhanced both activity and selectivity as evidenced by the activity profile of the resulting compound (**88)**. Molecular docking studies revealed that compound (**88)** formed key interactions with ATR kinase, including a hinge-binding hydrogen bond between its 3-methylmorpholine group and VAL-851. Also, interactions of its indole group with ASP-810 and hydrophobic residues such as LEU-807, ILE-932, and PHE-934 were observed. Additionally, its methyl pyrazole group engaged in π-π interactions with TRP-850, hydrophobic interactions with MET-772, TRP-850, and MET-922, and a bond with GLN-859, while its methyl group stabilises the conformation in a hydrophobic pocket, boosting ATR selectivity. *In vitro* study results indicate that compound (**88)** was endowed with potent and selective cytotoxicity against ATM-deficient cell lines (LoVo) and exerted minimal effects on ATM-proficient lines. Compound (**88)** effectively inhibited ATR signalling pathways, increased DNA damage markers such as γH2AX, and suppressed tumour cell proliferation and migration, as confirmed by colony formation and migration assays. *In vivo* studies further highlighted its antitumor efficacy, with compound (**88)** achieving up to 72.8% tumour growth inhibition in LoVo xenograft mouse models at a dose of 100 mg/kg, without notable toxicity. Mechanistic studies validated its ATR inhibition and DNA damage induction in treated tumours (Figure 25). Moreover, compound (**88)** exhibited favourable pharmacokinetic properties, including good bioavailability and moderate clearance, making it a promising candidate for further development as a targeted cancer therapy^325^.

**Figure 26.**
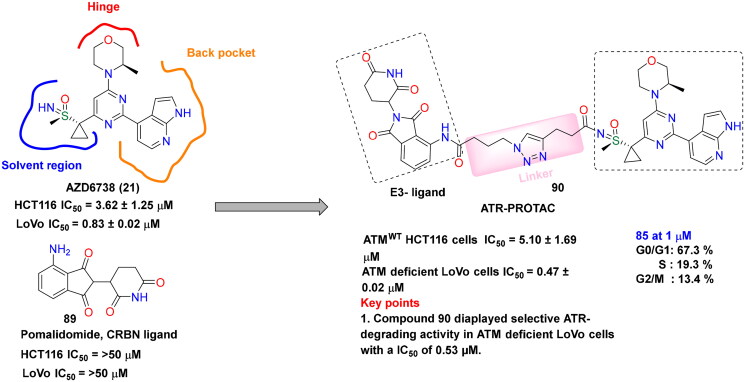
Discovery of the first Ataxia Telangiectasia and Rad3-related (ATR-PROTAC) (the figure was drawn by the authors using Chemdraw software).

Huang et al. utilised the chemical architecture of AZD6738 **(21),** a magnificent ATR inhibitor as a warhead to furnish ATR targeting degraders (PROTAC). At the outset, the research group conducted a molecular docking study of AZD6738 **(21)** which revealed that the solvent domain exposed sulfoximine group is an appropriate site for appendage with the linker to connect it to the E3 ligase ligand. Notably, von Hippel–Lindau (VHL) and cereblon (CRBN) were pinpointed as the ligands for the E3 ligase. Comprehensive profiling of the furnished PROTAC_S_ led to the identification of compound (**90)** endowed with potent and selective ATR degrading activity in ATM-deficient LoVo cells (DC_50_ = 0.53 μM). Also, compound (**90**) manifested cell growth inhibitory activities against ATM-deficient LoVo cells (IC_50_ value of 0.47 µM). *In vivo* studies revealed that compound (**90)** possessed significant tumour growth inhibitory activity (as a single agent as well as in combination) in the LoVo xenograft mouse model (Figure 26). Moreover, a correlation between the antitumor effects and the ATR degrading ability of compound (**90)** was established through the *in-vivo* studies^326^.

**Figure 27.**
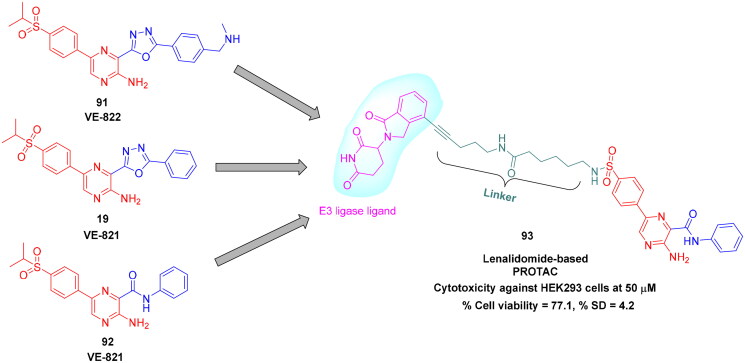
Discovery of novel first-in-class proteolysis targeting chimaera (PROTACs) (the figure was drawn by the authors using Chemdraw software).

Leveraging the structural assemblage of previously synthesised highly potent and selective ATR kinase inhibitors, Alfayomy et al. designed and synthesised first-in-class proteolysis targeting chimaera (PROTACs) for the Ataxia Telangiectasia and Rad3-related kinase. Of all the synthesised compounds, the lenalidomide-based PROTAC (**93)** was found to be the highly potent ATR degrader in MIA PaCa-2 cancer cell lines. The plasma protein binding and plasma stability studies were carried out in which compound (**93)** displayed 89.4 ± 0.3% plasma protein binding and 75% plasma stability. The non-enzymatic stability assay results indicated that compound (**93)** was chemically stable and did not yield any degradation product after 72h. Also, the *in-vitro* non-radioactive assay was performed over the ATR/ATRIP complex in which compound (**93)** displayed weaker inhibition with an IC_50_ value of > 10 μM. Moreover, compound (**93)** also induced degradation of the MIA PaCa-2 cells at 2 μM through the ubiquitin-proteasome-system. Furthermore, flow cytometry assay and annexin-V/PI staining results revealed that compound (**93)** induced cell death in MIA PaCa-2 cells. Additionally, it was observed that compound (**93)** did not show any cytotoxic effects on HEK293 cells at an excessive concentration of 50 μM, ascertaining it selective cytotoxic action towards the cancer cells (Figure 27). Overall, the authors highlighted the first-in-class discovery of proteolysis targeting chimaera (PROTACs)^327^.

### Medicinal chemistry endeavours on DNA-PK inhibitor

**Figure 28.**
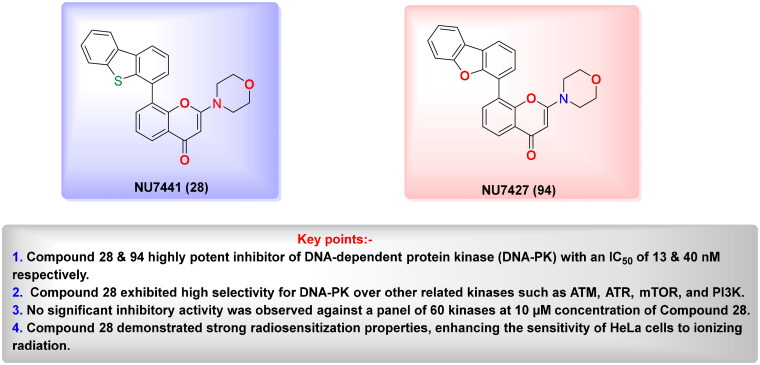
Chromen-4-one based DNA-dependent protein kinase (DNA-PK) inhibitors (the figure was drawn by the authors using Chemdraw software).

Hardcastle and colleagues developed a series of highly impressive chromen-4-one-based inhibitors targeting DNA-dependent protein kinase (DNA-PK). Structure-activity relationship (SAR) analysis indicated that 2-morpholinyl substituent was critical for DNA-PK inhibitory activity due to its essential hydrogen bonding interactions within the ATP-binding site. Substitutions at the 6- and 7-positions of the chromen-4-one scaffold typically resulted in diminished activity, whereas modifications at the 8-position, particularly with hydrophobic and aromatic groups such as dibenzothiophene and dibenzofuran, were well-tolerated and enhanced potency. Owing to the aforementioned structural optimisation endeavours, compounds (**28)** and (**94)** were identified as highly potent DNA-PK inhibitors, with (**28)** manifesting superiority in terms of exceptional potency (IC_50_ = 13 nM) and selectivity. Notably, compound (**28)** demonstrated over 100-fold selectivity for DNA-PK relative to closely related kinases, including ATM, ATR, PI3K, and mTOR, and exhibited minimal off-target activity against a panel of 60 kinases, underscoring its specificity. In functional assays, compound (**28)** significantly sensitised HeLa cells to ionising radiation and the chemotherapeutic agent, etoposide. At sub-micromolar concentrations, it potentiated cytotoxic effects, yielding dose modification factors (DMFs) of 1.3 and 2.5 at concentrations of 0.2 µM and 0.5 µM, respectively, in radiation sensitisation experiments (Figure 28). Importantly, compound **(28)** displayed no inherent cytotoxicity at concentrations up to 10 µM, further supporting its potential as a therapeutic agent^328^.

**Figure 29.**
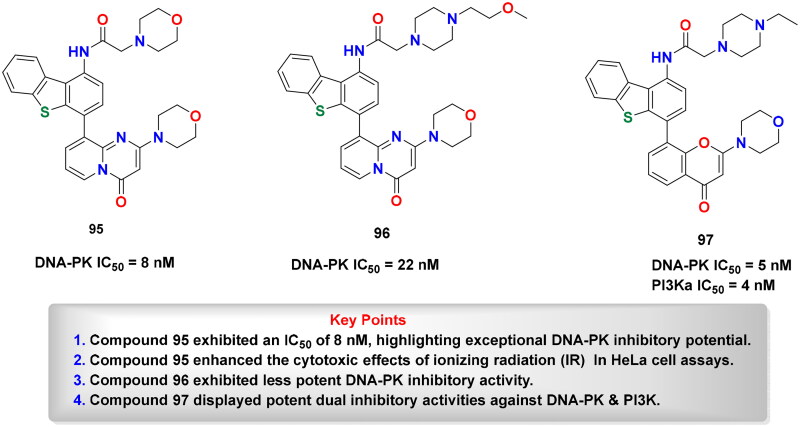
Discovery of pyridopyrimidin-4-one derivatives based DNA-PK inhibitors (the figure was drawn by the authors using Chemdraw software).

In an extension of the previously identified DNA-PK inhibitor (**28)**, Celine Cano and colleagues synthesised an innovative series of DNA-PK inhibitors by substituting the chrome-4-one core scaffold with quinolin-4-one and pyridopyrimidin-4-one heterocycles. Water-solubilizing substituents were strategically introduced at 1-position of the dibenzothiophen-4-yl and dibenzofuran-4-yl functionalities to enhance pharmacokinetic and physicochemical properties. Structure-activity relationship (SAR) analyses revealed that pyridopyrimidin-4-one derivatives exhibited significantly higher potency than their quinolin-4-one counterparts, with dibenzothiophen-4-yl substitutions imparting higher efficacy relative to dibenzofuran-4-yl analogs. Pyridopyrimidin-4-one derivatives demonstrated exceptional nanomolar inhibition of DNA-PK, with compound (**95)** achieving an IC_50_ of 8 nM. In cellular assays employing HeLa cells, compound (**95)** markedly potentiated the cytotoxicity of ionising radiation (IR), and other derivatives exhibited dose modification ratios (DMRs) exceeding 10 at a concentration of 0.5 μM. While compounds with greater intrinsic potency generally achieved higher DMR values, parameters such as aqueous solubility and cellular permeability were found to modulate overall efficacy^329^. In another study, the same research group developed a series of dibenzothiophene- and 4-chromone-based small molecules as dual inhibitors of PI-K and DNA-PK. This study aimed to improve the pharmacokinetic properties of compound (**28)** while preserving its biological activity. Using *in silico* homology modelling, the researchers synthesised four series of dibenzothiophene derivatives with 4-chromone moieties and a polar substituent. *In vitro* assay identified compound (**97)** as a potent DNA-PK inhibitor, with an IC_50_ value of 5 nM. This molecule showed no activity against other kinases, including ATM, ATR, and mTOR. Additionally, compound (**97)** exhibited strong inhibitory effects on head and neck squamous tumour cells and significantly reduced colony formation (Figure 29). The mechanism of action of compound (**97)** was determined to involve the inhibition of replication fork formation^330^.

**Figure 30.**
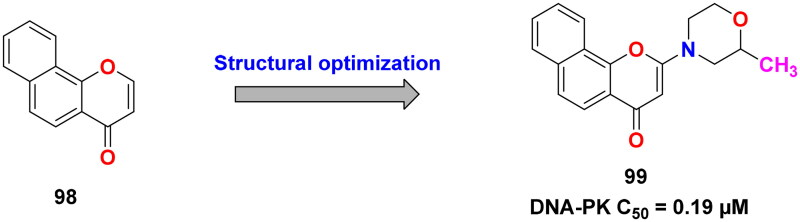
Discovery of benzopyranone-based DNA-pk inhibitors (the figure was drawn by the authors using Chemdraw software).

Griffin et al. designed, synthesised, and evaluated a series of DNA-PK inhibitors based on benzopyranone and pyrimidoisoquinolin-4-one structures. A total of 72 small molecules were synthesised and evaluated for their ability to inhibit DNA-PK, as well as their selectivity for associated kinases such as ATM, ATR, mTOR, and PI3. Among these, compound (**99)** was identified as a highly aggressive and selective DNA-PK inhibitor, with an IC_50_ of 0.19 µM. Compound (**99)** exhibited ATP-competitive inhibition, with a Ki value of 24 nM, indicating a strong binding affinity for the ATP-binding site of the kinase. Structure-activity relationship studies revealed the importance of the 2-position substituent on the inhibitor scaffold. Only 2–(2′-methylmorpholino) groups were well-tolerated, with other modifications causing a significant loss of activity. This suggested a tightly constrained binding pocket, where precise placement of functional groups is crucial for effective kinase inhibition. Additionally, compound (**99)** demonstrated excellent selectivity, showing minimal inhibition of other kinases within the phosphatidylinositol 3-kinase-related kinase (PIKK) family. *In vitro*, the assays further confirmed the therapeutic potential of compound (**99)** as it sensitised HeLa tumour cells to ionising radiation, significantly enhancing radiation-induced cytotoxicity. At a concentration of 5 µM, compound (**99)** increased the dose modification factor to 2.3 at 10% survival, indicating a substantial improvement in the effectiveness of radiation therapy (Figure 30)^331^.

**Figure 31.**
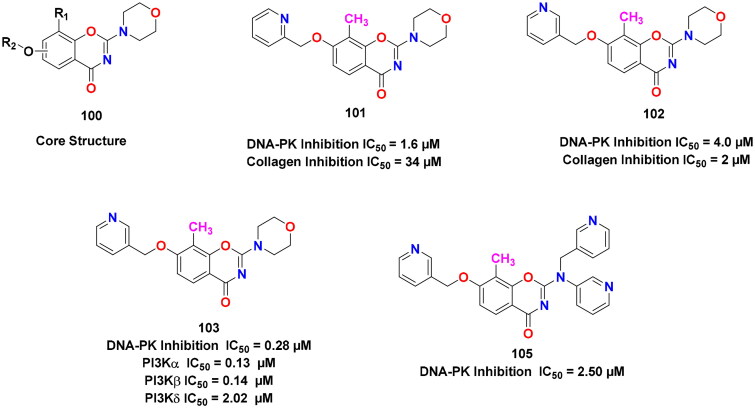
Novel series of benzoxazine-based compounds for DNA-PK inhibitor (the figure was drawn by the authors using Chemdraw software).

The PI3K (phosphoinositide 3-kinase) enzyme family is significant to various intracellular signalling mechanisms that control key physiological processes like cell growth, proliferation, adhesion, and survival. Due to these roles, PI3Ks are considered important targets for treating conditions such as cancer, thrombosis, and immunoinflammatory diseases. They also play a role in DNA repair through the DNA-PK, a member of the PI3K family. In pursuit of capitalising on the aforementioned disclosures, Jasim Al-Rawi and his team designed and synthesised a novel series of benzoxazine-based compounds to inhibit DNA-PK and different PI3K isoforms. They developed 31 new compounds and assessed their effects on collagen-induced platelet aggregation. Among these, (**101)** and (**102)** were found to be the most potent compounds. Notably, compound (**101)** exerted strong DNA-PK inhibition with an IC_50_ value of 1.6 µM and adduct **(102)** demonstrated effective collagen inhibition with an IC_50_ value of 2 µM^332^. Also, Jasim Al-Rawi and colleagues further developed two new series of compounds featuring 2-morpholino-substituted-1,3-benzoxazines and 2-(N-substituted-(pyridin-3-ylmethyl) amino)-substituted-1,3-benzoxazines. Each compound was evaluated for DNA-PK inhibition and antiplatelet effects, with compounds **(103)** and **(104)** exhibiting potent DNA-PK inhibition, showing IC_50_ values of 0.28 µM and 2.50 µM, respectively. Additionally, PI3K inhibition studies showed compound **(103)** excellent PI3K inhibitory profile with IC_50_ values for PI3K isoforms as follows: PI3Kα = 0.13 μM, PI3Kβ = 0.14 μM, PI3Kγ = 0.72 μM, and PI3Kδ = 2.02 μM. These Studies indicate that benzoxazine analogs exhibit significant anticancer potential, primarily through the inhibition of DNA-dependent protein kinase (DNA-PK), as a key component of the DNA damage response (Figure 31). Consequently, targeting DNA-PK holds therapeutic promise for enhancing the sensitivity of cancer cells to DNA-damaging agents, including chemotherapeutic drugs and radiotherapy^333^.

**Figure 32.**
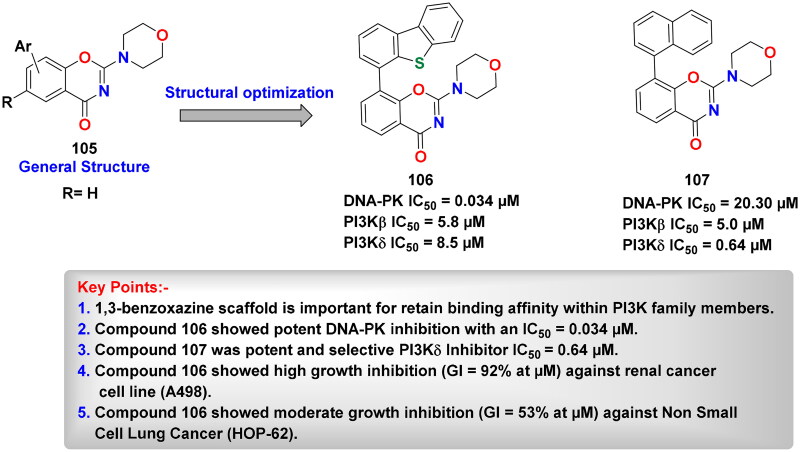
Discovery of dual DNA-PK and PI3K inhibitors (the figure was drawn by the authors using Chemdraw software).

1,3-benzoxazine scaffold has been identified as a promising platform for developing more selective ligands that target specific PI3K isoforms. Given the aforementioned, Rawi and colleagues synthesised a series of DNA-PK and PI3K inhibitors based on 6-aryl, 8-aryl, and 8-aryl-6-chloro-2-morpholino-1,3-benzoxazine backbones, focusing on optimising DNA-PK inhibition alongside selectivity for PI3K isoforms. An initial screening of thirty-one compounds was conducted at a concentration of 10 μM, with IC_50_ values subsequently determined for compounds demonstrating more than 50% inhibition. To optimise the initial hit, a comprehensive structure-activity relationship (SAR) study was conducted and yielded compound (**106)** demonstrated potent DNA-PK inhibition (IC_50_ = 0.034 μM) with high selectivity over Class I PI3K isoforms (all IC_50_ values > 5 μM). Additionally, compound (**107)** showed strong activity against the PI3Kδ isoform (IC_50_ = 0.64 μM) and moderate inhibition of PI3Kβ (IC_50_ = 5.0 μM), resulting in 32-fold selectivity for PI3Kδ over DNA-PK. In cancer cell viability assays, both compounds (**106)** and (**107)** showed significant antiproliferative activity across multiple cell lines. Compound (**106)** exerted 90% inhibition in the renal cancer cell line A498, while compound **(107)** demonstrated a maximum inhibition of 54% against the non-small cell lung cancer line HOP-92 (Figure 32)^334^.

**Figure 33.**
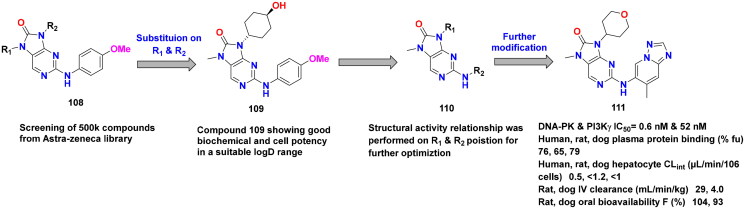
Discovery of AZD7648 potent and selective DNA-PK inhibitor (the figure was drawn by the authors using Chemdraw software).

Goldberg et al. discovered a highly potent and selective DNA-dependent protein kinase (DNA-PK) inhibitor, **AZD7648 (21),** through a screening process involving over 5,000 compounds from AstraZeneca’s synthetic library. During this screening, they identified a promising starting compound **(109),** featuring a 7,9-dihydro-8H-purin-8-one bicyclic core with trans-4-hydroxycyclohexyl substituent, which exhibited good biochemical and cellular potency. To understand the binding interactions of compound **(109)** within the DNA-PK active site, the research group employed various binding models, analysing the compound’s crystal structure in the DNA-PK binding pocket. These analyses revealed that aniline NH forms a hydrogen bond with the backbone carbonyl of Cys666. Notably, in a homologous PI3Kγ structure, the aniline group exhibited a reversed binding orientation within the pocket, where it formed a hydrogen bond with Glu880 and Val882. Further structure-activity relationship (SAR) studies involved the incorporation of additional structural elements (triazolopyridine and tetrahydropyran) into the general core structure. Encouragingly, the efforts led to the identification of compound **(111)** which demonstrated high biochemical and cellular potency, good permeability, and favourable crystalline solubility. It also showed promising pharmacokinetic properties, with low clearance and high oral bioavailability. Kinome profiling confirmed that compound **(111)**exhibited minimal off-target activity across the protein kinome, with only weak activity against PI3Kα/γ lipid kinases. *In vivo,* compound **(111)** acted as a potent radiosensitizer, effectively enhancing the effects of ionising radiation and synergizing with doxorubicin, both of which induce double-strand DNA breaks (DSBs). In xenograft and patient-derived xenograft models, these combinations led to marked tumour volume reductions (Figure 33). Importantly, compound **(111)** also showed some single-agent efficacy, a novel finding for selective DNA-PK inhibitors, as it achieved tumour regressions in both ATM-deficient and ATM wild-type models. Further studies demonstrated that compound **(111)** induced sustained tumour regression when combined with the PARP inhibitor (Olaparib) in an ATM knockout xenograft model^335^.

**Figure 34.**
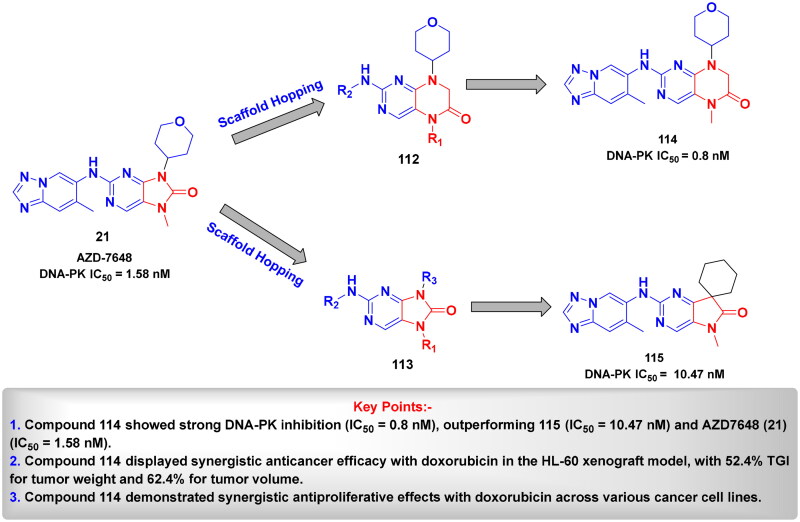
Discovery of 7,8-dihydropteridine-6(5H) based DNA-PK inhibitors (the figure was drawn by the authors using Chemdraw software).

Jianjun chen et al. rationally designed and developed two series of novel 7,8-dihydropteridine −6(5H) based DNA-PK inhibitors as potent anticancer agents through scaffold hopping strategy and evaluated their biological effects. Most of the compounds demonstrated strong inhibitory effects in DNA-PK inhibitory assays, with IC_50_ values below 300 nM. In particular, two compounds (**114** and **115**) were pinpointed as the most promising of the two series. Compound **(114)** from the first series demonstrated exceptional potency, with an IC_50_ of 0.8 nM, surpassing the well-known inhibitor AZD-7648 **(21)** (IC_50_ = 1.58 nM). **Compound (115)** from the second series showed lower potency, with an IC_50_ of 10.47 nM, making it less effective than compound **(21),** however, the compound was endowed with promising drug-like properties *in vitro* and favourable pharmacokinetic profiles for oral administration *in vivo.* Further, compound **(114)** reduced γH2AX expression levels, and it exhibited synergistic antiproliferative effects across various cancer cell lines when used alongside doxorubicin in mechanistic studies. In toxicity assessments, compound **(114)** did not induce bone marrow suppression, as granulocyte and lymphocyte counts remained comparable to the control group. Molecular simulation analyses revealed that compound **(114)** formed a stable complex with DNA-PK, establishing interactions with key amino acid residues, including Ile3938, Met3929, Ala3931, Val3930, and Asn3926, along with electrostatic interactions. Notably, compound **(114)** combined with the DNA double-strand break-inducing agent doxorubicin showed significant anticancer effects in an HL-60 xenograft model, achieving tumour growth inhibition (TGI) rates of 52.4% in tumour weight and 62.4% in tumour volume (Figure 34)^336^.

**Figure 35.**
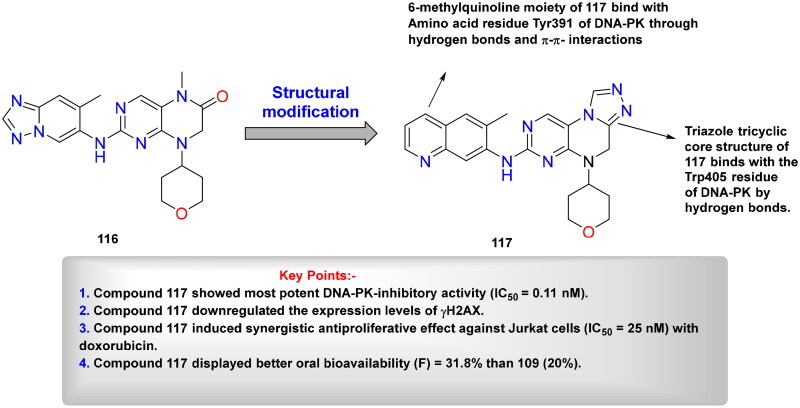
Discovery of heterotricyclic based as DNA-dependent kinase (DNA-PK) inhibitors (the figure was drawn by the authors using Chemdraw software).

Although compound **(114)** demonstrated strong anti-DNA-PK activity, its oral bioavailability was relatively low (F = 20.8%). To enhance compound **(114)** anticancer efficacy and pharmacokinetic profile, Chen *et al* optimised it through a tricyclic ring design approach to improve its oral bioavailability, and its potential to enhance cancer immunotherapy. The team synthesised 14 heterotricyclic DNA-PK inhibitors and assessed their biological activity. Many of these compounds displayed strong enzymatic activity with impressive IC_50_ values in DNA-PK biochemical assays. Notably, compound **(117)** emerged as the most potent, with a DNA-PK IC_50_ of 0.11 nM. Compound **(117)** also effectively reduced γH2A.X expression and exhibited optimal synergistic antiproliferative and immunomodulatory effects with doxorubicin against Jurkat cells (IC_50_ = 25 nM) and in CT26 and B16–F10 tumour-bearing mouse models. Additionally, compound **(117)** showed improved pharmacokinetic properties, including a higher oral bioavailability (F = 31.8%) than the previously reported compound (Figure 35)^337^.

**Figure 36.**
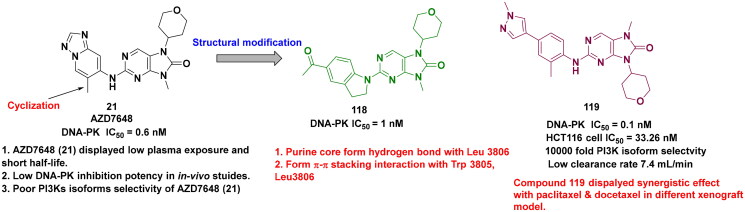
Discovery of AZD7648 derivative as DNA-PK inhibitors (the figure was drawn by the authors using Chemdraw software).

Several DNA-PK inhibitors have demonstrated significant therapeutic effects when combined with chemo- or radiotherapy in clinical studies. However, both clinical and preclinical studies have revealed several pharmacokinetic challenges with these inhibitors, primarily due to the complex role of DNA-PK in cancer progression. Addressing this need, Lijuan Chen and colleagues designed and synthesised a new series of small-molecule inhibitors guided by molecular docking. Among these, cyclic compound **(118)** demonstrated good inhibitory potency as compared to compound **(21).** Notably, its unique cyclic structure includes a fatty chain that links the secondary amine of the compound **(21)** to a neighbouring methyl group, equalising the molecule’s conformation. Compound **(118)** maintained significant potency even without the hydrogen bond (2.5 Å) between the secondary amine and core chain oxygen of Glu3804 amino acid. These modifications also reduced activity against PI3Kγ, a member of the phosphoinositide 3-kinase (PI3K)-related protein kinase family. Further structural modifications to increase selectivity against PI3Kγ while enhancing or preserving DNA-PK activity led to a series of compounds that featured the splitting of triazolopyridine moiety of compound **(21)** in two monocyclic rings and linking the parts via a rotatable C-C bond. Among these, compound **(119)**, having methylpyrrole group, showed outstanding inhibitory activity against DNA-PK, with an impressive IC_50_ value of 0.1 nM. It also exhibited a minimal *in vitro* excellent clearance rate and limited toxicity in normal cells. Moreover, compound **(119)** showed at least a 10,000-fold selectivity over four PI3K variants and ATR. In the H460 xenograft model, compound **(119)** in combination with radiotherapy significantly enhanced the antitumor effect compared to compound **(21)** with radiotherapy. Combination **(119)** with docetaxel liposomes yielded better efficacy than compound **(21)** in the BT474 xenograft model under similar conditions. Similarly, compound **(119)** combined with paclitaxel markedly enhanced antitumor efficacy compared to paclitaxel alone in a xenograft model. Noteworthy to mention that administering oral compound **(119)** at 7.5 mg/kg alongside intravenous paclitaxel at 10 mg/kg resulted in a 78.79% tumour inhibition rate (Figure 36). Moreover, compound **(119)** showed no significant changes in the body weight of mice over the 28-day treatment, suggesting a favourable safety profile for compound **(119)** in combination with paclitaxel^338^.

**Figure 37.**
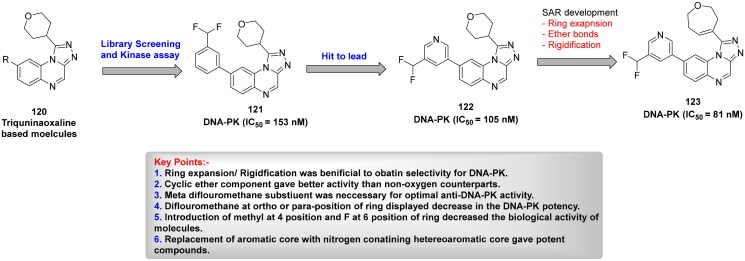
Discovery of potential antitumor agent as DNA-PK inhibitor for conjugated therapy (the figure was drawn by the authors using Chemdraw software).

Berger et al. disclosed Compound **(123)** as a new and highly selective inhibitor of DNA-dependent protein kinase and a promising conjugate for antitumor therapies. Initially, the Bayer library was screened, and compound **(122)** (having a triquinoxaline nucleus) showing higher selectivity for DNA-PK was chosen as a hit. Different substituents at position 8 of the triquinoxaline moiety were placed and assessed for kinase activity, wherein compound **(122)** showed the highest activity. The results also highlighted that difluoro-methyl at the meta-position of the substituent at the 8^th^ position was optimal for DNA-PK selectivity. Further, the identified lead, compound **(122)**, was modified through rigidification or ring expansion, which led to the discovery of compound **(123)**, endowed with higher potency for DNA-PK (IC_50_ = 81 nM) and PI3Kβ (IC_50_ = 117 nM) inhibition and good antiproliferative activity against HT-29 cells (IC_50_ = 358 nM). Also, compound **(123)** showed moderate stability in rat and human hepatocytes, low metabolic stability in mouse hepatocytes, no CYP liabilities, and 49% plasma protein binding. The *in vivo* pharmacokinetic evaluation of compound **(123)** showed moderate blood clearance in rats. In mice, the rate was high, bioavailability was low (22%) and half-life was 4 h. Finally, the antitumor activity of the compound was evaluated in monotherapy and combination with PSMA-TTC using an androgen-responsive LNCaP model (Figure 37). The results showed that in monotherapy, compound **(123)** showed mild antitumor activity on oral dosing, while in combination, the antitumor effect increased significantly, and the body weight of treated animals was significantly improved^339^.

## Conclusion and perspective

DNA damage response (DDR) kinases, including DNA-PK, ATR, and ATM as therapeutic targets for antitumor drug design have garnered significant attention from researchers over the last decade. The literature covered in this review perspicuously demonstrates the sincere efforts invested by the medicinal chemistry fraternity to load the armoury of inhibitors endowed with modulatory ability towards key DNA damage response kinases. Delightfully, the attempts made in this direction have culminated in the advancement of several inhibitors of ATM, ATR, and DNA-PK to clinical stage explorations. Amongst the DNA damage response kinase inhibitors, ATR inhibitors appear to be leading the race in terms of advanced-stage clinical investigation. Notably, ATR inhibitor, AZD-6738 is undergoing phase 3 clinical trials for NSCLC while VE-822 and BAY-1895344 are in phase 2 clinical-stage evaluation for oesophageal and colorectal cancer, respectively. An encouraging development in the field of DNA damage response kinase inhibitors at the clinical level is their advancement for clinical studies in glioblastoma and neuroblastoma. Noteworthy to mention that, ATM inhibitor, WSD-0628, and DNA-PK inhibitor, CC-115 are being profiled as anti-glioblastoma agents in clinical studies. To add on, clinical stage assessment is going on for DNA-PK inhibitor LY294002 in neuroblastoma. Given, the complexities associated with GBM treatment viz. lack of chemotherapeutics, the existence of glioma stem cells, and lack of CNS penetrating ability of small molecule inhibitors, promising outcomes of DNA damage response kinase inhibitors in brain tumours would expand the list of therapeutic options for the treatment of glioblastoma/neuroblastoma. Excitingly, the patent literature also presents some interesting and tractable entries (ATM, ATR, and DNA-PK inhibitors) exhibiting antitumor efficacy in diverse malignancies, however, detailed mechanistic studies are required to be conducted to assess their ability to exert conclusive therapeutic benefits in cancer.

The most strategic attempts of the drug discovery teams have been made in pre-clinical studies where the medicinal chemist has invested remarkable efforts in generating a compendium of small molecule structural assemblages targeting DNA repair response kinase via robust drug design strategies. Resultantly, numerous efficacious ATM, ATR, and DNA-PK inhibitors have been pinpointed that are yearning for *in-depth* investigations for ascertainment of their candidature to exert conclusive benefits in cancer. Important to mention, that the research groups hitherto working in the direction of small molecule inhibitor discovery for DNA damage response kinases have majorly relied on two strategies viz. (i) lead modification involving scaffold hopping and strategic placement of substituents and (ii) new scaffold construction using diverse heterocyclic units. While both strategies aimed to generate adducts with amplified ATM/ATR/DNA-PK modulatory ability, in numerous instances, lead modification studies were solely designed to negate the physicochemical/pharmacokinetic liabilities of the potent chemical architectures. One of the exemplary studies to overcome the aforementioned constraints was conducted by Balaram et al. In the study, a pragmatically installed cinnoline scaffold outwitted the pharmacokinetic hindrances of the quinolone counterpart. Eventually, the outcome of the endeavour culminated in a potent ATM inhibitor endowed with favourable pharmacokinetic properties, including long half-lives, high oral bioavailability, and good solubility^266^. Similarly, there are preclinical precedents for ATR kinase and DNA-PK inhibitors where a lead modification approach was employed to improve the solubility/metabolic stability/bioavailability of the lead scaffold. Indeed, endeavours conducted at the preclinical level to generate ATR inhibitory chemical architectures reflect the skilfulness of medicinal chemists in the most perspicuous manner in narrowing the limitations associated with the previously reported ATR inhibitors. Notably, drug discovery sleuths in the past invested significant efforts in the development of ATR inhibitors. Though the endeavours paid dividends in the context of yielding efficacious ATR inhibitors, selectivity towards ATR was pinpointed as the major constraint hurdling the advancement of such inhibitors. Prudent drug design strategies were employed to solve the aforestated issue, leading to the furnishment of effective as well as selective ATR inhibitors as antitumor agents. Likewise, several logically designed medicinal chemistry campaigns were also conducted for the generation of tractable DNA-PK inhibitors at the preclinical level. In particular, one of the studies performed by Astra Zeneca employed a holistic approach for DNA-PK inhibitor design. In this endeavour, a lead structural template was identified at the outset and then its binding interactions were figured out through various binding models to analyse the compound’s crystal structure in the DNA-PK binding pocket. After the attainment of information on the binding pattern of the lead compound, further structural optimisation was carried out resulting in a compound that demonstrated high biochemical and cellular potency, good permeability, and promising pharmacokinetic properties^335^. Working on similar lines, several medicinal chemistry campaigns were conducted that culminated in a plethora of pharmacodynamically and pharmacokinetically impressive small molecule DNA-PK inhibitors deserving enough to be explored exhaustively. It is important to mention that medicinal chemists have not confined themselves merely to the design of inhibitors and have extended their campaigns towards the fabrication of degraders (PROTAC). Meisoindigo-based PROTAC manifesting substantial ATM kinase degrading potential in a ubiquitin − proteasome dependent manner in SW620 and SW480 cells (colorectal cell lines)^315^ and AZD6738 based PROTAC eliciting potent and selective ATR degrading activity in ATM-deficient LoVo cells along with significant tumour growth inhibitory activity (LoVo xenograft mouse model)^326^ serves as prototype examples of DNA damage response kinase targeting PROTACS.

Given the remarkable efforts invested in the medicinal chemistry endeavours on DNA damage response kinases, it is anticipated that continued attempts in this direction towards the design of inhibitors as well as degraders will sail the boat of DNA damage response kinase inhibitors as cancer therapeutics in terms of enhancing their clinical utility. Also to mention that the combination therapy involving drug cocktails of ATM/ATR/DNA-PK inhibitors with other targeted agents/chemotherapeutic agents has shown optimistic results indicating that such inhibitors have the potential to sensitise cancer cells, thereby enhancing tumour regression. To capitalise on this, dual inhibitor drug design to assemble DNA-damage response kinase bifunctional inhibitors can be exercised that might prove to be beneficial in the context of cementing the candidature of such adducts as antitumor agents. Overall, the integration of structural insights, mechanistic understanding, and in vivo assessments offers significant potential for advancing DDR-targeted therapies, with the prospect of improving therapeutic outcomes across diverse cancer types.

## Data Availability

The authors confirm that the data supporting the findings of this study are available within the article.
